# Synthesis of 3,4,5-trisubstituted isoxazoles in water via a [3 + 2]-cycloaddition of nitrile oxides and 1,3-diketones, β-ketoesters, or β-ketoamides

**DOI:** 10.3762/bjoc.18.47

**Published:** 2022-04-22

**Authors:** Md Imran Hossain, Md Imdadul H Khan, Seong Jong Kim, Hoang V Le

**Affiliations:** 1 Department of BioMolecular Sciences and Research Institute of Pharmaceutical Sciences, School of Pharmacy, University of Mississippi, Mississippi 38677, USA; 2 Natural Products Utilization Research Unit, United States Department of Agriculture, Agricultural Research Service, University of Mississippi, Mississippi 38677, USA

**Keywords:** environmentally friendly, furoxans, 1,2,5-oxadiazole 2-oxides, trifluoromethyl-substituted isoxazoles, 3,4,5-trisubstituted isoxazoles

## Abstract

Herein we report a method for the synthesis of 3,4,5-trisubstituted isoxazoles in water under mild basic conditions at room temperature via a [3 + 2]-cycloaddition of nitrile oxides and 1,3-diketones, β-ketoesters, or β-ketoamides. We optimized the reaction conditions to control the selectivity of the production of isoxazoles and circumvent other competing reactions, such as O-imidoylation or hetero [3 + 2]-cycloaddition. The reaction happens fast in water and completes within 1–2 hours, which provides an environmentally friendly access to 3,4,5-trisubstituted isoxazoles, an important class of structures found in numerous bioactive natural products and pharmaceuticals. Additionally, we optimized the reaction conditions to produce trifluoromethyl-substituted isoxazoles, a prevalent scaffold in biomedical research and drug discovery programs. We also proposed a plausible mechanism for the selectivity of the [3 + 2]-cycloaddition reaction to produce 3,4,5-trisubstituted isoxazoles. Not to be overlooked are our optimized reaction conditions for the dimerization of hydroximoyl chlorides to form furoxans also known as 1,2,5-oxadiazole 2-oxides, a class of structures with important biological activities due to their unique electronic nature and coordination ability.

## Introduction

Isoxazoles are a privileged class of five-membered heterocycles, which are found in numerous bioactive natural products [1–2] and synthetic small molecule drugs [3–4], and are used as important precursors for the synthesis of β-hydroxycarbonyl compounds and γ-amino alcohols [1]. Isoxazoles, appearing in 33 patents from the year 2016 to 2018 [3], are an important drug class due to their wide range of biological activities, such as anticancer [5], antibiotic [6–7], antimicrobial [8], antifungal [9], and anti-inflammatory [10]. Therefore, new methods to develop efficient, high-yielding, and green routes to isoxazoles are always highly desirable.

**Figure 1:**
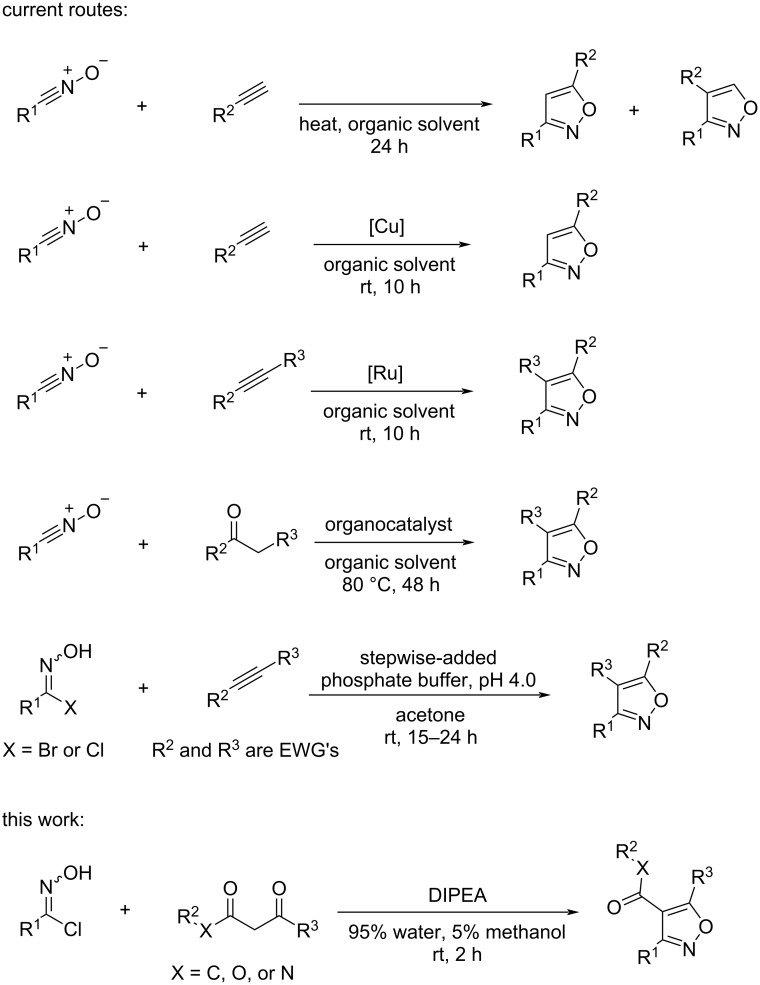
Routes to isoxazoles.

New approaches to 3,4,5-trisubstituted isoxazoles, particularly to the ones that leverage diverse chemical libraries, are exciting due to a limited number of suitable methods. One current route is the cycloaddition of alkynes and nitrile oxides performed in organic solvents at elevated temperatures (Figure 1) [11–16]. In this method, while 3,5-disubstituted isoxazoles can be accessed from terminal alkynes, 3,4,5-trisubstituted isoxazoles require a high degree of substitution on non-terminal alkynes to activate them for a decent yield of the isoxazole products, thus limiting the scope of the substrates in this method. In addition, this method requires high heat and produces very poor regioselectivity of the products [17–18]. The addition of copper catalysts in this route can help the reaction proceed at room temperature and improve both the regioselectivity and yields of the isoxazoles. However, these catalysts only work for the reaction with terminal alkynes and only produce 3,5-disubstituted isoxazole products (Figure 1) [19–20]. The synthesis of 3,4,5-trisubstituted isoxazoles from highly substituted non-terminal alkynes does not proceed with copper catalysts at room temperature. As an alternative, the usage of ruthenium(II) catalysts enables the reaction to proceed smoothly at room temperature and produces high yields and regioselectivity for both, 3,5-disubstituted and 3,4,5-trisubstituted isoxazoles (Figure 1) [21–22]. Similarly, palladium catalysts were used for the electrophilic intramolecular cyclization of alkynes and aldoximes to produce 3,4,5-trisubstituted isoxazoles, but the scope of the substrates of the method was limited as the substituted 2-alkyne-1-one *O*-methyl oximes needed to be synthesized independently [23]. While ruthenium(II) and palladium catalysts are useful, they are expensive and environmentally unfriendly.

The dehalogenation of hydroximoyl chlorides in the presence of a strong base to generate nitrile oxides and a follow-up cycloaddition with 1,3-diketones, β-ketoesters or β-ketoamides are a commonly used 2-step route to 3,4,5-trisubstituted isoxazoles [24–25]. Xiao Zhou et al. recently reported a direct access to 3,4,5-trisubstituted isoxazoles via an enolate-mediated 1,3-dipolar cycloaddition of β-functionalized ketones with nitrile oxides using organocatalysts (Figure 1) [26–27]. This enolate-mediated cycloaddition, however, requires long reaction time in organic solvents at high temperatures.

Our current work was inspired by a recent report by Kesornpun et al., in which a cycloaddition of nitrile oxides and alkenes or alkynes was carried out in 0.1 M phosphate buffer under weakly acidic conditions (pH 4.0) at room temperature for 15–24 h and generated 3,5-disubstituted isoxazolines and 3,4,5-trisubstituted isoxazoles in good yields (Figure 1) [28]. Notably, no metal catalysts were used in this method. The development of organic reactions in water not only is environmentally friendly, but also finds significant applications in biological systems (e.g., click reactions, bio-conjugation, and bio-orthogonal chemistry). One of such applications is ligation chemistry, in which “click” chemistry through a [3 + 2] biorthogonal cycloaddition between nitrile oxide and strained alkenes has been used to achieve the high-density functionalization of oligodeoxyribonucleotides [29]. However, the alkenes need to be highly strained in order for this reaction to occur without the need for an additional metal catalyst. As an alternative to the mild acidic conditions, we herein report a method for the synthesis of 3,4,5-trisubstituted isoxazoles in water under mild basic conditions at room temperature via a [3 + 2]-cycloaddition of nitrile oxides and 1,3-diketones, β-ketoesters, or β-ketoamides (Figure 1). No additional metal catalyst was used.

## Results and Discussion

**Figure 2:**
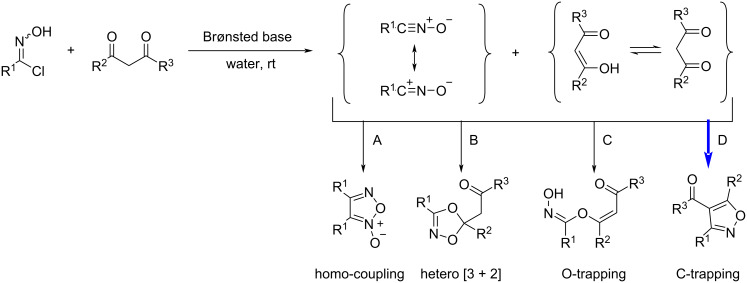
Possible products of the reaction between nitrile oxides and 1,3-diketones. Path D (C-trapping) produces 3,4,5-trisubstituted isoxazoles.

The reason why we investigated the reaction under mild basic conditions was because nitrile oxides are often generated by dehalogenation of hydroximoyl chlorides in situ under basic conditions or by dehydration of nitroalkanes [30]. So we were wondering whether we could take advantage of that. There are many possible products formed through the reaction between nitrile oxides and 1,3-diketones (Figure 2), such as the homo-coupled product (dimerization) of the nitrile oxides (Figure 2, path A) and dioxazoles through a hetero [3 + 2] cyclization as another possibility [30] (Figure 2, path B). In addition, there are competing reactions leading to the O-trapping product and the C-trapping product of the nitrile oxides (Figure 2, paths C and D). We were interested in the optimization of the reaction conditions leading to the isoxazole products (path D, Figure 2). For this purpose, we chose 4-fluoro-*N*-hydroxybenzimidoyl chloride (**1a**) and 1-phenylbutane-1,3-dione (**2a**) (Table 1) as the starting materials and a water–methanol mixture as the aqueous reaction medium. A series of organic and inorganic bases was screened at room temperature in various combinations of water–methanol mixtures and other solvents. The results are summarized in Table 1.

**Table 1:** Optimization of the reaction conditions for the synthesis of 3,4,5-trisubstituted isoxazoles **3**.^a^

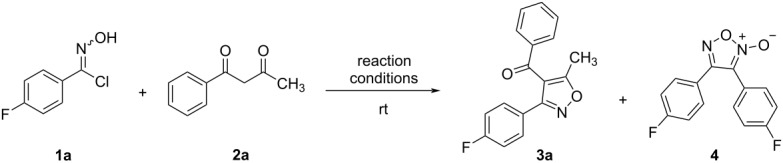					

Entry	Base	Solvents	Time (h)	Yield (%)	

				**3a**	**4**

1	DBU	5% water, 95% methanol	18	–	–
2	(*S*)-proline	5% water, 95% methanol	18	–	–
3^b^	NaHCO_3_	98% water, 2% methanol	3	14	55
4^b^	Na_2_CO_3_	98% water, 2% methanol	3	52	33
5	TEA	98% water, 2% methanol	2	54	22
6	TEA	5% water, 95 % methanol	2	68	19
7	TEA	CH_2_Cl_2_	2	50	28
8	TEA	isopropanol	2	57	30
9	DIPEA	CHCl_3_	1	43	52
10	DIPEA	CH_2_Cl_2_	1	17	81
11	DIPEA	5% water, 95 % methanol	1	89	–
12	DIPEA	95% water, 5% methanol	1	98	–
13^c^	DIPEA	2% water, 98 % methanol	5	95	–
14	DIPEA (10 mol %)	95% water, 5% methanol	2	–	70
15	–	5% water, 95 % methanol	2	–	95
16^d^	DIPEA (10 mol %)	phosphate buffer (pH 7.4), 5% methanol	2	7	76

^a^Unless otherwise noted, the reactions were performed with 0.5 mmol of **1a**, 0.5 mmol of **2a**, and 3 equivalents of base in 15 mL of the indicated solvents at room temperature for the indicated time. Yields are calculated from NMR spectra of the crude product using acetonitrile as the internal standard. ^b^Four equivalents of NaHCO_3_ and Na_2_CO_3_. ^c^Two equivalents of DIPEA and 45 mL of the solvent mixture. ^d^10 mol % of DIPEA. TEA = triethylamine, DIPEA = *N*,*N*-diisopropylethylamine, DBU = 1,8-diazabicyclo(5.4.0)undec-7-ene.

In our first attempt, DBU or (*S*)-proline in 5% water, 95% methanol yielded a complex mixture of products that were not easy to distinguish (Table 1, entries 1 and 2). The use of NaHCO_3_ in 98% water, 2% methanol after 3 hours, produced 14% of the expected 3,4,5-trisubstituted isoxazole **3a** and 55% of a compound that we identified as furoxan **4** (a 1,2,5-oxadiazole 2-oxide) which formed through homo-coupling of substrate **1a** (Table 1, entry 3). Na_2_CO_3_ in 98% water, 2% methanol produced 52% of the isoxazole **3a** and 33% of compound **4** after 3 hours (Table 1, entry 4). Triethylamine (TEA) in 98% water, 2% methanol afforded 54% of **3a** and 22% of **4** after 2 hours (Table 1, entry 5). However, TEA in 5% water, 95% methanol improved the yield of **3a** to 68% as well as the selectivity of **3a** (3.6 times) over **4** (Table 1, entry 6). Similar yields and selectivities for **3a** were obtained when TEA was used in either dichloromethane (50% yield; 1.8 times selectivity) or isopropanol (57% yield; 1.9 times selectivity) as solvents (Table 1, entries 7 and 8). In contrast, *N*,*N*-diisopropylethylamine (DIPEA) in chloroform neither improved the yield (43%) nor the selectivity (0.8 times) of **3a** (Table 1, entry 9). In dichloromethane, DIPEA furnished an even lower yield (17%) and selectivity (0.2 times) of **3a** (Table 1, entry 10). Interestingly, in 5% water, 95% methanol, DIPEA gave product **3a** in 89% yield in 1 hour, while in 95% water, 5% methanol, DIPEA gave **3a** in 98% yield in 1 hour, which is the highest yield of our screening in the shortest reaction time (Table 1, entries 11 and 12). The reaction furnished a similar yield (95%) with a reduced amount of DIPEA and reactant concentration (2 equivalents of DIPEA and 1 mM of reactants **1a** and **2a**). However, these conditions required a longer reaction time (5 hours) to complete (Table 1, entry 13). Additionally, we observed a high selectivity towards compound **4**, when we reduced the amount of DIPEA to 10 mol % in 95% water, 5% methanol (Table 1, entry 14) or when the reaction was performed without a base in 5% water, 95% methanol (Table 1, entry 15); in these cases, compound **4** was obtained in excellent yields (70% yield and 95% yield, respectively). Compound **4** was also formed almost exclusively in 76% yield when the reaction was run in phosphate buffer (pH 7.4) in the presence of 5% methanol with 10 mol % of DIPEA (Table 1, entry 16). Notably, no other regioisomers of isoxazole **3a** were overserved in any of these reaction conditions.

**Figure 3:**
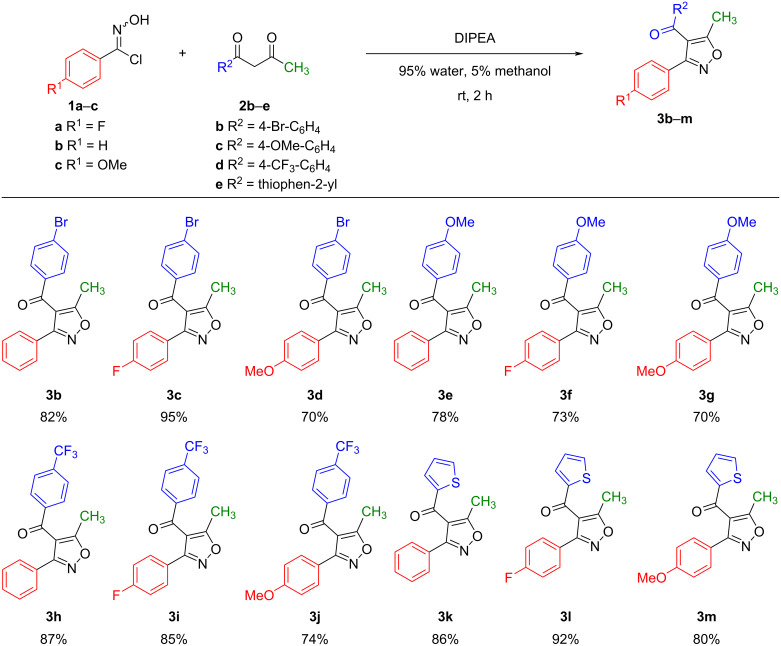
Reactions between various arylhydroximoyl chlorides and 1,3-diketones. The reactions were performed with 0.5 mmol of **1**, 0.5 mmol of **2**, and 3 equivalents of DIPEA in 15 mL of 95% water, 5% methanol at room temperature for 2 hours. The yields were calculated after the isolation and purification of products.

With the optimized reaction conditions for the formation of 3,4,5-trisubstituted isoxazoles **3a** (DIPEA, 95% water, 5% methanol, room temperature, Table 1, entry 12) at hands, we explored the scope of substrates for both the oximes and the β-diketones. First, we carried out the reactions between phenyl hydroximoyl chlorides **1a–c** and 1,3-diketones **2b–e** (Figure 3). To ensure completion, all reactions were run for 2 hours instead of 1 hour, regardless of substrates having either electron-withdrawing or electron-donating substituents on the benzene rings. The results showed smooth and complete reactions that produced the 3,4,5-trisubstituted isoxazoles **3b–m** in good to excellent yields. The overall trend was that electron-donating substituents on the phenyl hydroximoyl chlorides or the phenyl 1,3-diketones produced comparatively lower yields than electron-withdrawing substituents. For example, the reactions afforded a yield from 70% to 80% when one of the reactants contained a *p*-methoxyphenyl group (compounds **3d–g**,**j**, and **m**), whereas the reactions afforded a yield from 82% to 95% when one of the reactants contained a Br, F, or CF_3_ group in the *para*-position or contained a thiophene ring (compounds **3b**,**c**,**h**,**i**,**k**, and **l**).

**Figure 4:**
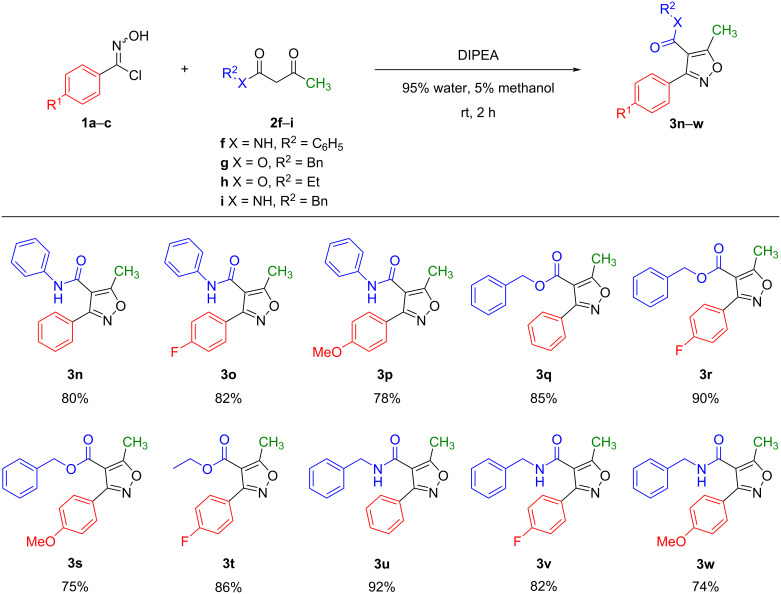
Reactions between various phenyl hydroximoyl chlorides and β-ketoesters or β-ketoamides. The reactions were performed with 0.5 mmol of **1**, 0.5 mmol of **2**, and 3 equivalents of DIPEA in 15 mL of 95% water, 5% methanol at room temperature for 2 hours. The yields were calculated after the isolation and purification of products.

We also explored the reactions between phenyl hydroximoyl chlorides **1a–c** and β-ketoesters **2g**,**h** or β-ketoamides **2f**,**i** under the optimized reaction conditions (DIPEA, 95% water, 5% methanol, room temperature, 2 hours). The results showed that phenyl, benzyl, and ethyl β-ketoesters or β-ketoamides reacted smoothly with the phenyl hydroximoyl chlorides and gave the corresponding 3,4,5-trisubstituted isoxazoles **3n**–**w** in good to excellent yields (Figure 4). Generally, the yields of the reactions with β-ketoesters and β-ketoamides were comparable with those obtained with 1,3-diketones.

**Figure 5:**
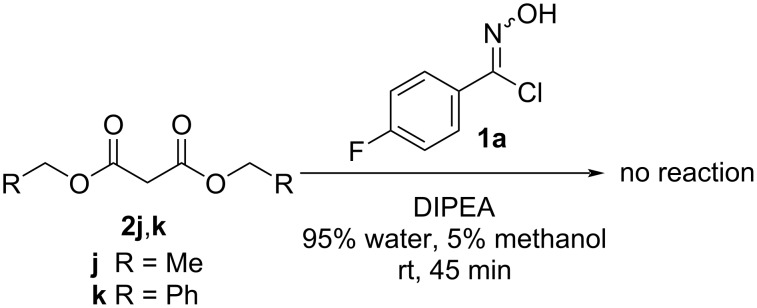
Reactions between 4-fluorophenyl hydroximoyl chloride (**1a**) and diethyl malonate (**2j**) or dibenzyl malonate (**2k**) did not proceed.

We also explored the reactions between 4-fluorophenyl hydroximoyl chloride (**1a**), which was the phenyl hydroximoyl chloride that gave the best yield, and diethyl malonate (**2j**) or dibenzyl malonate (**2k**) under the optimized reaction conditions. However, the reactions did not proceed (Figure 5). We suspected that the increased electron-donating effect on both sides of the 1,3-diketones in **2j** or **2k** made the methylene group less acidic, thus DIPEA was not able to deprotonate the methylene for the nucleophilic addition and successive cyclization to happen.

In addition, we further explored the scope of substrates with a trifluoromethyl group, a strong electron-withdrawing substituent, instead of methyl, an electron-donating group, in the 1,3-diketone starting materials (Table 2). The reaction between 4-fluorophenyl hydroximoyl chloride (**1a**) and 4,4,4-trifluoro-1-phenyl-1,3-butanedione (**2l**) under the optimized reaction conditions (Table 2, entry 1) gave a complex mixture of products with only a trace amount of the expected trifluoromethyl-substituted isoxazole **3x** being detected. Meanwhile, some of the starting material **2l** remained unreacted and we suspected the lower solubility of compound **2l** in water was responsible for the low yield of the reaction. Since trifluoromethyl-substituted isoxazoles are an important and prevalent scaffold in biomedical research and drug discovery programs, we decided to optimize the reaction conditions for the synthesis of the trifluoromethyl-substituted isoxazole **3x**. We varied the solvent mixtures and the bases, but kept the reaction temperature at room temperature and the reaction time at 2 hours, because these are the highlights of our synthetic method. The results are collected in Table 2. First, we varied the proportion of methanol in the solvent mixture with water to increase the solubility of 4,4,4-trifluoro-1-phenyl-1,3-butanedione (**2l**). As the percentage of methanol in the solvent mixture increased from 5% to 50%, 75%, and 95%, with DIPEA as the base, the yield of the trifluoromethyl-substituted isoxazole **3x** increased from trace amounts to 40% (Table 2, entries 1–4). With dichloromethane as the solvent and DIPEA as the base, the reaction gave **3x** in 40% yield (Table 2, entry 5). When isopropanol was used as the solvent and TEA was used as the base, the reaction produced **3x** in 40% yield (Table 2, entry 9). When benzene, dichloromethane, or a 5% water, 95% methanol mixture was used as the solvent and TEA was used as the base, the reaction gave a yield from 5% to 22% (Table 2, entries 6–8). Overall, the best results, i.e., 40% yield of the trifluoromethyl-substituted isoxazole **3x**, were obtained with DIPEA, 5% water, 95% methanol, room temperature, 2 hours, or with DIPEA, dichloromethane, room temperature, 2 hours, or with TEA, isopropanol, room temperature, 2 hours (Table 2, entries 4, 5, and 9).

**Table 2:** Optimization of the reaction conditions to synthesize the trifluoromethyl-substituted isoxazole **3x**.^a^

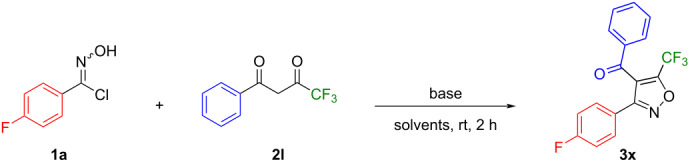			

Entry	Base	Solvents	Yield of **3x** (%)

1	DIPEA	95% water, 5% methanol	traces
2	DIPEA	50% water, 50% methanol	traces
3	DIPEA	25% water, 75% methanol	30
4	DIPEA	5% water, 95% methanol	40
5	DIPEA	dichloromethane	40
6	TEA	5% water, 95% methanol	22
7	TEA	dichloromethane	20
8	TEA	benzene	5
9	TEA	isopropanol	40

^a^The reactions were performed with 0.5 mmol of 4-fluorophenyl hydroximoyl chloride (**1a**), 0.5 mmol of 4,4,4-trifluoro-1-phenyl-1,3-butanedione (**2l**), and 3 equivalents of base in 15 mL of the indicated solvent at room temperature for 2 hours.

**Figure 6:**
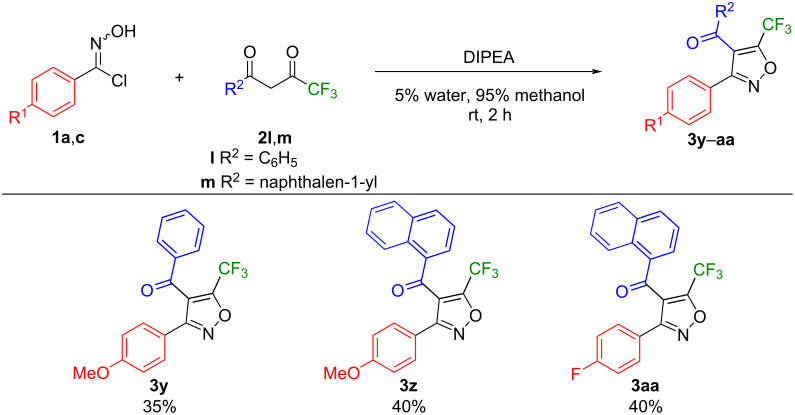
Reactions between phenyl hydroximoyl chlorides **1a**,**c** and 4,4,4-trifluoro-1-phenyl- (**2l**) and 4,4,4-trifluoro-1-naphthylbutane-1,3-dione (**2m**) to synthesize trifluoromethyl-substituted isoxazoles **3y**–**aa**.

We then applied the reaction conditions DIPEA, 5% water, 95% methanol, room temperature, 2 hours (Table 2, entry 4) to the reactions between phenyl hydroximoyl chlorides **1a**,**c** and 4,4,4-trifluoro-1-phenyl- (**2l**) and 4,4,4-trifluoro-1-naphthylbutane-1,3-dione (**2m**) to synthesize the trifluoromethyl-substituted isoxazoles **3y**–**aa** (Figure 6). The reactions proceeded smoothly and produced the expected trifluoromethyl-substituted isoxazole products. Similar to the yield of compound **3x**, the yields of **3y–aa** were between 35–40%. Overall, the reactions to synthesize trifluoromethyl-substituted isoxazoles produced lower yields than those of methyl-substituted isoxazoles.

**Figure 7:**
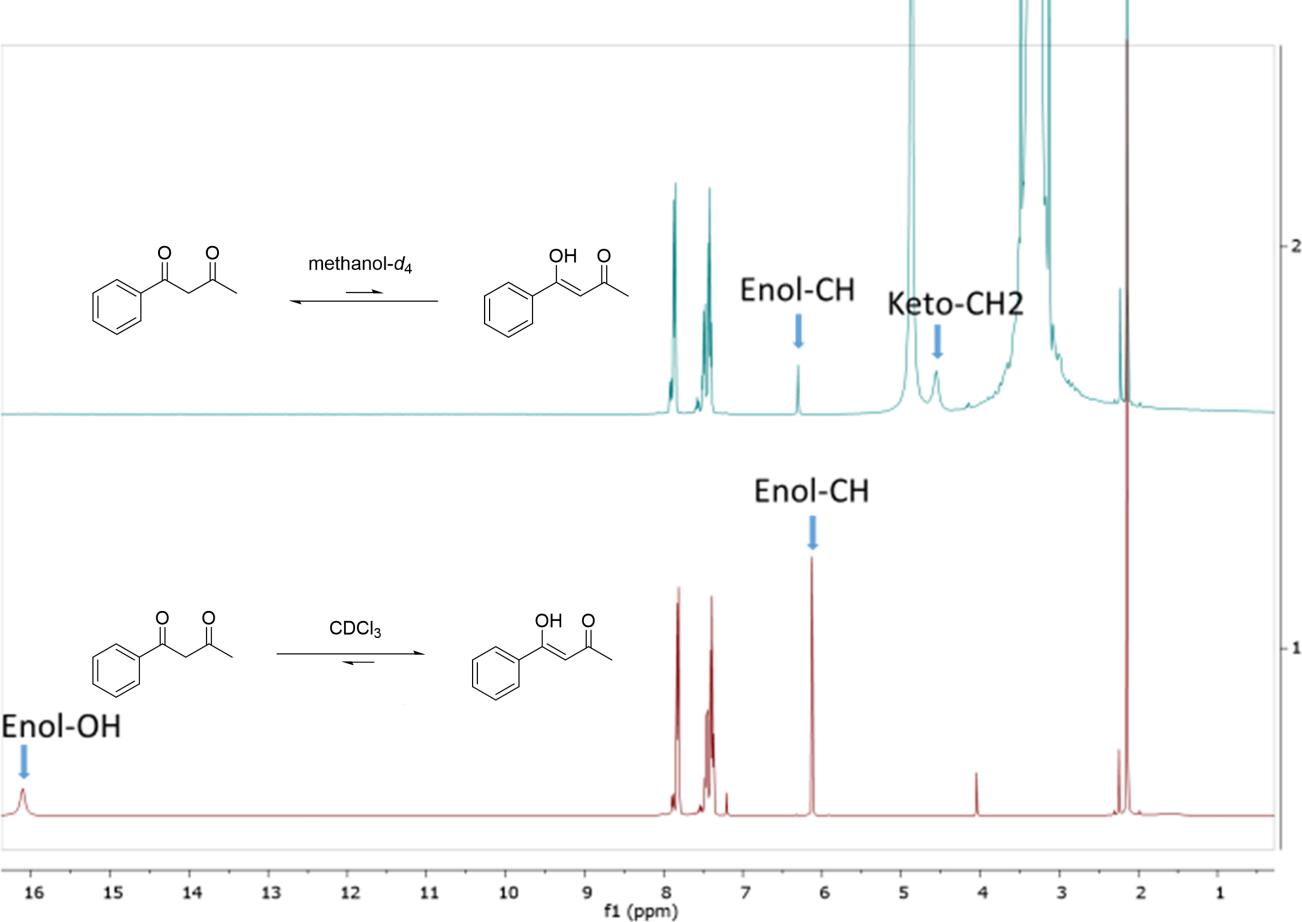
^1^H NMR spectra of 1-phenyl-1,3-butanedione (**2a**) in methanol-*d*_4_ (top) and in CDCl_3_ (bottom).

Our observed results with 1,3-diketones with substituents of different electronic nature and the variety of the polarities of the solvents can be explained by the enolization of the 1,3-diketones as previously was described in the literature [31–32]. Strongly electron-withdrawing substituents present in the 1,3-diketones would increase the degree of enolization of the 1,3-diketones [30,33], and the polarity of the solvent would have only a little effect on the keto–enol tautomerization of such 1,3-diketones [30]. During our studies, we also observed the effect of solvent polarity on the keto–enol equilibrium of the 1,3-diketones, which influences the results of the [3 + 2] cycloaddition reactions under mild basic conditions, via nuclear magnetic resonance (NMR) spectroscopy. The ^1^H NMR spectra of 1-phenyl-1,3-butanedione (**2a**) in CDCl_3_ and in methanol-*d*_4_ indicated that the enol tautomer of the 1,3-diketone was predominant in CDCl_3_, while the keto tautomer was predominant in methanol-*d*_4_, which is a more polar solvent than CDCl_3_ (Figure 7). Our observations thus reinforce Meyer’s rule [34–35], which states that the keto tautomer is favored as the solvent polarity increases. This also corroborates our observations and explains why under mild basic conditions, the 3,4,5-trisubstituted isoxazole **3a** was formed exclusively in polar solvents like water or methanol (Table 1, entries 11–13), whereas the furoxan **4** was formed predominantly in nonpolar solvents such as chloroform or dichloromethane (Table 1, entries 9 and 10).

**Figure 8:**
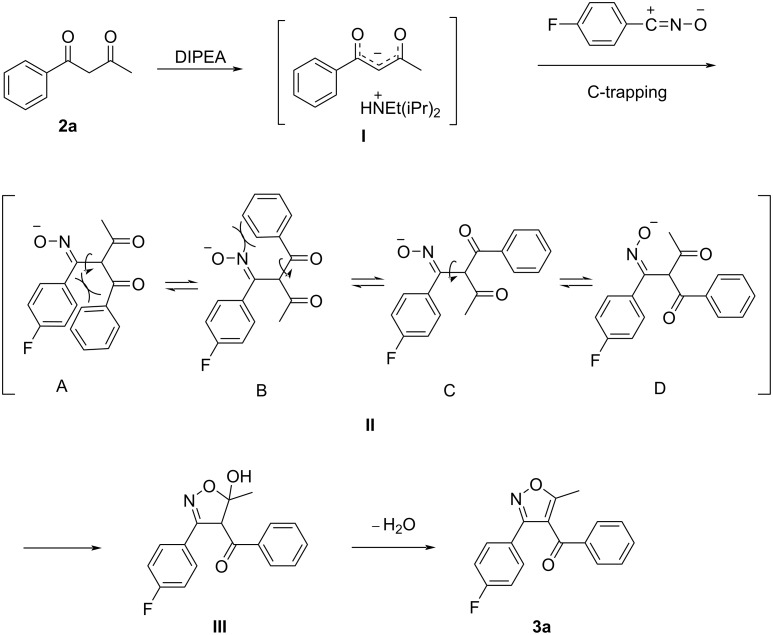
A plausible mechanism for the formation of the 3,4,5-trisubstituted isoxazoles **3** in the presence of DIPEA in polar solvents like water or methanol.

A plausible mechanism for the formation of the 3,4,5-trisubstituted isoxazoles **3a** (the only regioisomer that was formed) is shown in Figure 8. In a polar solvent like water or methanol, the 1,3-diketone is deprotonated by the base DIPEA to form the carbanion **I** that is solvated by the polar solvent. The carbanion **I** then adds to the carbon (carbocation) of the nitrile oxide, to give the intermediate **II**. Due to steric effects, the intermediate **II** undergoes bond rotation to the lowest-energy conformation **II-D** in which the methyl group is at a close distance to the nitrile oxide. Then, cyclization followed by the elimination of water (formation of the aromatic ring is the driving force), produces the 3,4,5-trisubstituted isoxazole **3a** (Figure 8). Of note, **II-A–II-D** are conformers, not resonance structures, and the isoxazole **3a** is likely the thermodynamical product. The solvent polarity also affects the keto–enol equilibrium of the intermediate **II-D**. In polar solvents, the keto tautomer is predominant as an electrophilic group for the intramolecular cyclization, while in nonpolar solvents, the enol tautomer could not accept a nucleophilic attack for a further cycloaddition. In addition, the conjugation between the new C=N bond and the enol double bond promotes the enol tautomer formation. This may also explain why the trifluoromethylated 1,3-diketones produced the trifluoromethyl-substituted isoxazoles with lower yields than the methyl-substituted isoxazoles. Significant enolization of the corresponding trifluoromethyl intermediate **II-D** could not transfer into the target sufficiently. Furthermore, when the amount of DIPEA was reduced to 0–10 mol % (Table 1, entries 14–16), or when the reaction was carried out in a nonpolar solvent like chloroform or dichloromethane, the amount of carbanion **I** in the reaction medium is much smaller. Therefore, the formation of the homo-coupled product (furoxan **4**) outcompeted the [3 + 2]-cycloaddition.

Not to be overlooked is our discovery of the optimized reaction conditions for the formation of furoxan **4** with 95% yield (5% water, 95% methanol, room temperature, 2 hours; see Table 1, entry 15), which adds another method for the synthesis of furoxans to the current literature. The synthesis of furoxans or 1,2,5-oxadiazole-2-oxides was first reported by Kekulé in 1857 [36]. Since then, these nitric oxide donors have shown important biological activities due to their unique electronic and coordination ability [37–38], such as antitumor [39] and antiparasitic [40–41]. Previously, furoxans were synthesized via dimerization of hydroximoyl chlorides in the presence of a base, such as trimethylamine in ether [42], or Na_2_CO_3_ in DME [43]. Our results showed that when 5% water, 95% methanol was used as a solvent mixture, the dimerization of hydroximoyl chlorides happened fast at room temperature without the need of a base. The obtained furoxan **4** was found to be a stable product; when stirring it in a 5% water, 95% methanol mixture in the presence of DIPEA overnight, no degradation was observed.

## Conclusion

**Figure 9:**
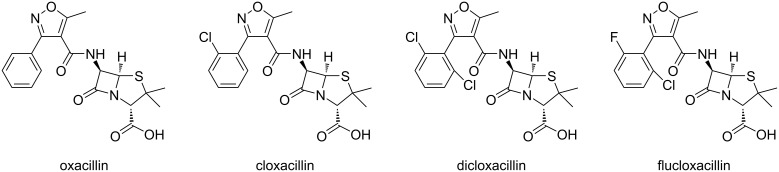
Structures of β-lactamase-resistant antibiotics oxacillin, cloxacillin, dicloxacillin, and flucloxacillin.

We have developed a method for the synthesis of 3,4,5-trisubstituted isoxazoles in aqueous medium under mild basic conditions at room temperature. The reaction proceeds via a [3 + 2]-cycloaddition of nitrile oxides and 1,3-diketones, β-ketoesters, or β-ketoamides. When DIPEA is used as the base and 95% water, 5% methanol is used as the solvent mixture, the reaction is complete within 1–2 hours at room temperature. Our method provides a fast and environmentally friendly access to 3,4,5-trisubstituted isoxazoles, an important class of structures found in numerous bioactive natural products and pharmaceuticals. Producing good to excellent yields in short reaction time in aqueous media, our method has the potential for significant applications in biological systems (e.g., click reactions, bioconjugation, and bio-orthogonal chemistry). Our method could also find potential applications in the production of important 3,4,5-trisubstituted isoxazoles, such as the precursors of many β-lactamase-resistant antibiotics like oxacillin, cloxacillin, dicloxacillin, and flucloxacillin (Figure 9), which share a similar 3,4,5-trisubstituted isoxazole structure to the synthesized compounds in this report. In addition, we optimized the reaction conditions to produce trifluoromethyl-substituted isoxazoles, a prevalent scaffold in biomedical research and drug discovery programs. We also proposed a plausible mechanism for the selectivity of the [3 + 2]-cycloaddition reaction to produce 3,4,5-trisubstituted isoxazoles. Not to be overlooked is our optimized reaction conditions for the dimerization of hydroximoyl chlorides to form furoxans or 1,2,5-oxadiazole 2-oxides, a class of structures with important biological activities due to their unique electronic and coordination ability.

## Experimental

### General information

All chemicals and solvents were obtained from Sigma-Aldrich or Fisher Scientific and were used as received unless specified. ^1^H NMR and ^13^C NMR spectra were recorded on a Bruker-400 and or a Bruker-500 spectrometer using CDCl_3,_ methanol-*d*_4_, or DMSO-*d*_6_ as the solvent. Chemical shifts (δ) were recorded in parts per million and referenced to CDCl_3_ (7.24 ppm for ^1^H NMR and 77.23 ppm for ^13^C NMR), methanol-*d*_4_ (3.31 ppm for ^1^H NMR and 49.15 ppm for ^13^C NMR), or DMSO-*d*_6_ (2.50 ppm for ^1^H NMR and 39.52 ppm for ^13^C NMR). ^19^F NMR spectra were recorded on a Bruker-400 spectrometer. Coupling constants (*J*) are given in Hz. The following abbreviations were used to designate the multiplicities: s = singlet, d = doublet, t = triplet, q = quartet, quint = quintet, m = multiplet, br = broad. Melting points were measured using an OptiMelt automated melting point system. Exact high-resolution mass determinations were analyzed on a JEOL AccuToF 4G LCplus atmospheric pressure ionization time-of-flight mass spectrometer (Jeol, Tokyo, Japan) fitted with direct analysis in real-time (DART) ion source (IonSense DART controller, Saugus, MA, USA). The DART ion source was operated with helium gas (approximately 4.0 L/min flow rate), the gas heater (350 °C), and the source grid (350 V). The data acquisition range was from *m/z* 50 to 1000. Polyethylene glycol (PEG 600) was used for the exact mass calibration.

### General synthetic procedure for phenyl hydroximoyl chlorides

The phenyl hydroximoyl chlorides (Figure S1 in Supporting Information File 1) were synthesized by following literature procedures [44–45]. To a solution of the corresponding benzaldehyde (1 mmol) in a 1:1 EtOH/H_2_O mixture (1 mL) was added sodium acetate (1.1 mmol) and hydroxylamine hydrochloride (1.1 mmol). The reaction mixture was stirred for 4 h at rt. After the reaction was complete, as indicated by thin-layer chromatography (TLC), the mixture was extracted with ethyl acetate. The organic phase was dried with Na_2_SO_4_ and concentrated under reduced pressure. Purification via column chromatography (ethyl acetate/hexanes) resulted in the aldoxime product.

To a solution of the aldoxime (1 mmol) in DMF was added *N*-chlorosuccinimide and the mixture stirred for 18 h at rt. After the reaction was complete, as indicated by TLC, the reaction mixture was poured into water and extracted with ethyl acetate. The organic phase was washed with brine and dried with Na_2_SO_4_. The solvent was then evaporated under reduced pressure to produce the desired phenyl hydroximoyl chloride, which was used immediately for the [3 + 2]-cycloaddition reaction without further purification.

### General synthetic procedure for 1,3-diketones

The 1,3-diketones (Figure S2 in Supporting Information File 1) were synthesized by following a literature procedure [46]. To a suspension of NaH (1.60 g of dispersion in oil, 40 mmol) in ethyl acetate (20 mL) at 0 °C was added slowly a solution of the corresponding ketone (10 mmol) in ethyl acetate (20 mL). The reaction mixture was stirred at rt for 12 h. After the reaction was complete, as indicated by TLC, the mixture was carefully treated with 10% aqueous NH_4_Cl (30 mL) and the pH adjusted to 5 with a solution of hydrochloric acid (3 M). The aqueous phase was then separated and extracted with ethyl acetate. The organic phase was dried with Na_2_SO_4_ and concentrated under reduced pressure. Purification via column chromatography (20:1 hexanes/ethyl acetate) resulted in the desired 1,3-diketone.

### General synthetic procedure for the [3 + 2]-cycloaddition reaction

To a solution of the 1,3-diketone, β-ketoester, or β-ketoamide (0.5 mmol, 1 equiv) in methanol was added water, phenyl hydroximoyl chloride (1 equiv), and DIPEA (3 equiv) at room temperature (total volume of methanol and water = 15 mL; 95% water, 5% methanol). The reaction mixture was stirred for 1–2 h until all of the starting materials were consumed (TLC, 10% ethyl acetate in hexanes). After the reaction was complete, the product was extracted with ethyl acetate, dried with Na_2_SO_4_, concentrated under reduced pressure, and purified via column chromatography (ethyl acetate/hexanes).

## Supporting Information

File 1 Synthetic schemes for phenyl hydroximoyl chlorides and 1,3-diketones, characterization data, and copies of ^1^H, ^13^C, and ^19^F NMR spectra.
