# Supplementary material for: Synthesis of 3,4,5-trisubstituted isoxazoles in water via a [3 + 2]-cycloaddition of nitrile oxides and 1,3-diketones, β-ketoesters, or β-ketoamides
Source: Beilstein J Org Chem. 2022 Apr 22;18:446–58. doi: 10.3762/bjoc.18.47 (PMC9039522; doi:10.3762/bjoc.18.47)
Supplement: File 1 — Synthetic schemes for phenyl hydroximoyl chlorides and 1,3-diketones, characterization data, and copies of 1H, 13C, and 19F NMR spectra. [file Beilstein_J_Org_Chem-18-446-s001.pdf]

BEILSTEIN JOURNAL OF ORGANIC CHEMISTRY

Supporting Information

for

Synthesis of 3,4,5-trisubstituted isoxazoles in water via a [3 + 2]-cycloaddition of nitrile oxides and 1,3-diketones, β-ketoesters, or β-ketoamides

Md Imran Hossain, Md Imdadul H. Khan, Seong Jong Kim and Hoang V. Le

Beilstein J. Org. Chem. **2022,** *18,* 446–458. doi:10.3762/bjoc.18.47

Synthetic schemes for phenyl hydroximoyl chlorides and 1,3-diketones, characterization data, and copies of ^{1}H, ^{13}C, and ^{19}F NMR spectra

License and Terms: This is a supporting information file under the terms of the Creative Commons Attribution License (https://creativecommons.org/licenses/by/4.0). Please note that the reuse, redistribution and reproduction in particular requires that the author(s) and source are credited and that individual graphics may be subject to special legal provisions.
The license is subject to the *Beilstein Journal of Organic Chemistry* terms and conditions: (https://www.beilstein-journals.org/bjoc/terms)

Table of contents

	Page
**Figure S1:** Synthesis of phenyl hydroximoyl chlorides **1a–c**	S3
**Figure S2:** Synthesis of 1,3-diketones **2b–e**	S3
Characterization of compounds **3a–aa** and **4**	S3
Reference	S10
**Figure S3:** ^{1}H and ^{13}C NMR spectra of compound **3a**	S11
**Figure S4:** ^{19}F NMR spectrum of compound **3a**	S12
**Figure S5:** ^{1}H and ^{13}C NMR spectra of compound **4**	S13
**Figure S6:** ^{19}F and HMBC NMR spectra of compound **4**	S14
**Figure S7:** ^{1}H and ^{13}C NMR spectra of compound **3b**	S15
**Figure S8:** ^{1}H and ^{13}C NMR spectra of compound **3c**	S16
**Figure S9:** ^{19}F spectrum of compound **3c**	S17
**Figure S10:** ^{1}H and ^{13}C NMR spectra of compound **3d**	S18
**Figure S11:** ^{1}H and ^{13}C NMR spectra of compound **3e**	S19
**Figure S12:** ^{1}H and ^{13}C NMR spectra of compound **3f**	S20
**Figure S13:** ^{19}F NMR spectrum of compound **3f**	S21
**Figure S14:** ^{1}H and ^{13}C NMR spectra of compound **3g**	S22
**Figure S15:** ^{1}H and ^{13}C NMR spectra of compound **3h**	S23
**Figure S16:** ^{19}F NMR spectrum of compound **3h**	S24
**Figure S17:** ^{1}H and ^{13}C NMR spectra of compound **3i**	S25
**Figure S18:** ^{19}F NMR spectrum of compound **3i**	S26
**Figure S19:** ^{1}H and ^{13}C NMR spectra of compound **3j**	S27
**Figure S20:** ^{19}F NMR spectrum of compound **3j**	S28
**Figure S21:** ^{1}H and ^{13}C NMR spectra of compound **3k**	S29
**Figure S22:** ^{1}H and ^{13}C NMR spectra of compound **3l**	S30
**Figure S23:** ^{19}F NMR spectrum of compound **3l**	S31
**Figure S24:** ^{1}H and ^{13}C NMR spectra of compound **3m**	S32
**Figure S25:** ^{1}H and ^{13}C NMR spectra of compound **3n**	S33
**Figure S26:** ^{1}H and ^{13}C NMR spectra of compound **3o**	S34
**Figure S27:** ^{19}F NMR spectrum of compound **3o**	S35
**Figure S28:** ^{1}H and ^{13}C NMR spectra of compound **3p**	S36
**Figure S29:** ^{1}H and ^{13}C NMR spectra of compound **3q**	S37
**Figure S30:** ^{1}H and ^{13}C NMR spectra of compound **3r**	S38
**Figure S31:** ^{19}F NMR spectrum of compound **3r**	S39
**Figure S32:** ^{1}H and ^{13}C NMR spectra of compound **3s**	S40
**Figure S33:** ^{1}H and ^{13}C NMR spectra of compound **3t**	S41
**Figure S34:** ^{19}F NMR spectrum of compound **3t**	S42
**Figure S35:** ^{1}H and ^{13}C NMR spectra of compound **3u**	S43

**Figure S36:** ^{1}H and ^{13}C NMR spectra of compound **3v**	S44
**Figure S37:** ^{19}F NMR spectrum of compound **3v**	S45
**Figure S38:** ^{1}H and ^{13}C NMR spectra of compound **3w**	S46
**Figure S39:** ^{1}H and ^{13}C NMR spectra of compound **3x**	S47
**Figure S40:** ^{19}F NMR spectrum of compound **3x**	S48
**Figure S41:** ^{1}H and ^{13}C NMR spectra of compound **3y**	S49
**Figure S42:** ^{19}F NMR spectrum of compound **3y**	S50
**Figure S43:** ^{1}H and ^{13}C NMR spectra of compound **3z**	S51
**Figure S44:** ^{19}F NMR spectrum of compound **3z**	S52
**Figure S45:** ^{1}H and ^{13}C NMR spectra of compound **3aa**	S53
**Figure S46:** ^{19}F NMR spectrum of compound **3aa**	S54

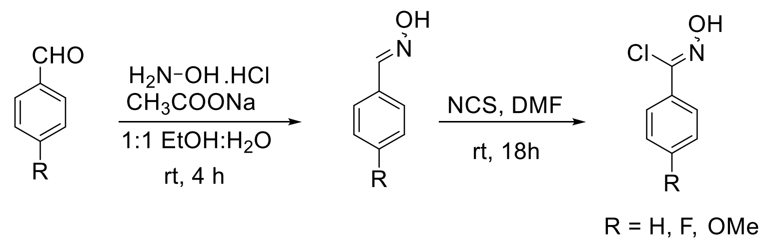

Figure S1: Synthesis of phenyl hydroximoyl chlorides **1a**–**c**.

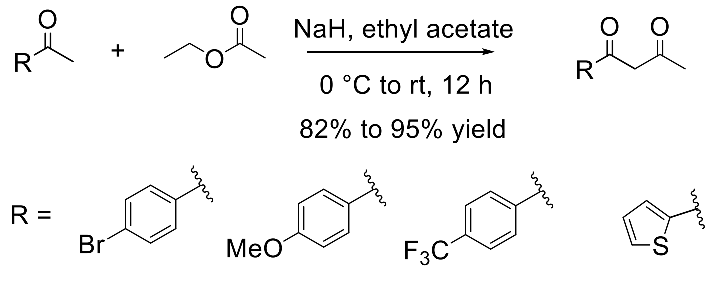

Figure S2: Synthesis of 1,3-diketones **2b**–**e**.

Characterization of compounds 3a–aa and 4:

(3-(4-Fluorophenyl)-5-methylisoxazol-4-yl)(phenyl)methanone (3a)

Yield: 98%. Yellow sticky gel. ^{1}H NMR (500 MHz, $CDCl_3$) δ 7.69 – 7.62 (m, 2H), 7.49 – 7.39 (m, 3H), 7.32 (tt, *J* = 7.5, 1.4 Hz, 2H), 6.99 – 6.85 (m, 2H), 2.46 (s, 3H). ^{13}C NMR (126 MHz, $CDCl_3$) δ 189.96, 173.09, 163.57 (d, *J* = 250 Hz), 161.05, 137.33, 133.62, 130.61 (d, *J* = 7.5 Hz), 129.49, 128.61, 124.38 (d, *J* = 3.8 Hz), 115.73, 115.57 (d, *J* = 3.8 Hz), 12.87. ^{19}F NMR (471 MHz, $CDCl_3$) δ -110.63. HRMS *m/z* calcd. for $C_{17}H_{13}FNO_2$ $[M+H]^+$ 282.0930, found 282.0930.

3,4-Bis(4-fluorophenyl)-1,2,5-oxadiazole 2-oxide (4)

General synthetic procedure for the [3 + 2]-cycloaddition reaction was followed but with 5% water, 95% methanol as the solvent mixture and no DIPEA. Yield: 95%. Clear sticky gel. ^{1}H NMR (400 MHz, DMSO-d_6) δ 7.60–7.56 (m, 4H), 7.42–7.37 (m, 4H). ^{13}C NMR (101 MHz, DMSO-d_6) δ 164.54 (d, *J* = 57 Hz), 162.06 (d, *J* = 57 Hz), 155.90, 131.65 (d, *J* = 9.1 Hz), 130.86 (d, *J* = 9.1

Hz), 122.73 (d, J = 3.2 Hz), 119.04 (d, J = 3.2 Hz), 116.44 (d, J = 7.7 Hz), 116.22 (d, J = 7.7 Hz), 114.18. ^{19}F NMR (377 MHz, DMSO- d_6) δ -108.74 (ttd, J = 8.9, 5.4, 1.8 Hz, 1F), -108.82 (ttd, J = 8.9, 5.4, 1.4 Hz, 1F). HRMS m/z calcd. for $C_{14}H_9N_2O_2F_2$ $[M+H]^+$ 275.0632, found 275.0637.

(4-Bromophenyl)(5-methyl-3-phenylisoxazol-4-yl)methanone (3b)

Yield: 82%. White crystal. Mp 105–107 °C. ^{1}H NMR (500 MHz, $CDCl_3$) δ 7.55 (d, J = 8.4 Hz, 2H), 7.49 – 7.38 (m, 4H), 7.35 (t, J = 7.3 Hz, 1H), 7.29 (t, J = 7.4 Hz, 2H), 2.55 (s, 3H); ^{13}C NMR (126 MHz, $CDCl_3$) δ 189.01, 173.41, 161.83, 136.11, 131.86, 131.01, 130.01, 128.74, 128.66, 128.60, 128.04, 115.40, 12.91. HRMS m/z calcd. for $C_{17}H_{13}BrNO_2$ $[M+H]^+$ 342.0130, found 342.0134.

(4-Bromophenyl)(3-(4-fluorophenyl)-5-methylisoxazol-4-yl)methanone (3c)

Yield: 95%. White solid. Mp 84–86 °C. ^{1}H NMR (500 MHz, $CDCl_3$) δ 7.54 (d, J = 8.6 Hz, 2H), 7.48 (d, J = 8.3 Hz, 2H), 7.44 (t, J = 8.6 Hz, 2H), 6.98 (t, J = 8.6 Hz, 2H), 2.50 (s, 3H); ^{13}C NMR (126 MHz, $CDCl_3$) δ 188.90, 173.39, 163.77 (d, J = 250 Hz), 160.95, 136.08, 132.04, 131.01, 130.66 (d, J = 8.8 Hz), 129.03, 124.23 (d, J = 3.8 Hz), 115.92(d, J = 21 Hz), 115.33, 13.00. ^{19}F NMR (101 MHz, $CDCl_3$) δ -110.18. HRMS m/z calcd. for $C_{17}H_{12}BrFNO_2$ $[M+H]^+$ 360.0035, found 360.0049.

(4-Bromophenyl)(3-(4-methoxyphenyl)-5-methylisoxazol-4-yl)methanone (3d)

Yield: 70%. White solid. Mp 91–93 °C. ^{1}H NMR (400 MHz, $CDCl_3$) δ 7.54 (d, J = 8.5 Hz, 2H), 7.45 (d, J = 8.6 Hz, 2H), 7.36 (d, J = 8.8 Hz, 2H), 6.78 (d, J = 8.8 Hz, 2H), 3.76 (s, 3H), 2.49 (s, 3H); ^{13}C NMR (101 MHz, $CDCl_3$) δ 189.23, 173.12, 160.96, 136.18, 131.91, 131.07, 130.05, 128.77, 120.31, 115.11, 114.44, 114.15, 55.41, 12.89. HRMS m/z calcd. for $C_{18}H_{15}BrNO_3$ $[M+H]^+$ 372.0235, found 372.0245.

(4-Methoxyphenyl)(5-methyl-3-phenylisoxazol-4-yl)methanone (3e)

Yield: 78%. White solid. Mp 88–90 °C. ^{1}H NMR (400 MHz, $CDCl_3$) δ 7.73 (d, J = 8.9 Hz, 2H), 7.56 – 7.48 (m, 2H), 7.38 – 7.26 (m, 3H), 6.83 (d, J = 8.9 Hz, 2H), 3.82 (s, 3H), 2.50 (s, 3H); ^{13}C NMR (101 MHz, $CDCl_3$) δ 188.62, 171.89, 164.06, 161.78, 132.12, 130.26, 129.82, 128.60,

128.54, 128.36, 115.80, 113.88, 55.59, 12.69. HRMS *m/z* calcd. for $C_{18}H_{16}NO_3$ $[M+H]^+$ 294.1130, found 294.1143.

(3-(4-Fluorophenyl)-5-methylisoxazol-4-yl)(4-methoxyphenyl)methanone (3f)

Yield: 73%. White crystal. Mp 115–117 °C. ^{1}H NMR (400 MHz, $CDCl_3$) δ 7.69 (d, *J* = 8.9 Hz, 2H), 7.49 (dd, *J* = 8.8, 5.3 Hz, 2H), 6.96 (t, *J* = 8.7 Hz, 2H), 6.81 (d, *J* = 8.9 Hz, 2H), 3.80 (s, 3H), 2.44 (s, 3H); ^{13}C NMR (101 MHz, $CDCl_3$) δ 188.43, 171.97, 164.16, 163.60 (d, *J* = 250 Hz), 160.84, 132.07, 130.50 (d, *J* = 9.1 Hz), 130.14, 124.52 (d, *J* = 3.0 Hz), 115.82, 115.64 (d, *J* = 5.0 Hz), 113.94, 55.57, 12.69. ^{19}F NMR (101 MHz, $CDCl_3$) δ -110.72. HRMS *m/z* calcd. for $C_{18}H_{15}FNO_3$ $[M+H]^+$ 312.1036, found 312.1046.

(4-Methoxyphenyl)(3-(4-methoxyphenyl)-5-methylisoxazol-4-yl)methanone (3g)

Yield: 70%. White solid. Mp 84–86 °C. ^{1}H NMR (500 MHz, $CDCl_3$) δ 7.75 – 7.69 (m, 2H), 7.47 – 7.41 (m, 2H), 6.85 – 6.77 (m, 4H), 3.82 (s, 3H), 3.76 (s, 3H), 2.44 (s, 3H); ^{13}C NMR (126 MHz, $CDCl_3$) δ 188.89, 171.61, 164.10, 161.33, 160.82, 132.19, 130.33, 129.93, 120.70, 115.65, 114.10, 113.94, 55.63, 55.36, 12.70. HRMS *m/z* calcd. for $C_{19}H_{18}NO_4$ $[M+H]^+$ 324.1236, found 324.1246.

(5-Methyl-3-phenylisoxazol-4-yl)(4-(trifluoromethyl)phenyl)methanone (3h)

Yield: 87%. White solid. Mp 78–80 °C. ^{1}H NMR (400 MHz, $CDCl_3$) δ 7.73 (d, *J* = 7.8 Hz, 2H), 7.60 – 7.47 (m, 2H), 7.37 (dt, *J* = 6.9, 1.5 Hz, 2H), 7.34 – 7.19 (m, 3H), 2.58 (s, 3H). ^{13}C NMR (101 MHz, $CDCl_3$) δ 189.08, 174.32, 161.96, 140.29, 134.52 (d, *J* = 33 Hz), 130.03, 129.75, 128.71 (d, *J* = 12 Hz), 127.99, 125.48 (q, *J* = 3.7 Hz), 124.60, 122.14, 115.43, 13.06. ^{19}F NMR (377 MHz, $CDCl_3$) δ -63.29. HRMS *m/z* calcd. for $C_{18}H_{13}F_3NO_2$ $[M+H]^+$ 332.0898 , found 332.0888.

(3-(4-Fluorophenyl)-5-methylisoxazol-4-yl)(4-(trifluoromethyl)phenyl)methanone (3i)

Yield: 85%. White solid. Mp 116–118 °C. ^{1}H NMR (400 MHz, $CDCl_3$) δ 7.80 – 7.72 (m, 2H), 7.58 (d, *J* = 8.1 Hz, 2H), 7.46 – 7.34 (m, 2H), 7.00 – 6.89 (m, 10H), 2.53 (s, 3H). ^{13}C NMR (101 MHz, $CDCl_3$) δ 188.93, 174.22, 163.79 (d, *J* = 200 Hz), 161.07, 140.24, 134.79 (q, *J* = 26 Hz), 130.75 (d, *J* = 8.5 Hz), 129.78, 125.67 (q, *J* = 3.7 Hz), 124.12 (d, *J* = 3.4 Hz), 122.37, 115.90 (d, *J* = 17 Hz), 115.34, 13.14. ^{19}F NMR (377 MHz, $CDCl_3$) δ -63.25, -110.12. HRMS *m/z* calcd. for $C_{18}H_{12}F_4NO_2$ $[M+H]^+$ 350.0804, found 350.0798.

(3-(4-Methoxyphenyl)-5-methylisoxazol-4-yl)(4-(trifluoromethyl)phenyl)methanone (3j)

Yield: 74%. Yellowish white solid. Mp 69–71 °C. ^{1}H NMR (400 MHz, $CDCl_3$) δ 7.86 – 7.71 (m, 2H), 7.57 (d, *J* = 8.2 Hz, 2H), 7.32 (d, *J* = 8.7 Hz, 2H), 6.77 (d, *J* = 8.8 Hz, 2H), 3.76 (s, 3H), 2.56 (s, 3H); ^{13}C NMR (101 MHz, $CDCl_3$) δ 189.25, 174.06, 161.50, 161.00, 140.31, 134.47 (q, *J* = 33 Hz), 129.98 (d, *J* = 35 Hz), 125.50 (d, *J* = 4.0 Hz), 124.74, 121.97, 120.15, 115.27, 114.11, 55.36, 13.05; ^{19}F NMR (377 MHz, $CDCl_3$) δ -63.21. HRMS *m/z* calcd. for $C_{19}H_{15}F_3NO_3$ $[M+H]^+$ 362.1004, found 362.1005.

(5-Methyl-3-phenylisoxazol-4-yl)(thiophen-2-yl)methanone (3k)

Yield: 86%. White solid. Mp 103–105 °C. ^{1}H NMR (400 MHz, $CDCl_3$) δ 7.63 (dd, *J* = 4.9, 1.2 Hz, 1H), 7.54 (dd, *J* = 7.9, 1.8 Hz, 2H), 7.40 – 7.27 (m, 4H), 6.94 (dd, *J* = 4.9, 3.8 Hz, 1H), 2.54 (s, 3H); ^{13}C NMR (101 MHz, $CDCl_3$) δ 181.75, 172.00, 161.30, 144.02, 135.41, 135.21, 130.00, 128.75, 128.52, 128.26, 115.84, 12.67. HRMS *m/z* calcd. for $C_{15}H_{12}NO_2S$ $[M+H]^+$ 270.0589, found 270.0583.

(3-(4-Fluorophenyl)-5-methylisoxazol-4-yl)(thiophen-2-yl)methanone (3l)

Yield: 92%. White solid. Mp 56–58 °C. ^{1}H NMR (500 MHz, $CDCl_3$) δ 7.66 (t, *J* = 3.5 Hz, 1H), 7.59 – 7.50 (m, 2H), 7.32 (d, *J* = 3.8 Hz, 1H), 7.07 – 6.94 (m, 3H), 2.52 (s, 3H); ^{13}C NMR (126 MHz, $CDCl_3$) δ 181.59, 172.03, 163.74 (d, *J* = 250 Hz), 160.39, 143.91, 135.66, 135.22, 130.51 (d, *J* = 8.8 Hz), 128.35, 124.41 (d, *J* = 3.7 Hz), 116.00, 115.78 (d, *J* = 12 Hz), 12.72; ^{19}F NMR (471 MHz, $CDCl_3$) δ -110.34. HRMS *m/z* calcd. for $C_{15}H_{11}FNO_2S$ $[M+H]^+$ 288.0495, found 288.0490.

(3-(4-Methoxyphenyl)-5-methylisoxazol-4-yl)(thiophen-2-yl)methanone (3m)

Yield: 80%. Sticky gel. ^{1}H NMR (500 MHz, $CDCl_3$) δ 7.65 (t, *J* = 3.5 Hz, 1H), 7.54 – 7.46 (m, 2H), 7.32 (d, *J* = 3.5 Hz, 1H), 6.96 (d, *J* = 4.0 Hz, 1H), 6.88 – 6.80 (m, 2H), 3.78 (s, 3H), 2.52 (s, 3H); ^{13}C NMR (126 MHz, $CDCl_3$) δ 182.04, 171.76, 160.96, 160.87, 144.12, 135.44, 135.29, 129.93, 128.35, 120.57, 115.70, 114.22, 55.39, 12.68. HRMS *m/z* calcd. for $C_{16}H_{14}NO_3S$ $[M+H]^+$ 300.0694, found 300.0696.

5-Methyl-N,3-diphenylisoxazole-4-carboxamide (3n)

Yield: 80%. White crystal. Mp 198–200 °C. ^{1}H NMR and ^{13}C NMR spectra matched with literature [1]. ^{1}H NMR (400 MHz, $CDCl_3$) δ 7.68 (m, 2H), 7.63 – 7.52 (m, 3H), 7.28 (m, 2H), 7.23 (m, 2H), 7.12 (brs, 1H), 7.10 (m, 1H), 2.80 (s, 3H). ^{13}C NMR (101 MHz, $CDCl_3$) δ 175.31, 159.83, 159.34, 137.19, 130.85, 129.44, 129.26, 129.04, 128.01, 124.64, 119.60, 111.24, 13.18. HRMS *m/z* calcd. for $C_{17}H_{15}N_2O_2$ $[M+H]^+$ 279.1134, found 279.1139.

3-(4-Fluorophenyl)-5-methyl-N-phenylisoxazole-4-carboxamide (3o)

Yield: 82%. White crystal. Mp 172–174 °C. ^{1}H NMR and ^{13}C NMR spectra matched with literature [1]. ^{1}H NMR (400 MHz, $CDCl_3$) δ 7.72 – 7.57 (m, 4H), 7.32 – 7.20 (m, 3H), 7.18 – 7.05 (m, 2H), 2.82 (s, 3H), ^{13}C NMR (101 MHz, $CDCl_3$) δ 175.14, 164.29 (d, J = 250 Hz), 159.44, 159.12, 137.16, 131.40 (d, J = 8.8 Hz), 129.28, 125.01, 124.09 (d, J = 3.7 Hz), 119.80, 116.77 (d, J = 22 Hz), 111.48, 13.21. ^{19}F NMR (471 MHz, $CDCl_3$) δ -108.64. HRMS *m/z* calcd. for $C_{17}H_{14}FN_2O_2$ $[M+H]^+$ 297.1039, found 297.1045.

3-(4-Methoxyphenyl)-5-methyl-N-phenylisoxazole-4-carboxamide (3p)

Yield: 78%. White crystal. Mp 144–146 °C. ^{1}H NMR (400 MHz, $CDCl_3$) δ 7.59 (d, J = 8.7 Hz, 2H), 7.36 – 7.19 (m, 4H), 7.15 – 7.04 (m, 3H), 3.89 (s, 3H), 2.77 (s, 3H); ^{13}C NMR (101 MHz, $CDCl_3$) δ 175.26, 161.68, 159.69, 137.37, 130.81, 129.18, 124.76, 119.94, 119.83, 114.99, 111.25, 55.64, 13.30. HRMS *m/z* calcd. for $C_{18}H_{17}N_2O_3$ $[M+H]^+$ 309.1239, found 309.1243.

Benzyl 5-methyl-3-phenylisoxazole-4-carboxylate (3q)

Yield: 85%. White solid. Mp 33–35 °C. ^{1}H NMR (400 MHz, $CDCl_3$) δ 7.67 – 7.57 (m, 2H), 7.52 – 7.46 (m, 1H), 7.46 – 7.38 (m, 2H), 7.34 (m, 3H), 7.28 – 7.19 (m, 2H), 5.25 (s, 2H), 2.75 (s, 3H); ^{13}C NMR (101 MHz, $CDCl_3$) δ 176.21, 162.69, 161.89, 135.32, 129.79, 129.48, 128.65, 128.53, 128.44, 128.39, 128.16, 108.44, 66.61, 13.77. HRMS *m/z* calcd. for $C_{18}H_{16}NO_3$ $[M+H]^+$ 294.1130, found 294.1143.

Benzyl 3-(4-fluorophenyl)-5-methylisoxazole-4-carboxylate (3r)

Yield: 90%. White solid. Mp 49–51 °C. ^{1}H NMR (400 MHz, $CDCl_3$) δ 7.59 (t, J = 8.7, 2H), 7.36 (m, 3H), 7.25 (m, 2H), 7.07 (t, J = 8.7 Hz, 2H), 5.24 (s, 2H), 2.74 (s, 3H); ^{13}C NMR (101 MHz,

$CDCl_3$) δ 176.41, 163.84 (d, *J* = 250 Hz), 161.86, 161.80, 135.21, 131.54 (d, *J* = 8.0 Hz), 128.72, 128.62, 128.53, 124.55 (d, *J* = 4.0 Hz), 115.28 (d, *J* = 22 Hz), 108.37, 66.76, 13.84. ^{19}F NMR (101 MHz, $CDCl_3$) δ -111.14. HRMS *m/z* calcd. for $C_{18}H_{15}FNO_3$ $[M+H]^+$ 312.1036, found 312.1032.

Benzyl 3-(4-methoxyphenyl)-5-methylisoxazole-4-carboxylate (3s)

Yield: 75%. White solid. Mp 47–49 °C. ^{1}H NMR (400 MHz, $CDCl_3$) δ 7.56 (d, *J* = 8.7 Hz, 2H), 7.35 (m, 3H), 7.29 – 7.24 (m, 2H), 6.92 (d, *J* = 8.7 Hz, 2H), 5.26 (s, 2H), 3.86 (s, 3H), 2.73 (d, *J* = 1.0 Hz, 3H); ^{13}C NMR (101 MHz, $CDCl_3$) δ 176.01, 162.23, 161.93, 160.81, 135.28, 130.79, 128.55, 128.38, 128.37, 120.58, 113.54, 108.19, 66.51, 55.30, 13.75. HRMS *m/z* calcd. for $C_{19}H_{18}NO_4$ $[M+H]^+$ 324.1236, found 324.1236.

Ethyl 3-(4-fluorophenyl)-5-methylisoxazole-4-carboxylate (3t)

Yield: 86%. White crystal. Mp 58–60 °C. ^{1}H NMR (400 MHz, $CDCl_3$) δ 7.70 – 7.59 (m, 2H), 7.20 – 7.08 (m, 2H), 4.26 (q, *J* = 7.1 Hz, 2H), 2.73 (s, 3H), 1.26 (t, *J* = 7.1 Hz, 3H). ^{13}C NMR (101 MHz, $CDCl_3$) δ 175.96, 163.72 (d, *J* = 250 Hz), 161.85, 161.70, 131.43 (d, *J* = 8.6 Hz), 124.56 (d, *J* = 3.6 Hz), 115.07 (d, *J* = 21 Hz), 108.35, 60.78, 14.00, 13.61. ^{19}F NMR (101 MHz, $CDCl_3$) δ -111.22. HRMS *m/z* calcd. for $C_{13}H_{13}FNO_3$ $[M+H]^+$ 250.0879, found 250.0878.

***N*-Benzyl-5-methyl-3-phenylisoxazole-4-carboxamide (3u)**

Yield: 92%. White crystal. Mp 135–137 °C. ^{1}H NMR (400 MHz, $CDCl_3$) δ 7.54 – 7.48 (m, 2H), 7.44 (d, *J* = 7.5 Hz, 1H), 7.39 (dd, *J* = 7.2, 1.2 Hz, 2H), 7.32 – 7.24 (m, 3H), 7.09 (dd, *J* = 7.3, 2.3 Hz, 2H), 5.73 (s, 1H), 4.42 (d, *J* = 5.7 Hz, 2H), 2.73 (s, 3H); ^{13}C NMR (101 MHz, $CDCl_3$) δ 174.27, 161.47, 160.19, 137.48, 130.48, 129.19, 129.08, 128.79, 128.15, 127.82, 127.71, 111.12, 43.67, 13.05. HRMS *m/z* calcd. for $C_{18}H_{17}N_2O_2$ $[M+H]^+$ 293.1290, found 293.1294.

***N*-Benzyl-3-(4-fluorophenyl)-5-methylisoxazole-4-carboxamide (3v)**

Yield: 82%. White crystal. Mp 179–181 °C. ^{1}H NMR (400 MHz, $CDCl_3$) δ 7.51 (dd, *J* = 8.8, 5.2 Hz, 2H), 7.35 – 7.27 (m, 2H), 7.13 (dd, *J* = 7.3, 2.3 Hz, 2H), 7.05 (t, *J* = 8.6 Hz, 2H), 5.62 (s, 1H), 4.44 (d, *J* = 5.7 Hz, 2H), 2.71 (s, 3H); ^{13}C NMR (101 MHz, $CDCl_3$) δ 174.04, 164.07 (d, *J* = 250 Hz), 161.38, 159.37, 137.47, 131.13 (d, *J* = 8.0 Hz), 128.92, 127.94 (d, *J* = 2.0 Hz), 124.18 (d, *J* =

3.0 Hz), 116.48, 116.26, 111.28, 43.82, 12.99. ^{19}F NMR (101 MHz, $CDCl_3$) δ -109.57. HRMS *m/z* calcd. for $C_{18}H_{16}FN_2O_2$ $[M+H]^+$ 311.1196, found 311.1194.

***N*-Benzyl-3-(4-methoxyphenyl)-5-methylisoxazole-4-carboxamide (3w)**

Yield: 74%. White crystal. Mp 143–145 °C. ^{1}H NMR (400 MHz, $CDCl_3$) δ 7.46 – 7.39 (m, 2H), 7.27 (dt, *J* = 6.3, 2.2 Hz, 3H), 7.15 – 7.08 (m, 2H), 6.85 (d, *J* = 8.8 Hz, 2H), 5.78 (s, 1H), 4.42 (d, *J* = 5.7 Hz, 2H), 3.80 (s, 3H), 2.70 (s, 3H); ^{13}C NMR (101 MHz, $CDCl_3$) δ 174.07, 161.62, 161.30, 159.90, 137.60, 130.48, 128.77, 127.91, 127.69, 120.05, 114.61, 111.04, 55.47, 43.63, 13.03. HRMS *m/z* calcd. for $C_{19}H_{19}N_2O_3$ $[M+H]^+$ 323.1396, found 323.1394.

(3-(4-Fluorophenyl)-5-(trifluoromethyl)isoxazol-4-yl)(phenyl)methanone (3x)

General synthetic procedure for the [3 + 2]-cycloaddition reaction was followed but with 5% water, 95% methanol as the solvent mixture. Yield: 40%. Sticky gel. ^{1}H NMR (500 MHz, $CDCl_3$) δ 7.86 – 7.78 (m, 2H), 7.69 – 7.60 (m, 1H), 7.60 – 7.53 (m, 2H), 7.47 (t, *J* = 7.9 Hz, 2H), 7.04 (t, *J* = 8.6 Hz, 2H); ^{13}C NMR (126 MHz, $CDCl_3$) δ 187.00, 164.34 (d, *J* = 250 Hz), 160.85, 156.77 (q, *J* = 42 Hz), 136.12, 135.26, 130.37 (d, *J* = 8.8 Hz), 129.79, 129.26, 122.67 (d, *J* = 2.5 Hz), 118.73, 118.72 (d, *J* = 2.5 Hz), 116.50 (d, *J* = 21 Hz); ^{19}F NMR (471 MHz, $CDCl_3$) δ -62.50, -108.50. HRMS *m/z* calcd. for $C_{17}H_{10}F_4NO_2$ $[M+H]^+$ 336.0648, found 336.0656.

(3-(4-Methoxyphenyl)-5-(trifluoromethyl)isoxazol-4-yl)(phenyl)methanone (3y)

General synthetic procedure for the [3 + 2]-cycloaddition reaction was followed but with 5% water, 95% methanol as the solvent mixture. Yield: 35%. Sticky gel. ^{1}H NMR (500 MHz, $CDCl_3$) δ 7.86 – 7.79 (m, 2H), 7.67 – 7.58 (m, 1H), 7.54 – 7.43 (m, 4H), 6.90 – 6.82 (m, 2H), 3.78 (s, 3H); ^{13}C NMR (126 MHz, $CDCl_3$) δ 187.40, 161.70, 161.28, 156.39 (q, *J* = 42 Hz), 136.24, 135.09, 129.81 (d, *J* = 3.8 Hz), 129.70, 129.20, 118.77 (d, *J* = 5.0 Hz), 118.65, 116.63, 114.66, 55.46; ^{19}F NMR (471 MHz, $CDCl_3$) δ -62.55. HRMS *m/z* calcd. for $C_{18}H_{13}F_3NO_3$ $[M+H]^+$ 348.0848, found 348.0826.

(3-(4-Methoxyphenyl)-5-(trifluoromethyl)isoxazol-4-yl)(naphthalen-1-yl)methanone (3z)

General synthetic procedure for the [3 + 2]-cycloaddition reaction was followed but with 5% water, 95% methanol as the solvent mixture. Yield: 40%. Yellow sticky gel. ^{1}H NMR (400 MHz, $CDCl_3$)

δ 8.30 – 8.23 (m, 1H), 8.04 (dd, *J* = 8.7, 1.8 Hz, 1H), 8.01 – 7.85 (m, 3H), 7.72 – 7.51 (m, 4H), 6.90 – 6.80 (m, 2H), 3.76 (s, 3H). ^{13}C NMR (126 MHz, $CDCl_3$) δ 187.24, 161.68, 161.38, 156.36 (q, *J* = 42 Hz), 136.49, 133.77, 133.21, 132.42, 130.04, 129.81, 129.65, 129.37, 128.04, 127.43, 123.83, 118.77, 118.75, 116.69, 114.68, 55.27. ^{19}F NMR (471 MHz, $CDCl_3$) δ -62.48. HRMS *m/z* calcd. for $C_{22}H_{15}F_3NO_3$ $[M+H]^+$ 398.1004, found 398.1027.

(3-(4-Fluorophenyl)-5-(trifluoromethyl)isoxazol-4-yl)(naphthalen-1-yl)methanone (3aa)

General synthetic procedure for the [3 + 2]-cycloaddition reaction was followed but with 5% water, 95% methanol as the solvent mixture. Yield: 40%. Yellow sticky gel. ^{1}H NMR (400 MHz, $CDCl_3$) δ 8.30 – 8.15 (m, 1H), 8.15 – 7.82 (m, 4H), 7.73 – 7.48 (m, 4H), 7.10 – 6.98 (m, 2H). ^{13}C NMR (101 MHz, $CDCl_3$) δ 186.83, 164.31 (d, *J* = 250 Hz), 160.94, 156.73 (q, *J* = 42 Hz), 136.54, 133.66, 133.21, 132.38, 130.32 (d, *J* = 8.0 Hz), 129.96 (d, *J* = 6.0 Hz), 129.47, 128.07, 127.53, 123.74, 122.72 (d, *J* = 3.0 Hz), 119.04, 118.83 (d, *J* = 2.0 Hz), 116.60, 116.38. ^{19}F NMR (377 MHz, $CDCl_3$) δ -62.41, -108.43. HRMS *m/z* calcd. for $C_{21}H_{12}F_4NO_2$ $[M+H]^+$ 386.0804, found 386.0825.

Reference

1. Zhou, X.; Xu, X.; Shi, Z.; Liu, K.; Gao, H.; Li, W. *Org. Biomol. Chem.* **2016**, *14*, 5246–5250.

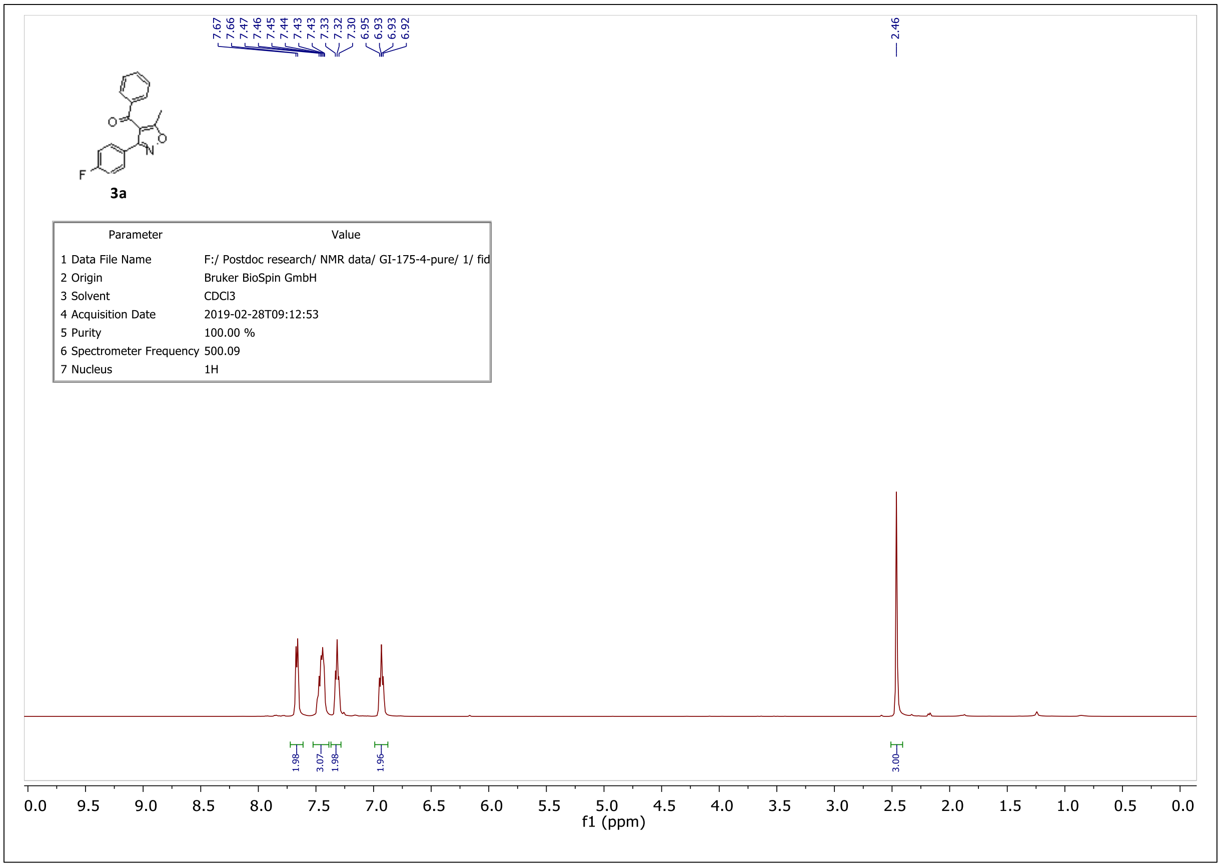

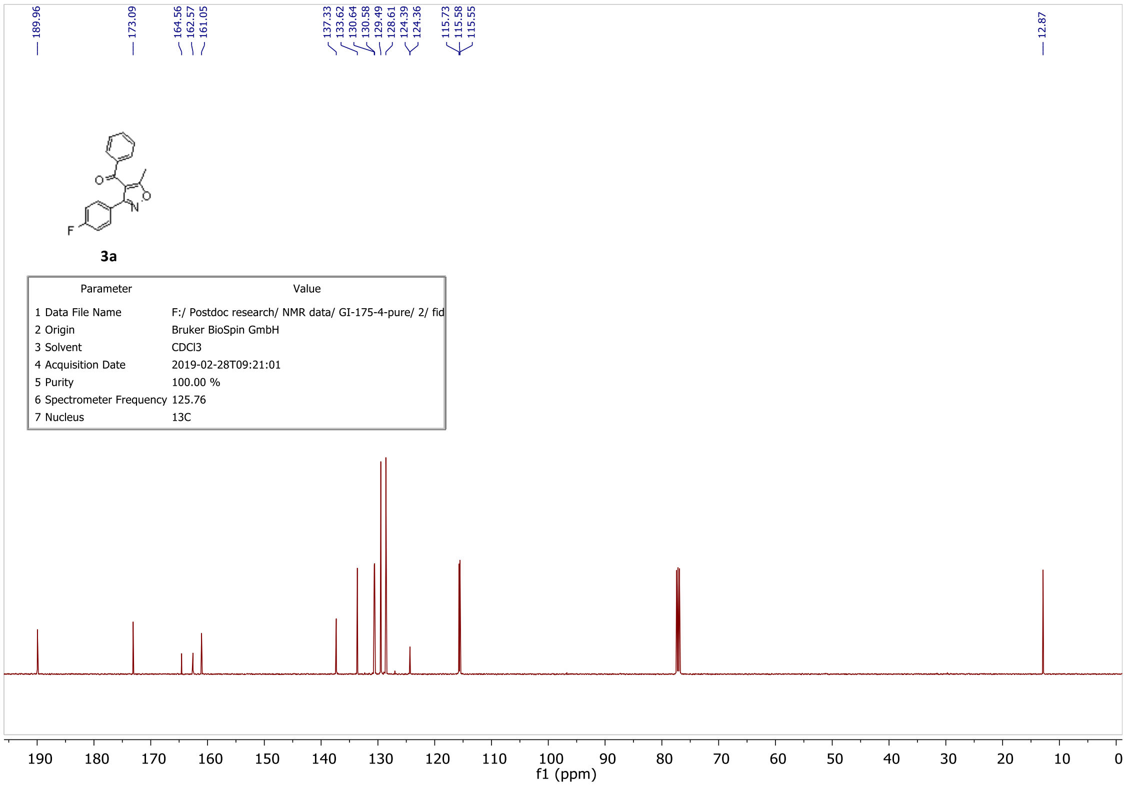

Figure S3: ^{1}H and ^{13}C NMR spectra of compound **3a**.

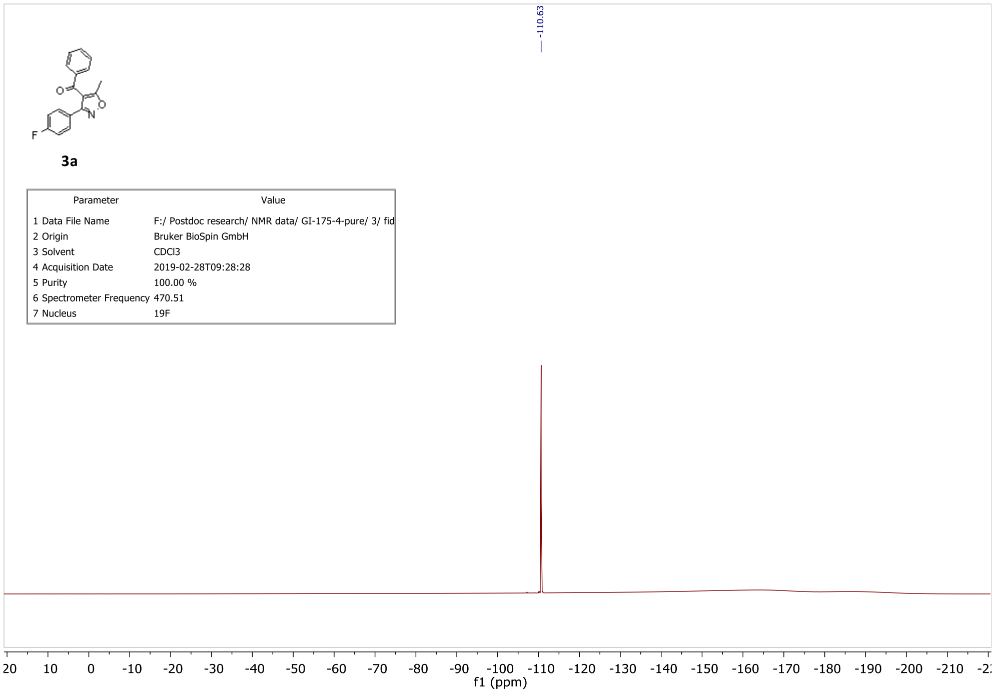

Figure S4: ^{19}F NMR spectrum of compound **3a**.

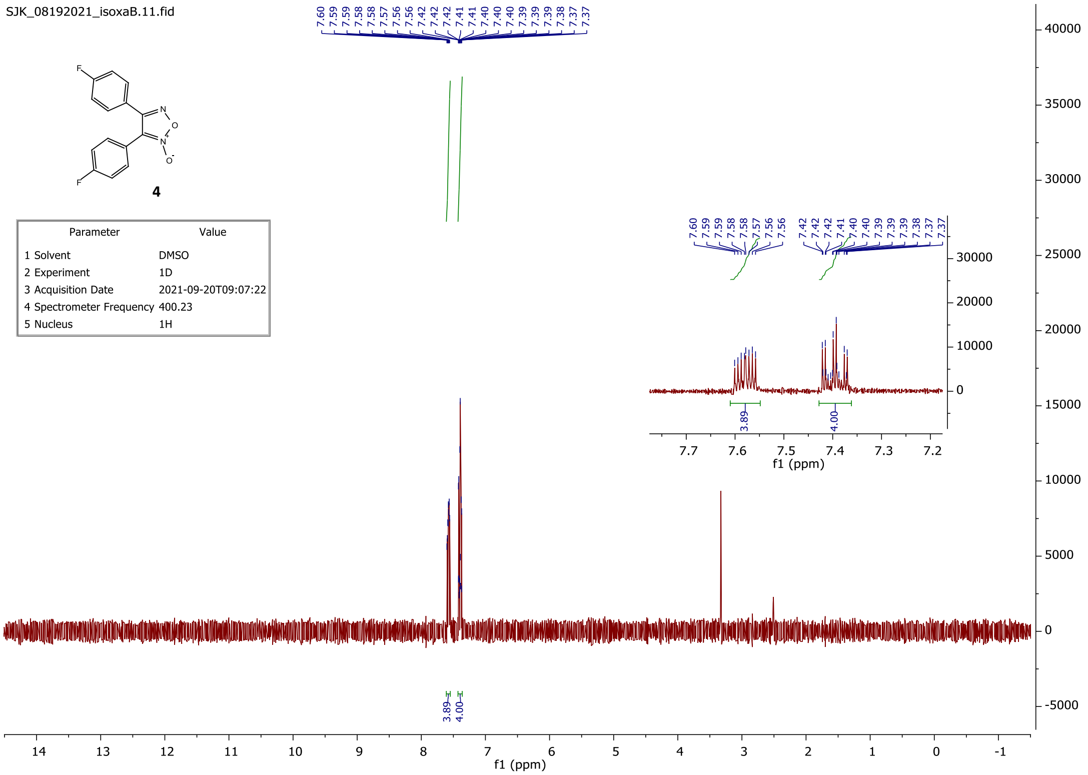

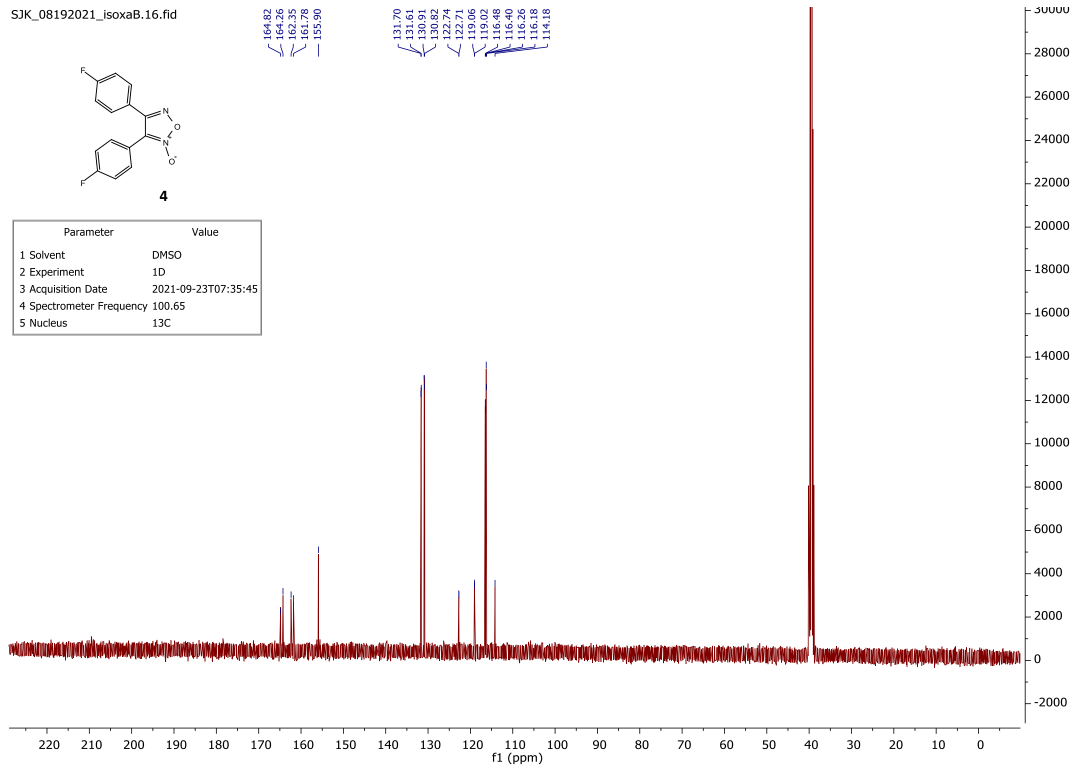

Figure S5: ^{1}H and ^{13}C NMR spectra of compound **4**.

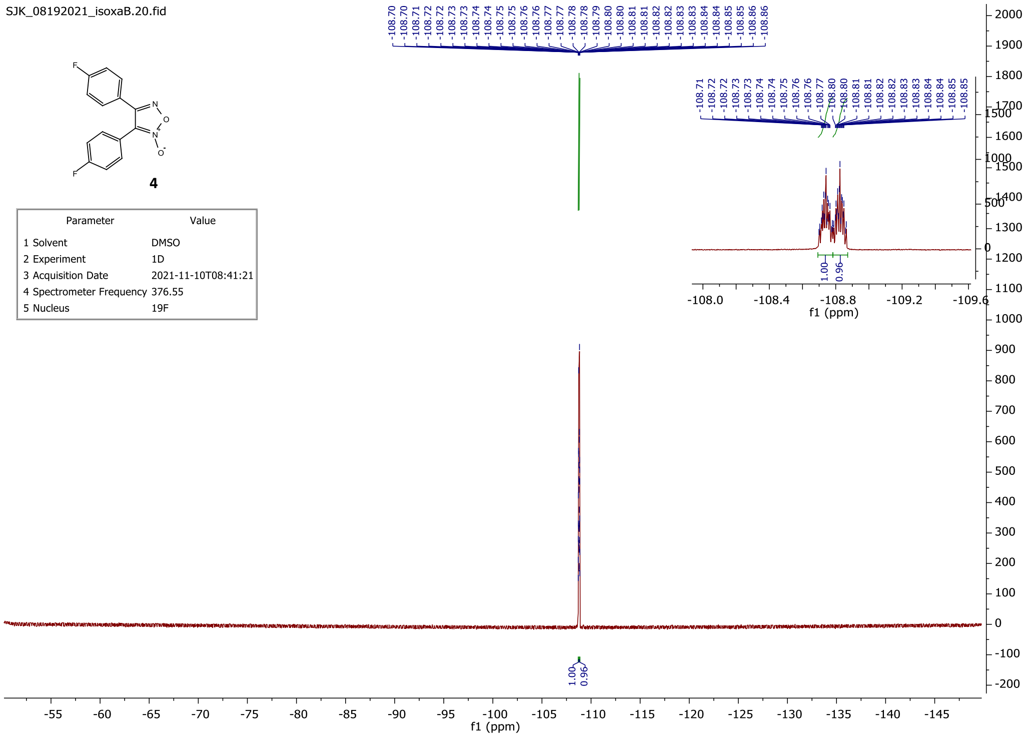

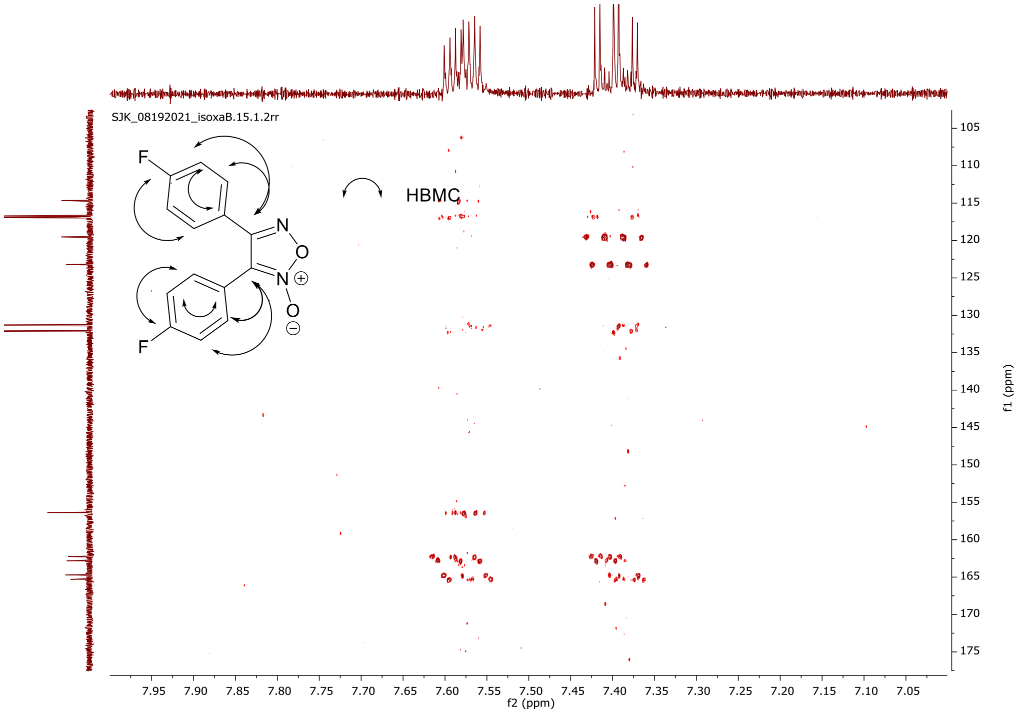

Figure S6: ^{19}F NMR and HMBC spectra of compound **4**. Compound **4** has a molecular formula of $C_{14}H_8N_2O_2F_2$, as evidenced from DART-HRMS analysis (*m/z* calcd. for $C_{14}H_9N_2O_2F_2$ $[M+H]^+$ 275.0632, found 275.0637). The oxadiazole and fluorophenyl groups are revealed by ^{19}F and HMBC spectral data, which identified **4** as 3,4-bis(4-fluorophenyl)-1,2,5-oxadiazole 2-oxide.

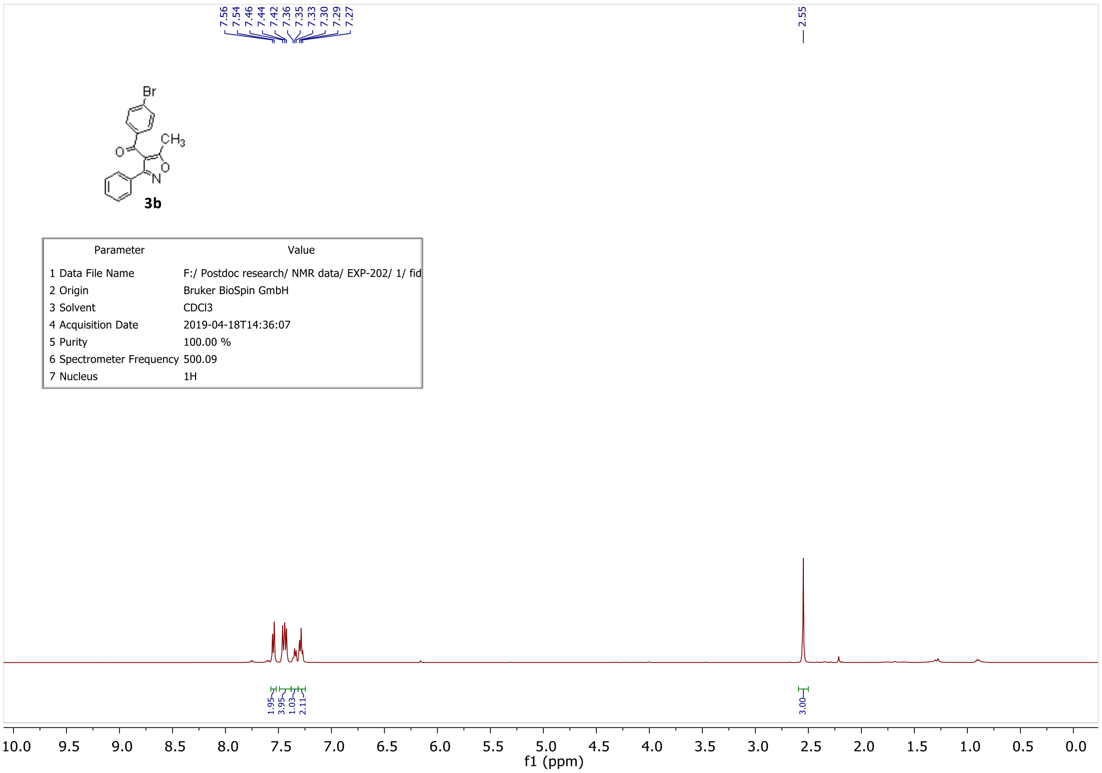

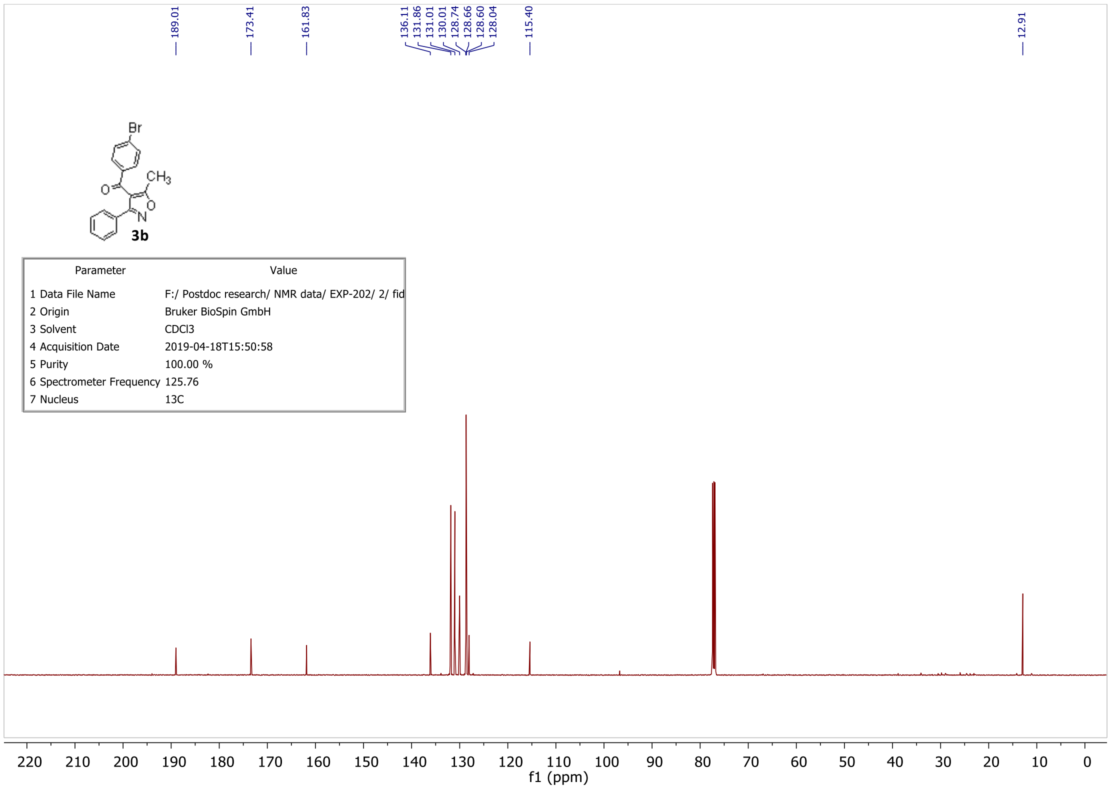

Figure S7: ^{1}H and ^{13}C NMR spectra of compound **3b**.

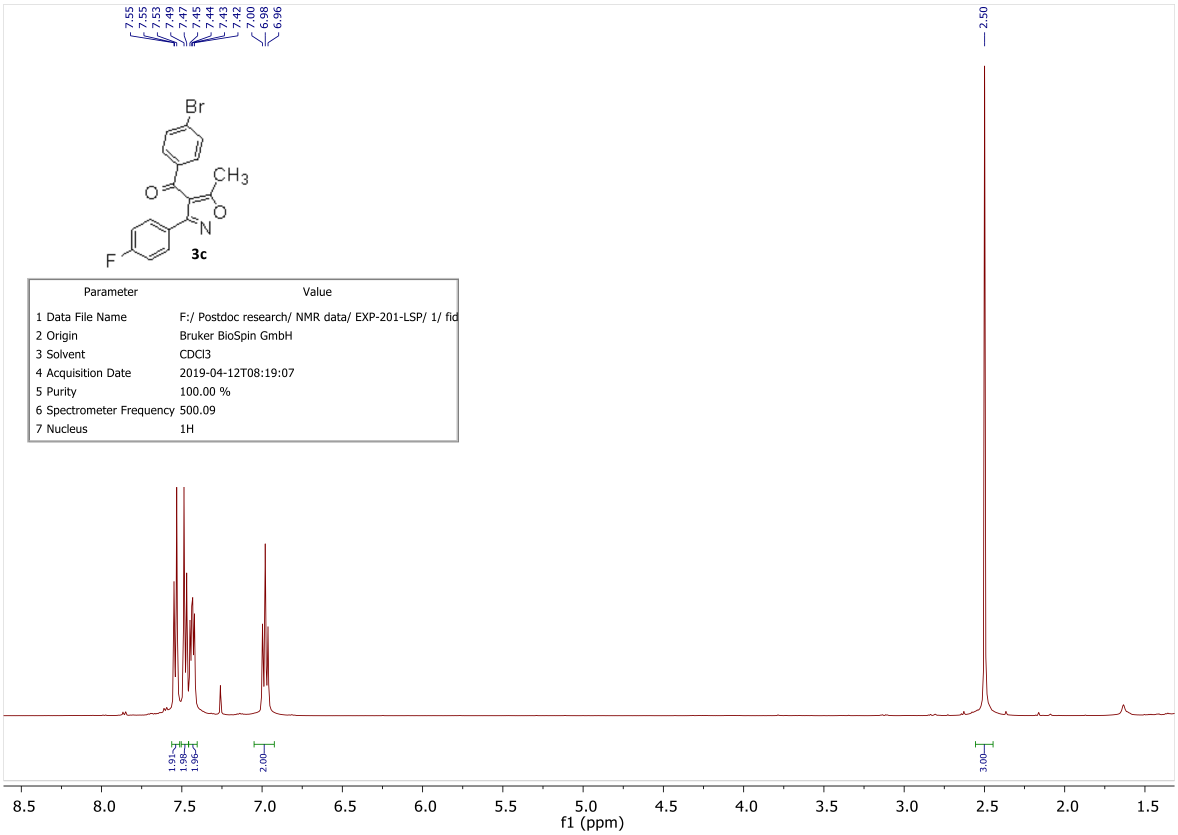

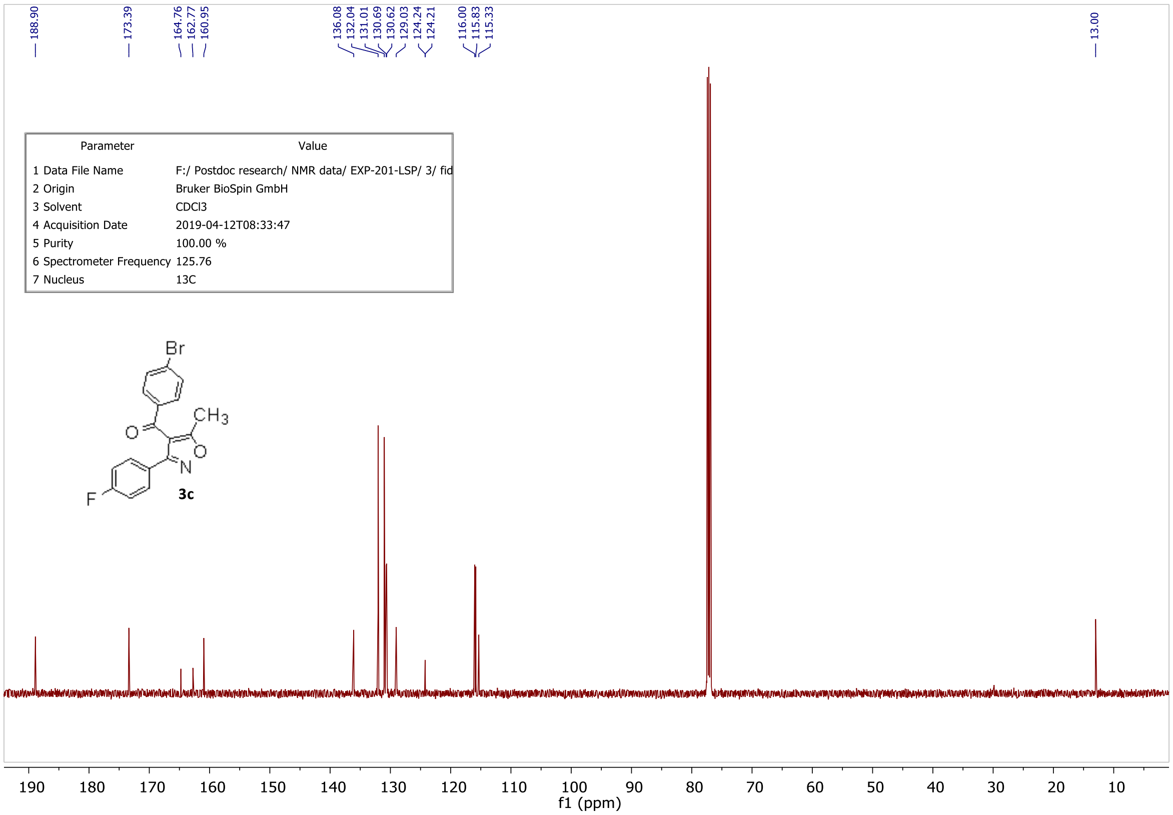

Figure S8: ^{1}H and ^{13}C NMR spectra of compound **3c**.

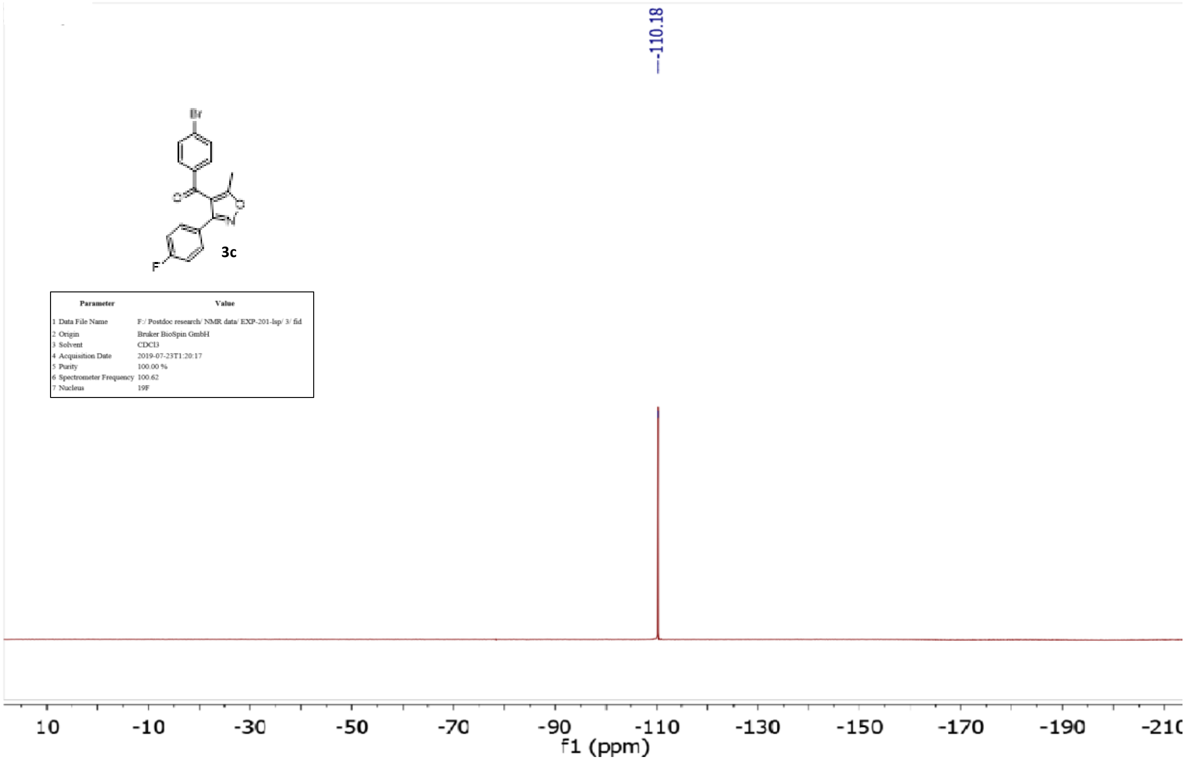

Figure S9: ^{19}F NMR spectrum of compound **3c**.

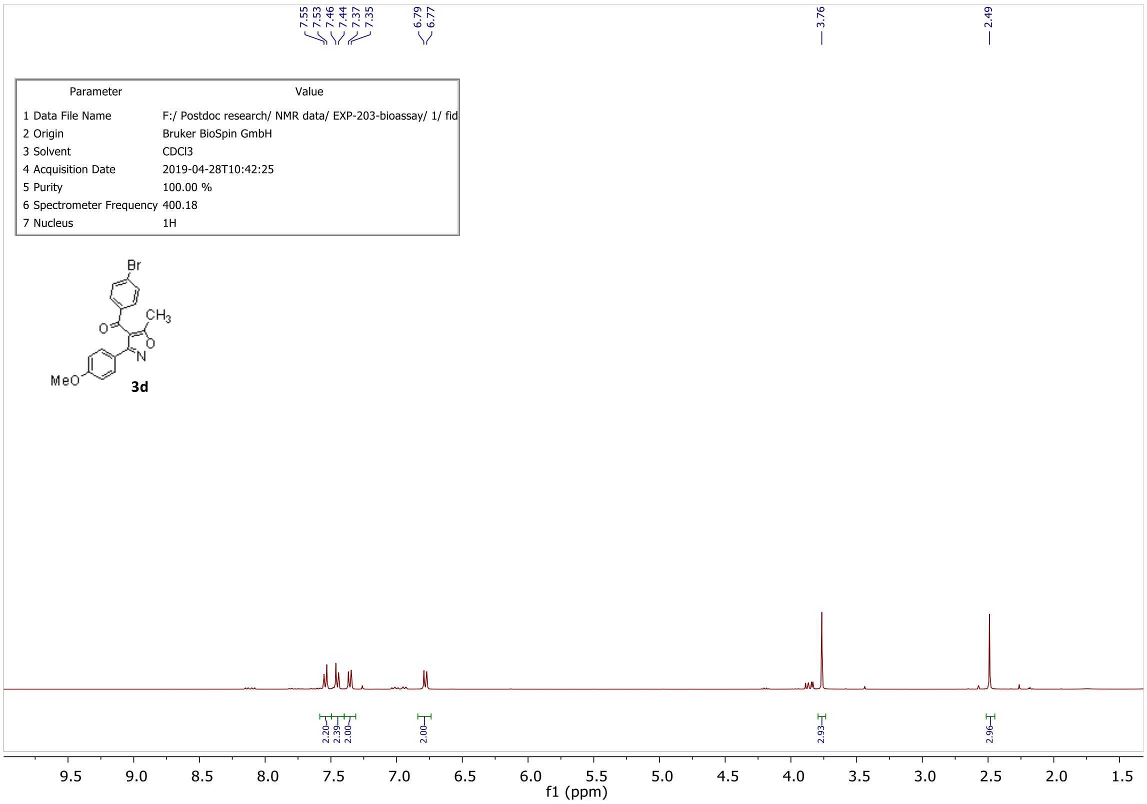

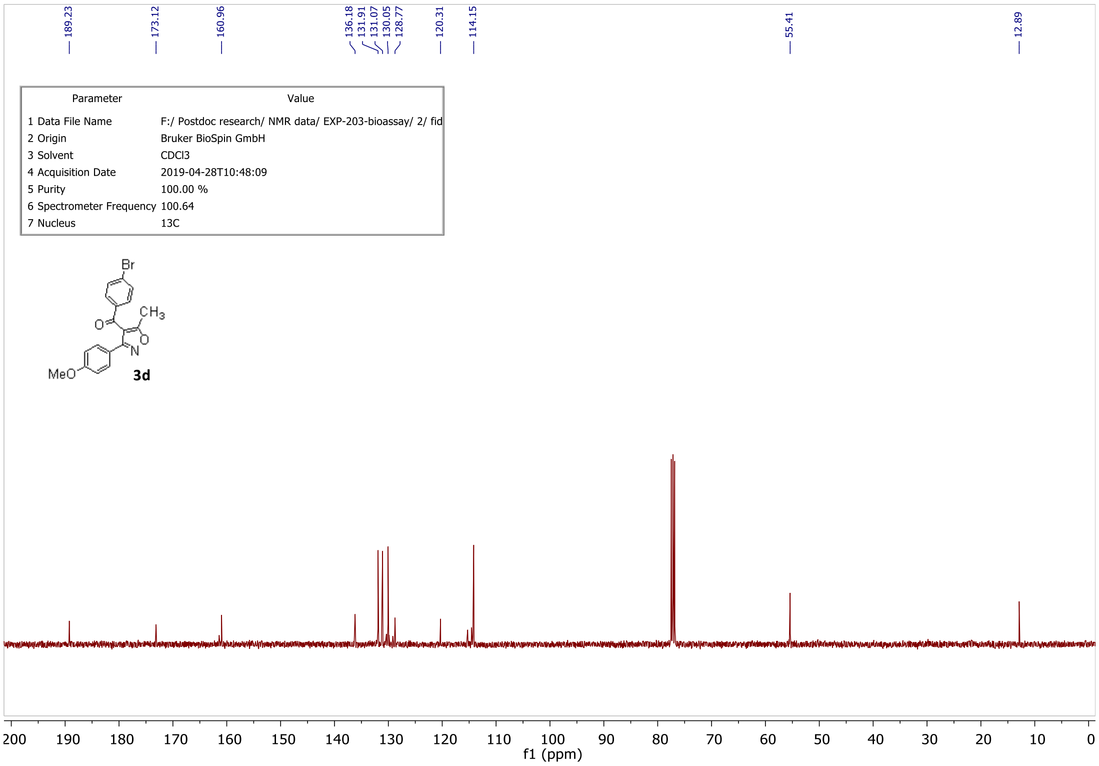

Figure S10: ^{1}H and ^{13}C NMR spectra of compound **3d**.

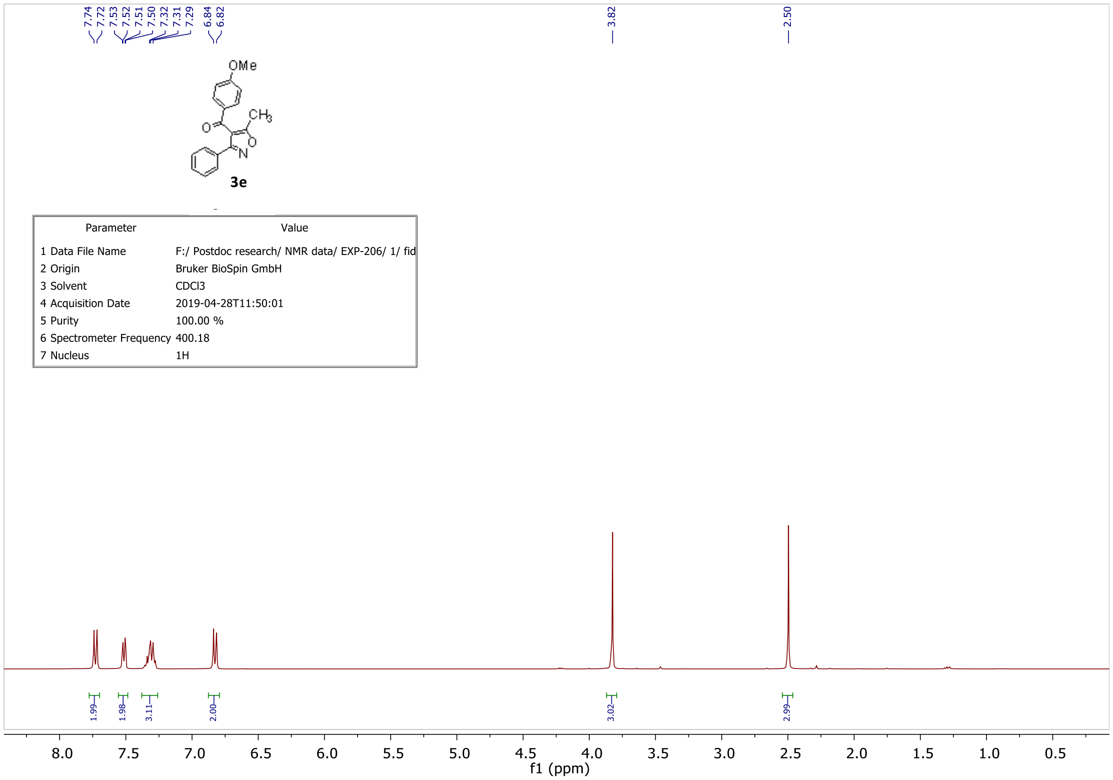

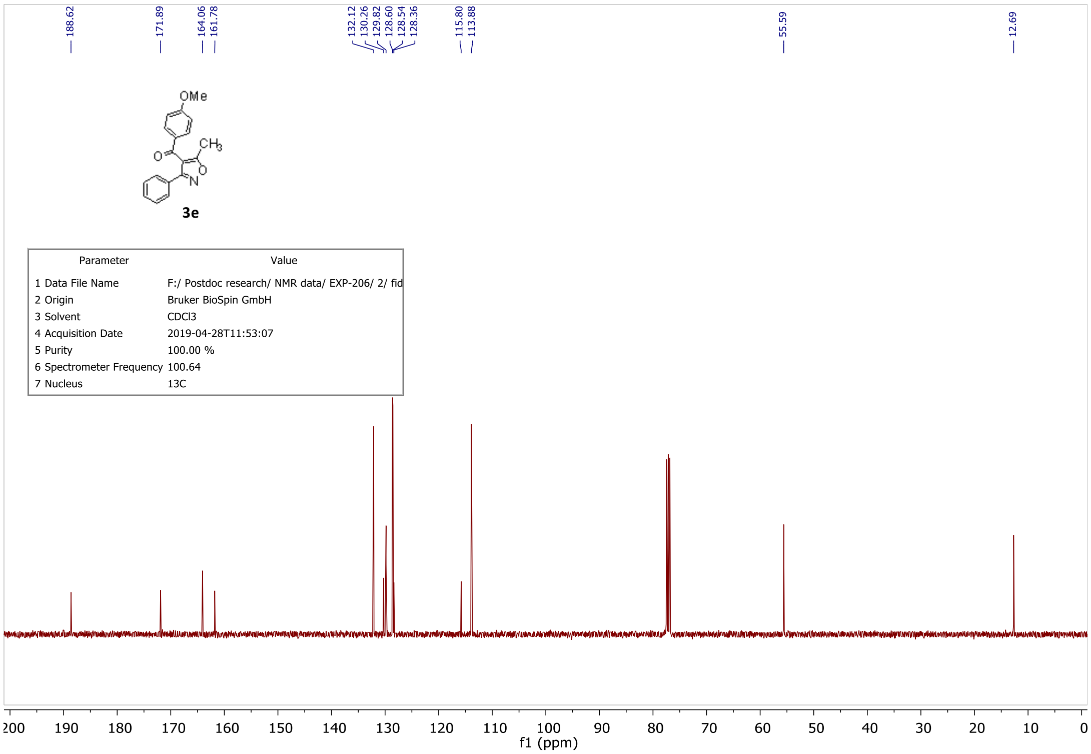

Figure S11: ^{1}H and ^{13}C NMR spectra of compound **3e**.

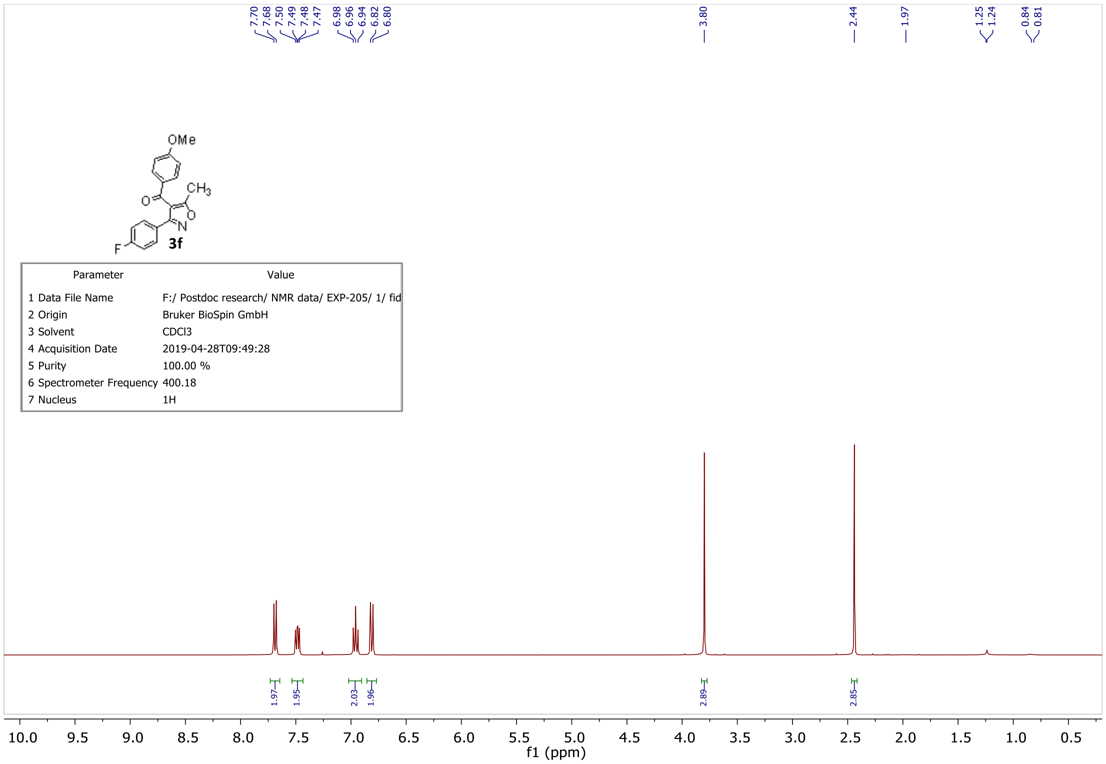

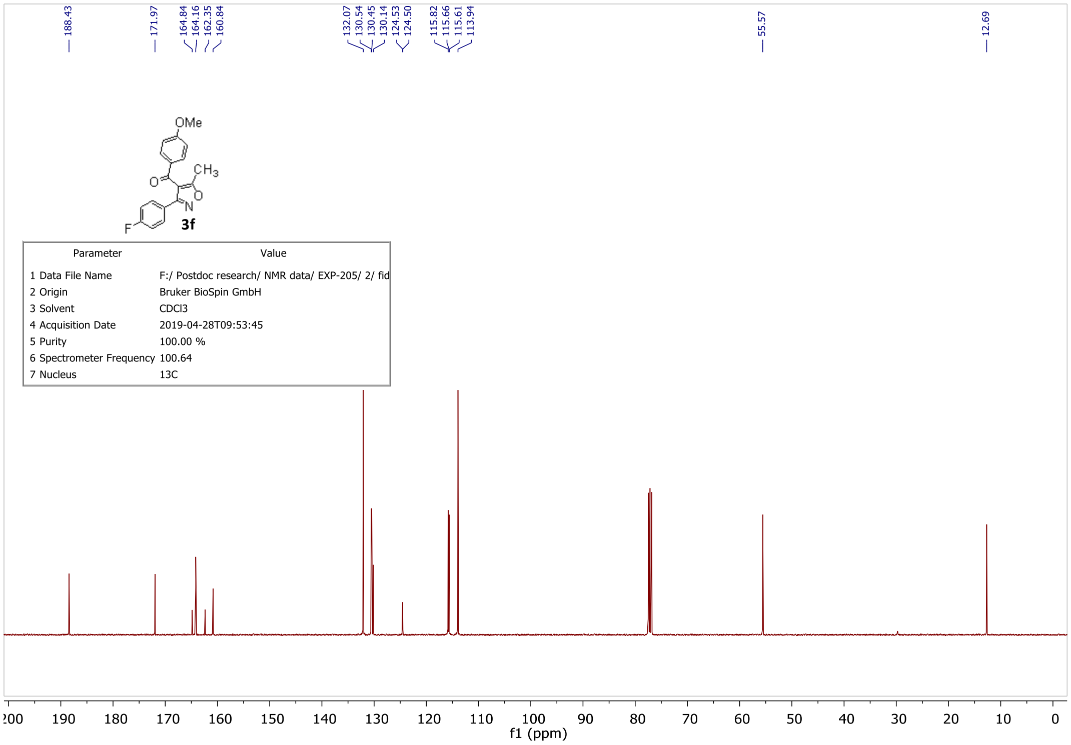

Figure S12: ^{1}H and ^{13}C NMR spectra of compound **3f**.

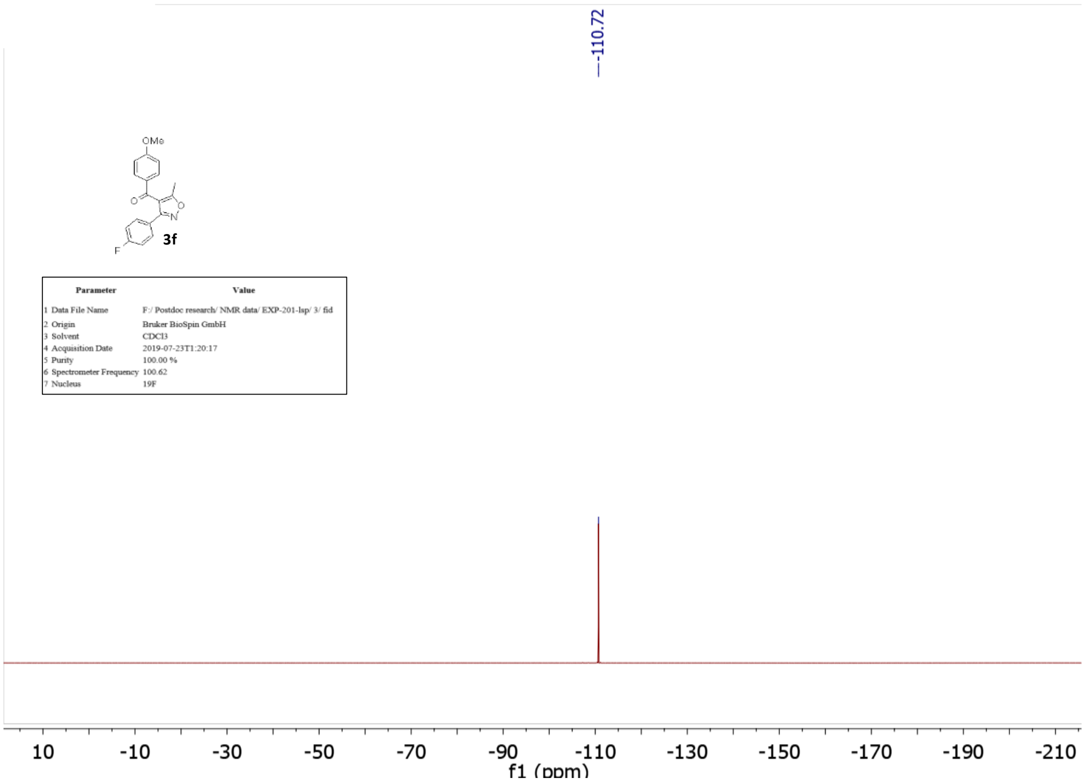

Figure S13: ^{19}F NMR spectrum of compound **3f**.

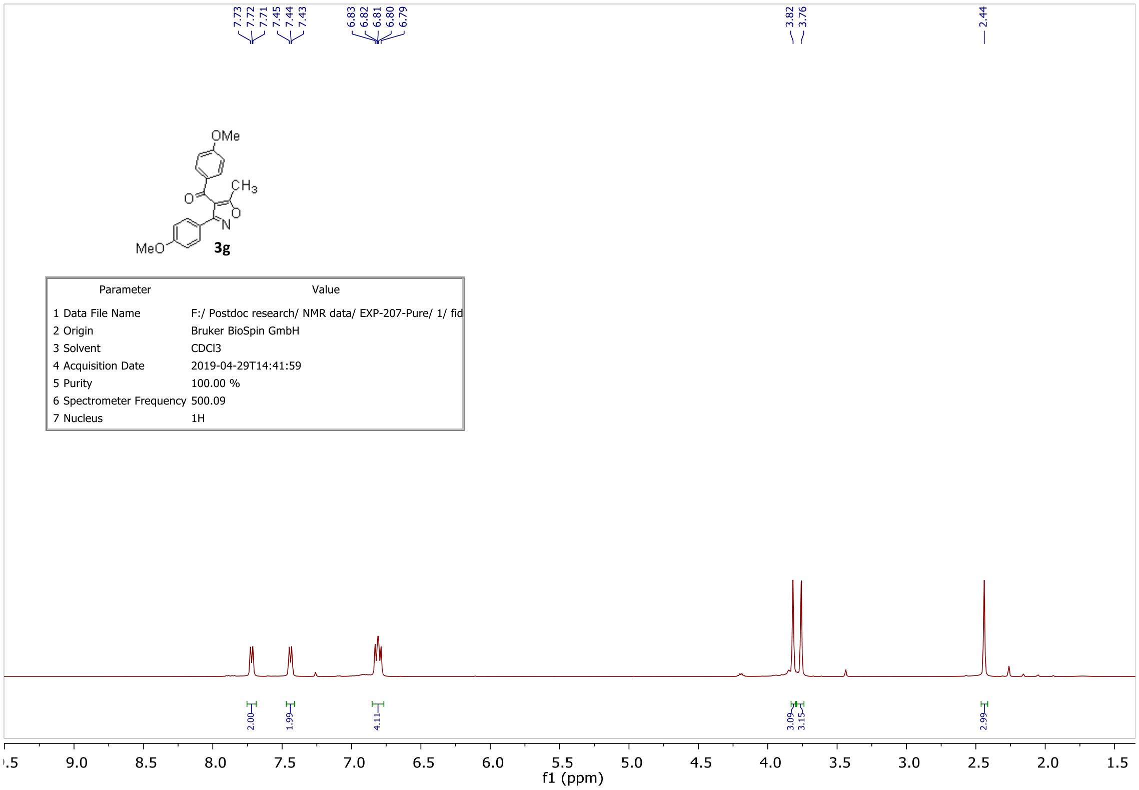

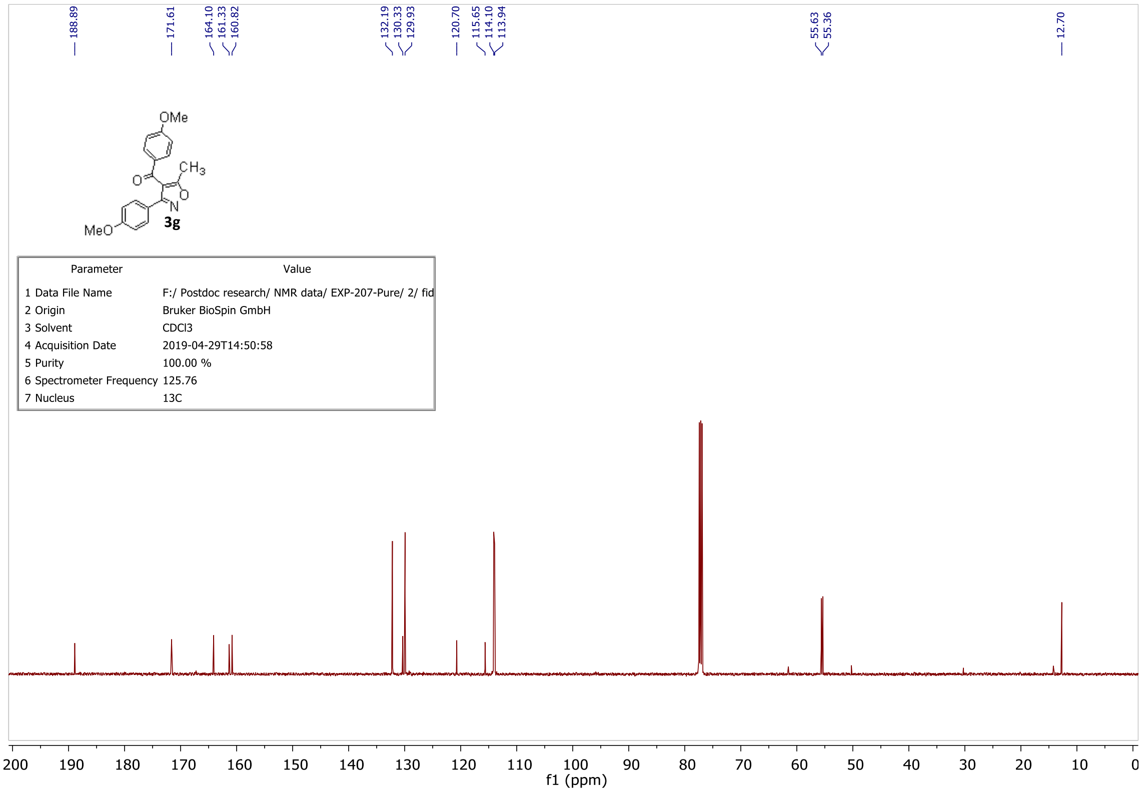

Figure S14: ^{1}H and ^{13}C NMR spectra of compound **3g**.

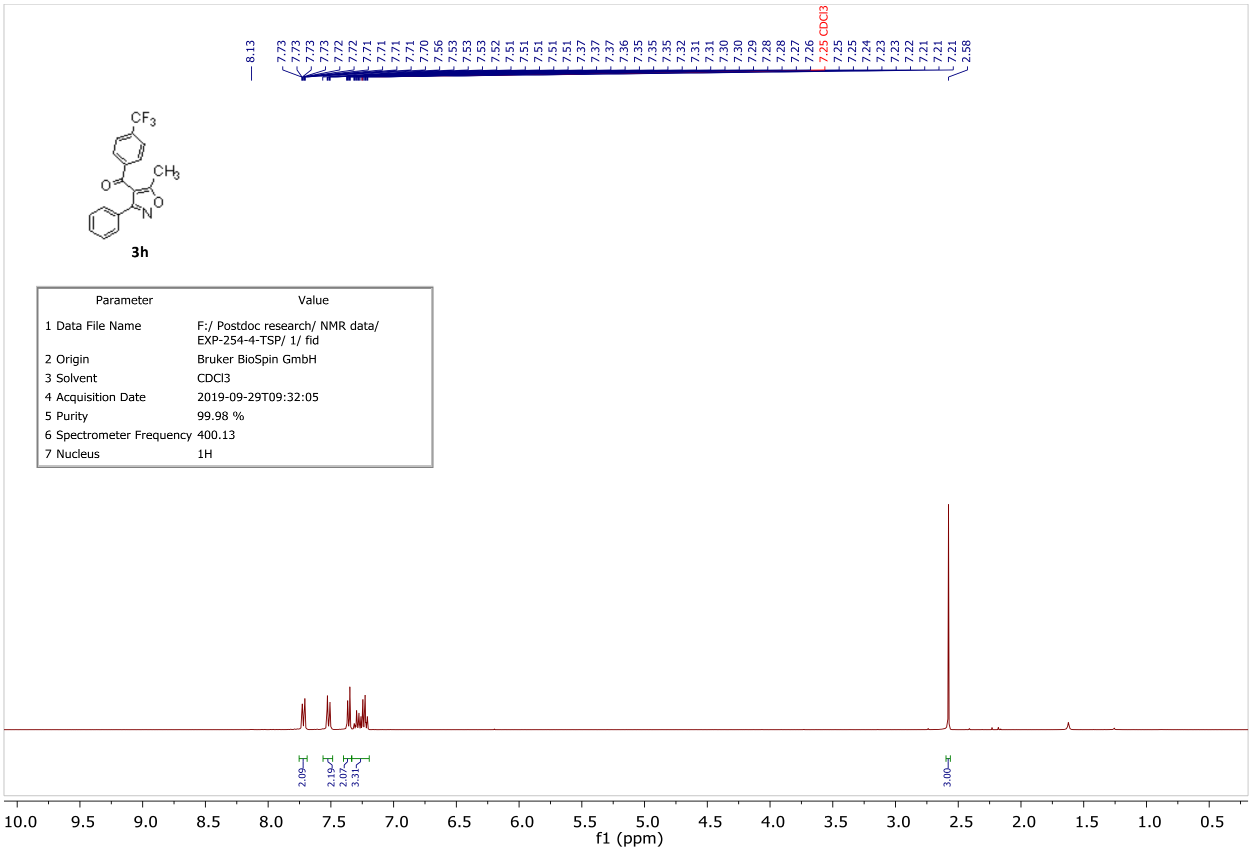

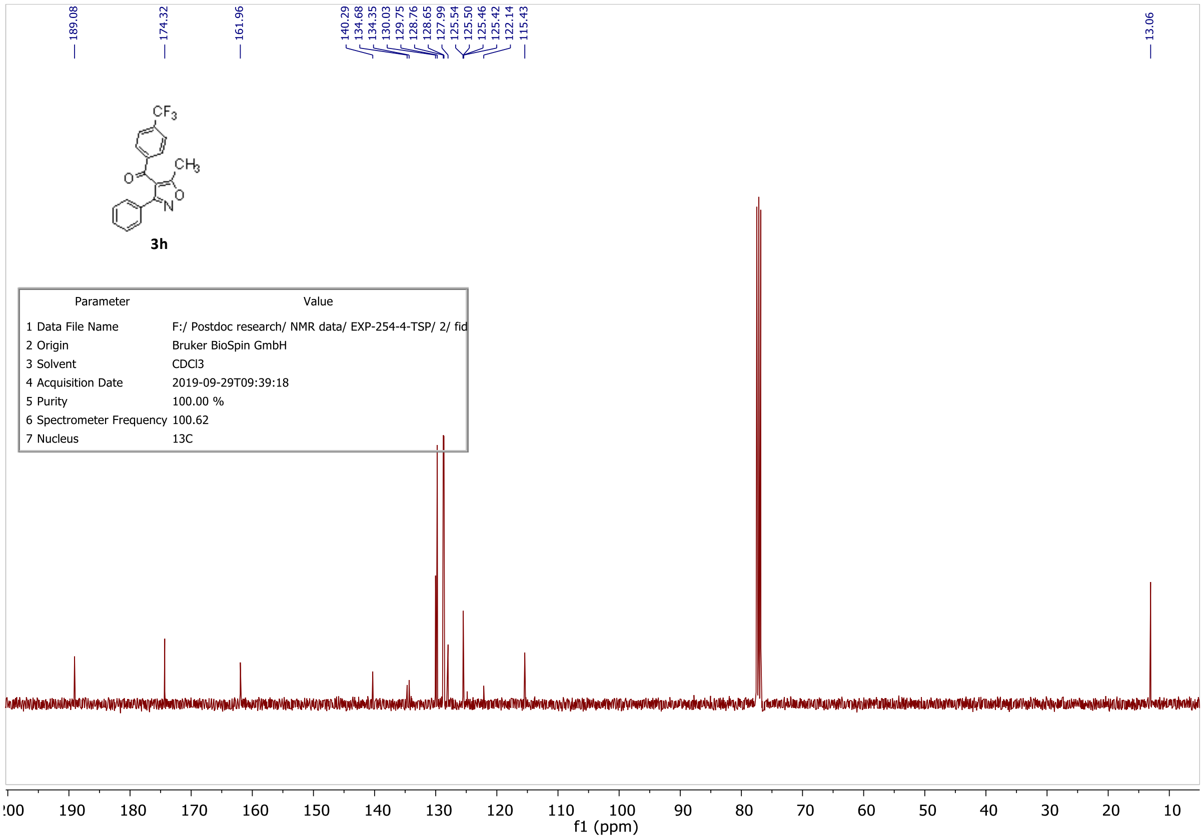

Figure S15: ^{1}H and ^{13}C NMR spectra of compound **3h**.

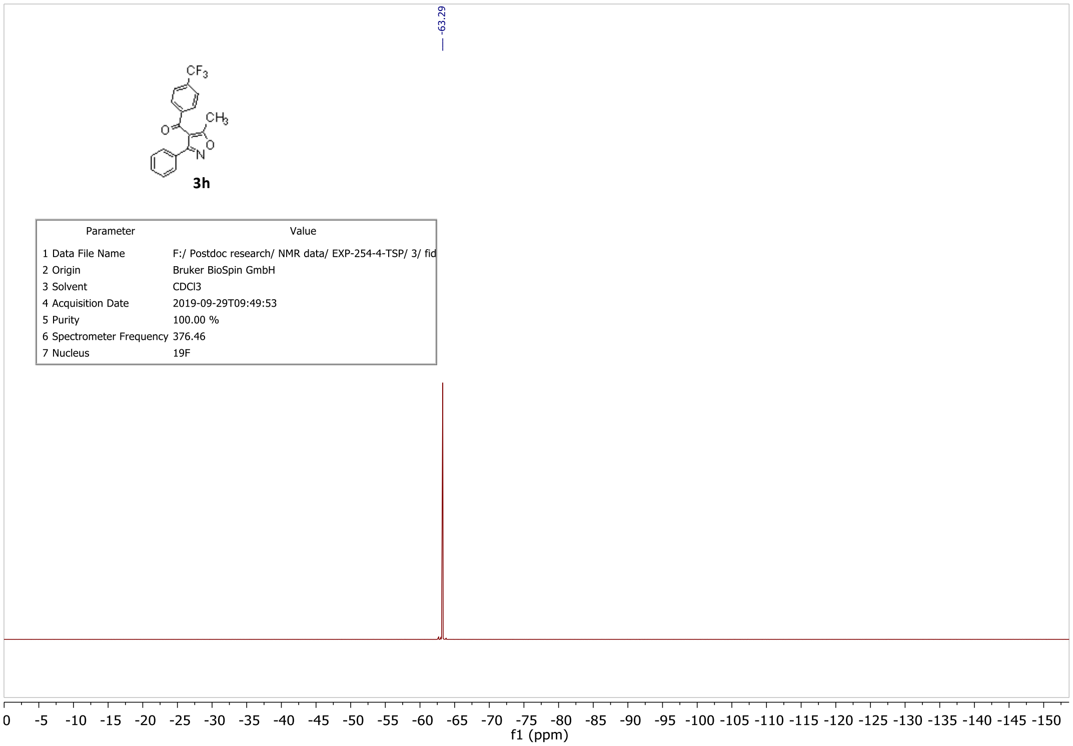

Figure S16: ^{19}F NMR spectrum of compound **3h**.

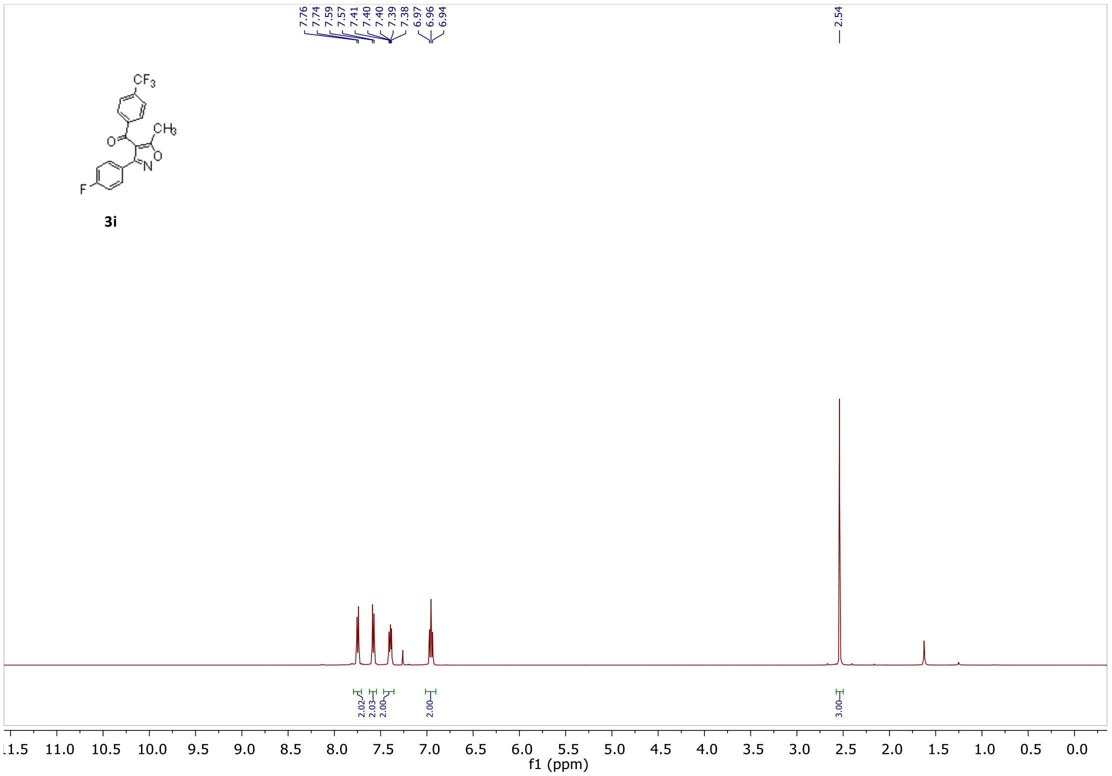

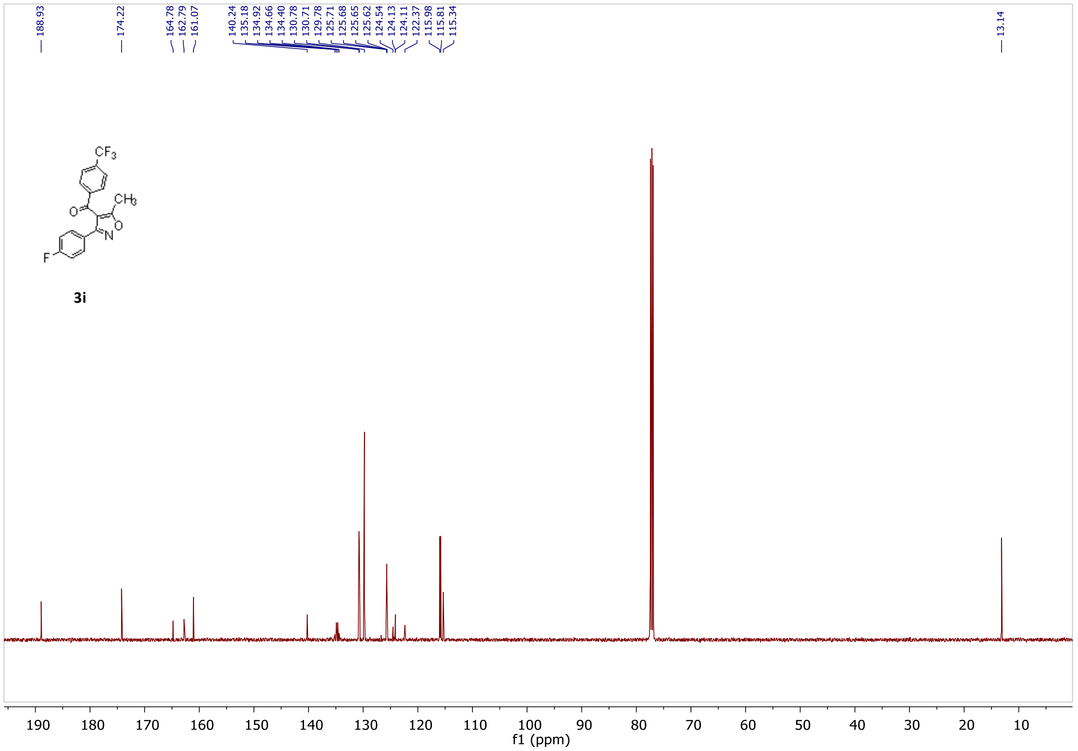

Figure S17: ^{1}H and ^{13}C NMR spectra of compound **3i**.

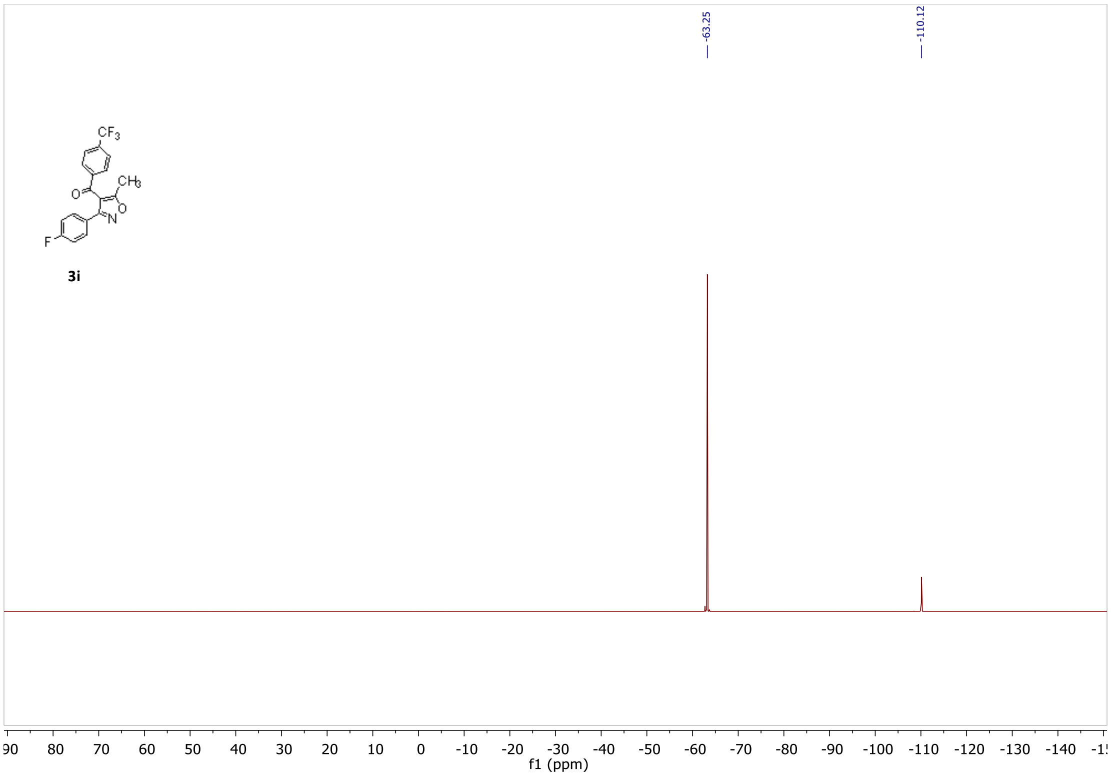

Figure S18: ^{19}F NMR spectrum of compound **3i**.

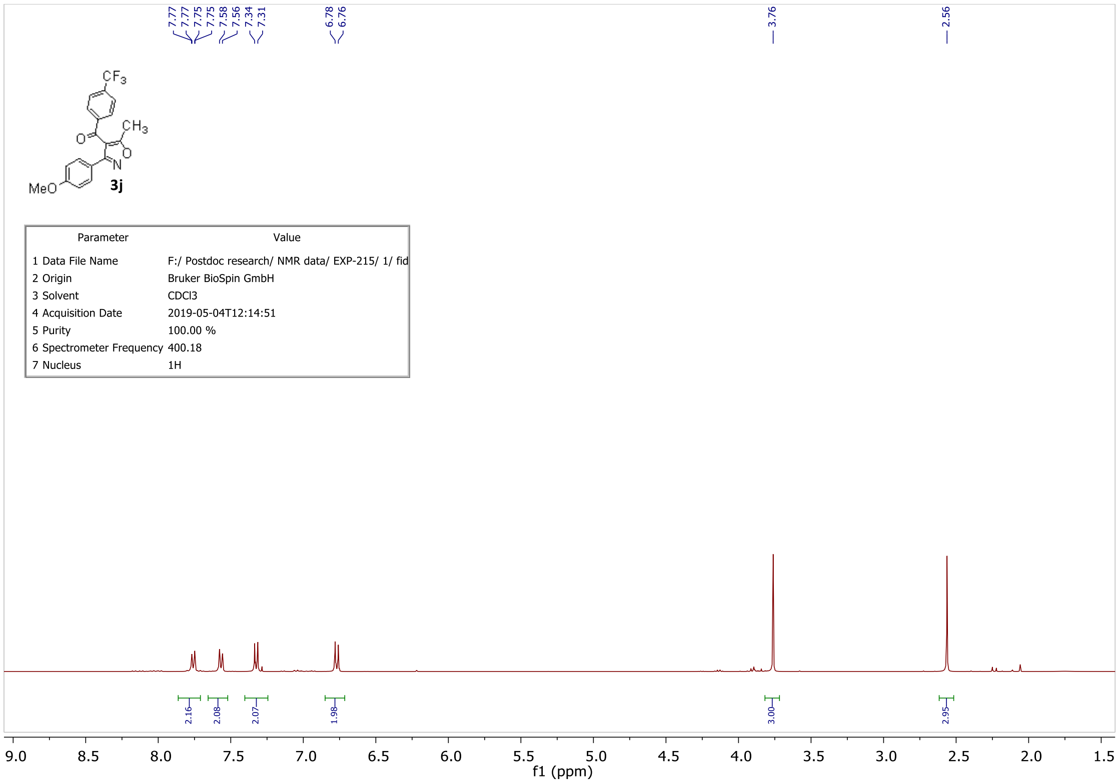

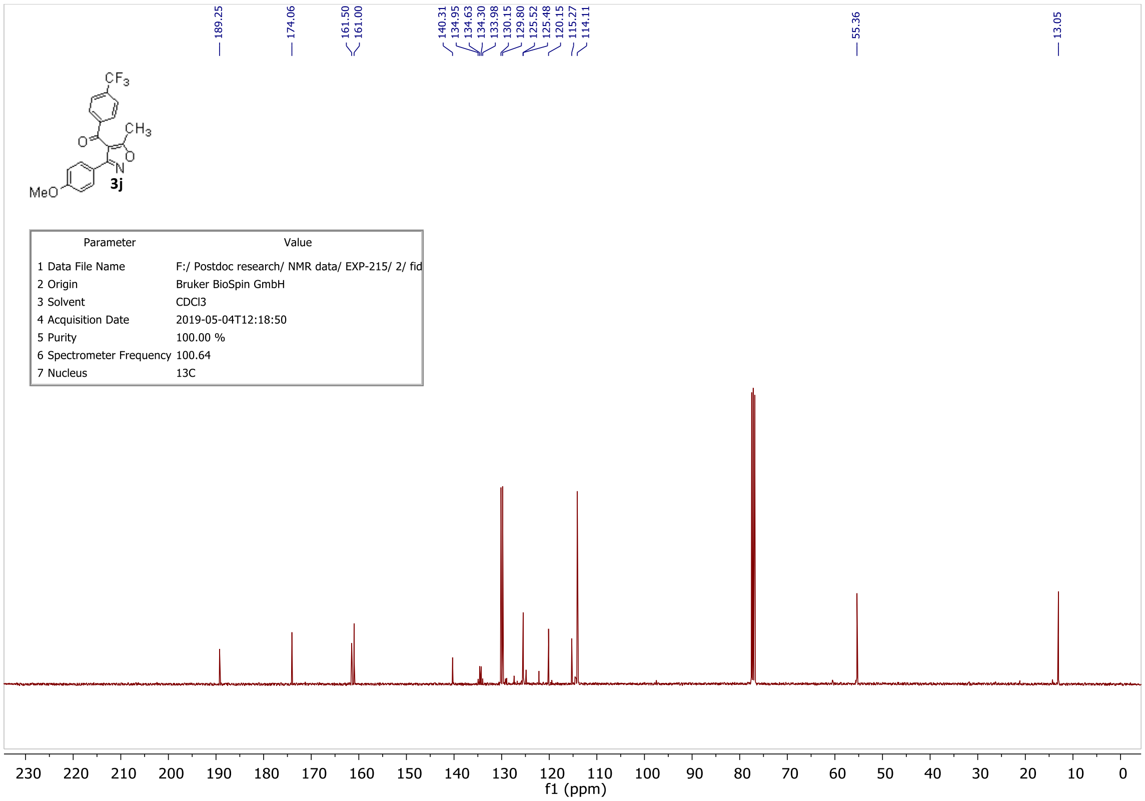

Figure S19: ^{1}H and ^{13}C NMR spectra of compound **3j**.

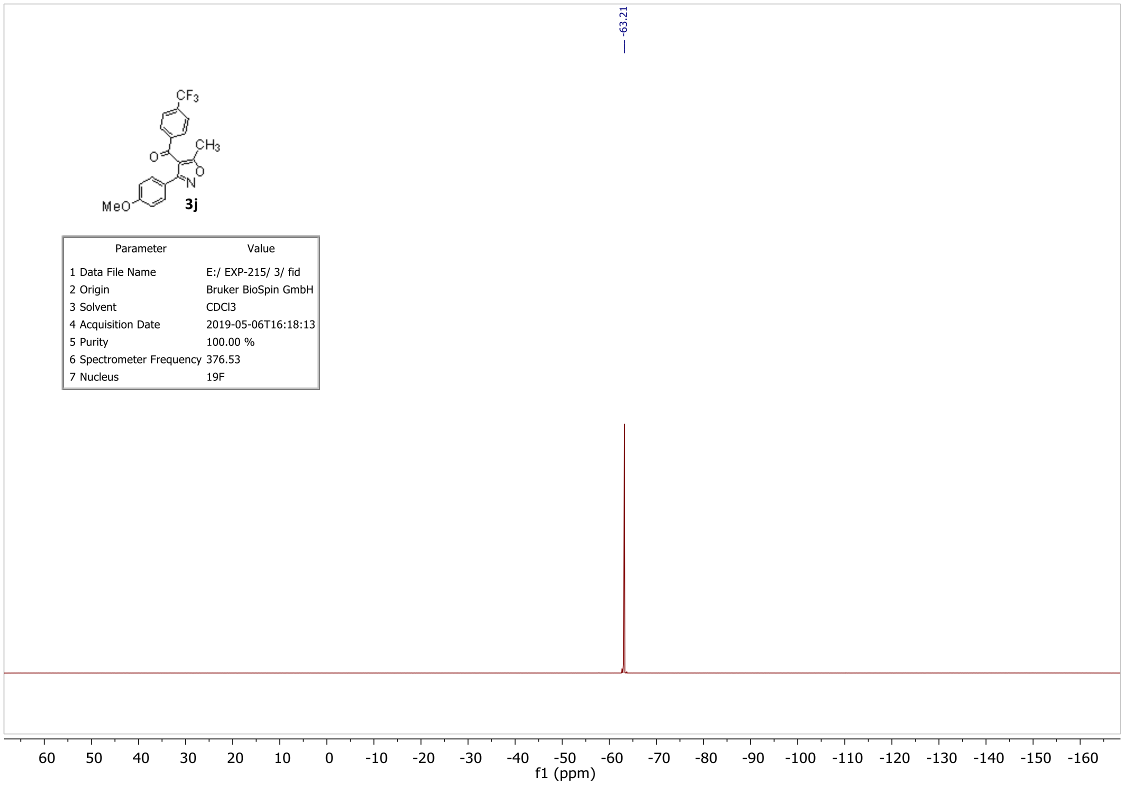

Figure S20: ^{19}F NMR spectrum of compound **3j**.

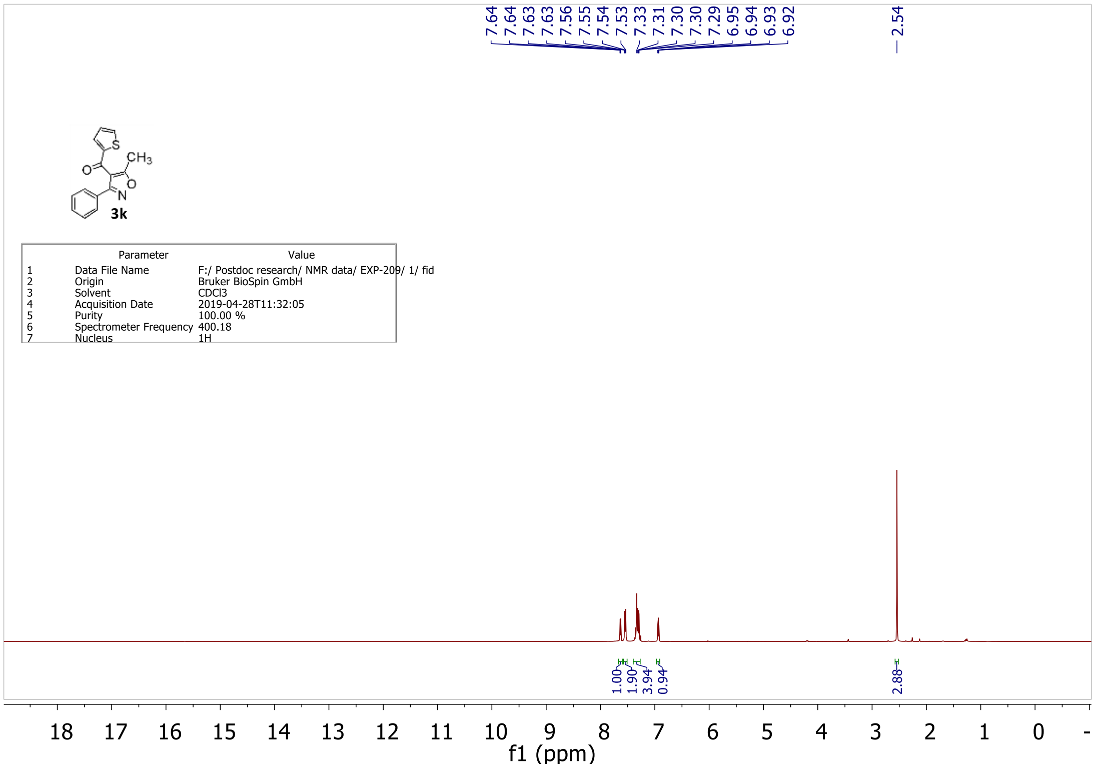

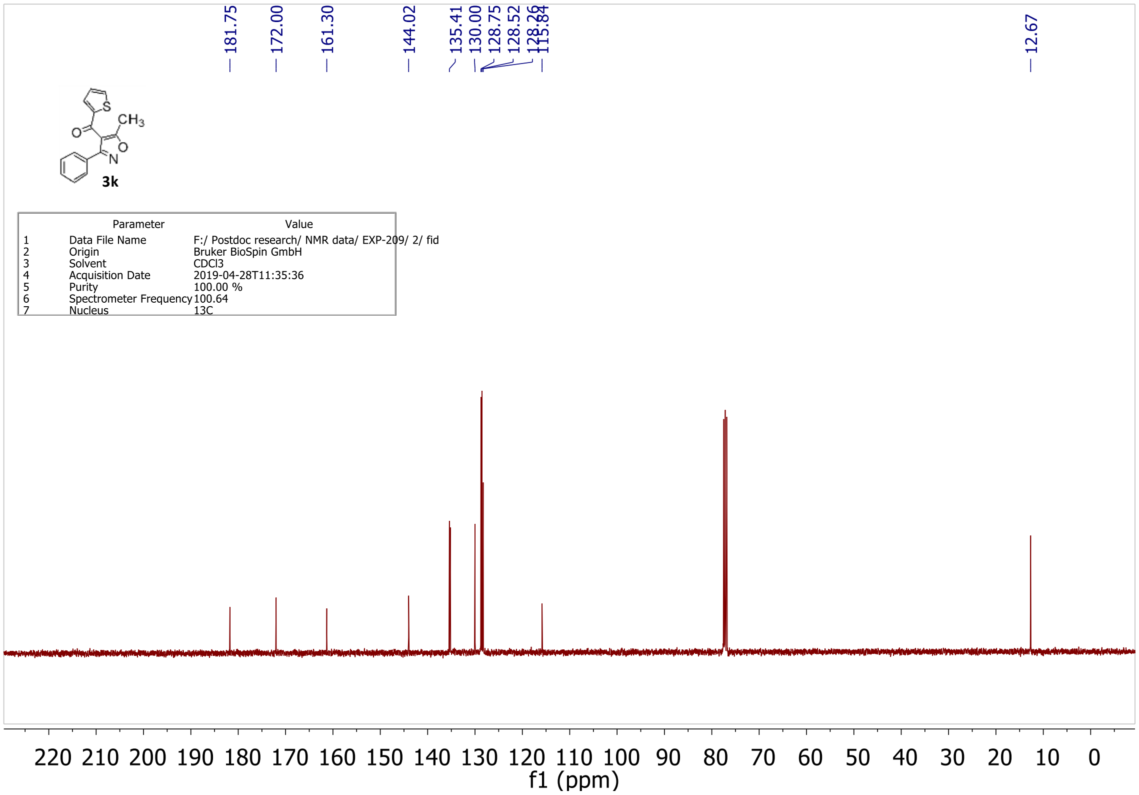

Figure S21: ^{1}H and ^{13}C NMR spectra of compound **3k**.

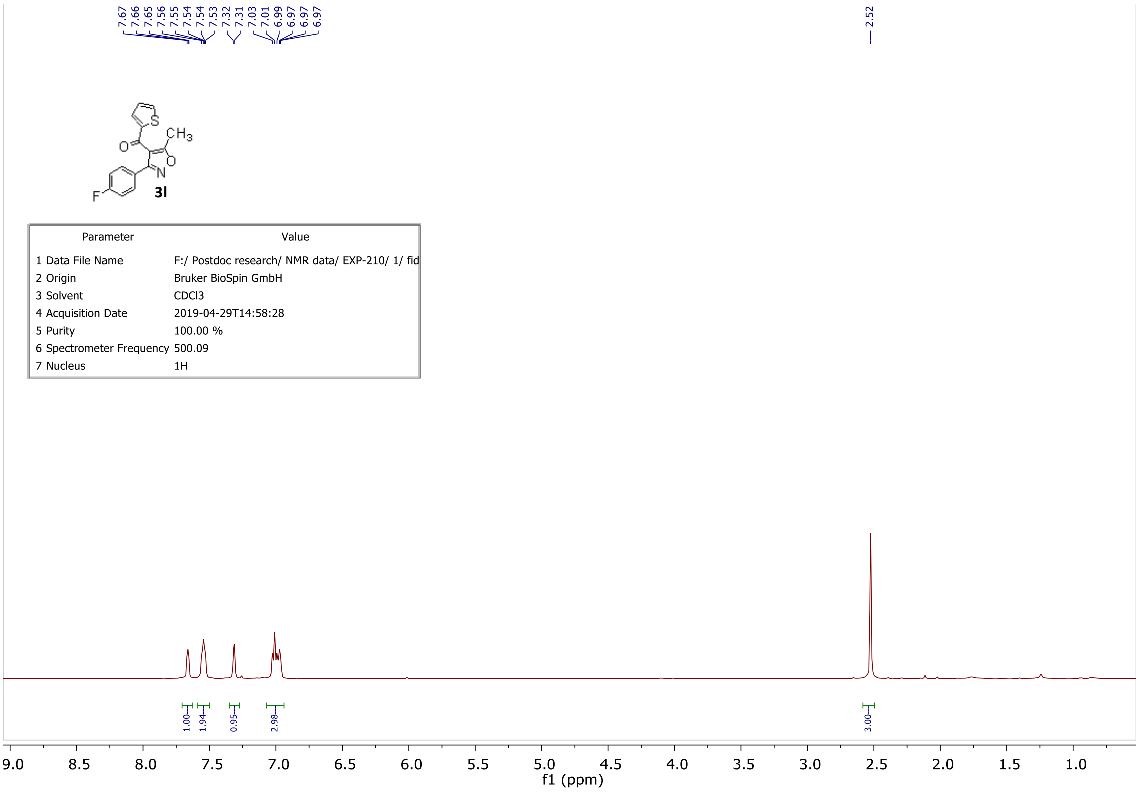

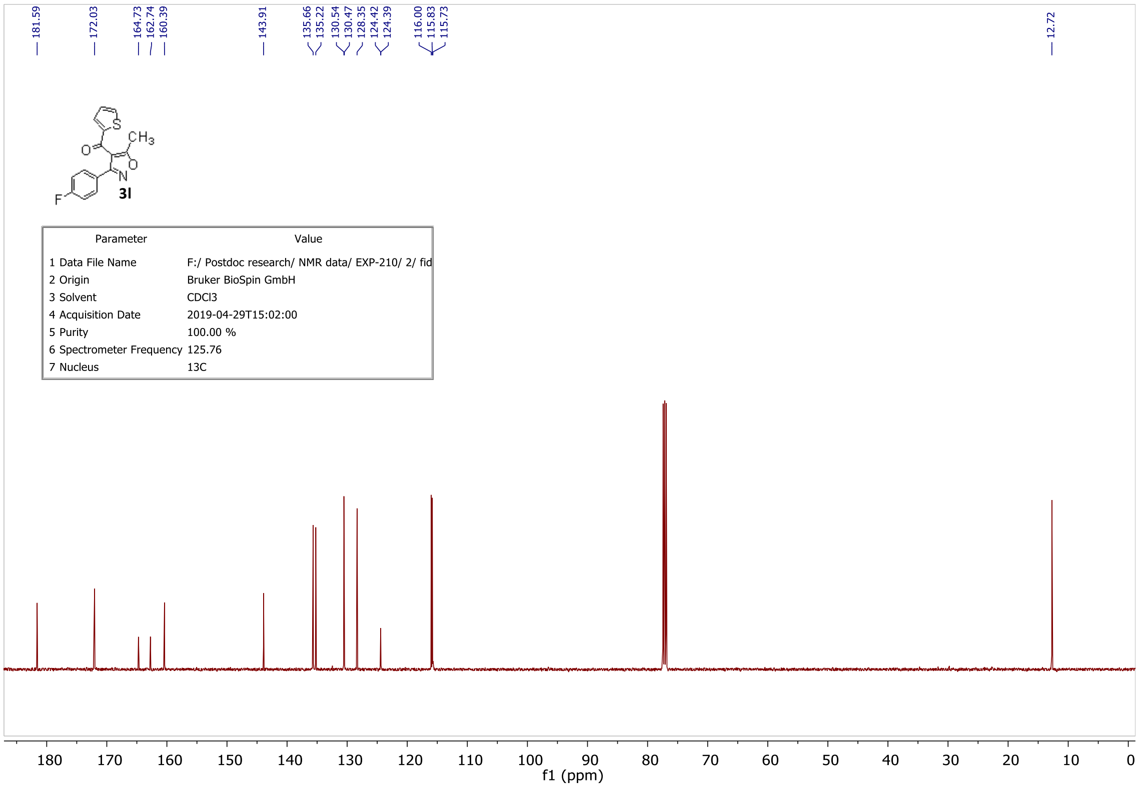

Figure S22: ^{1}H and ^{13}C NMR spectra of compound **3l**.

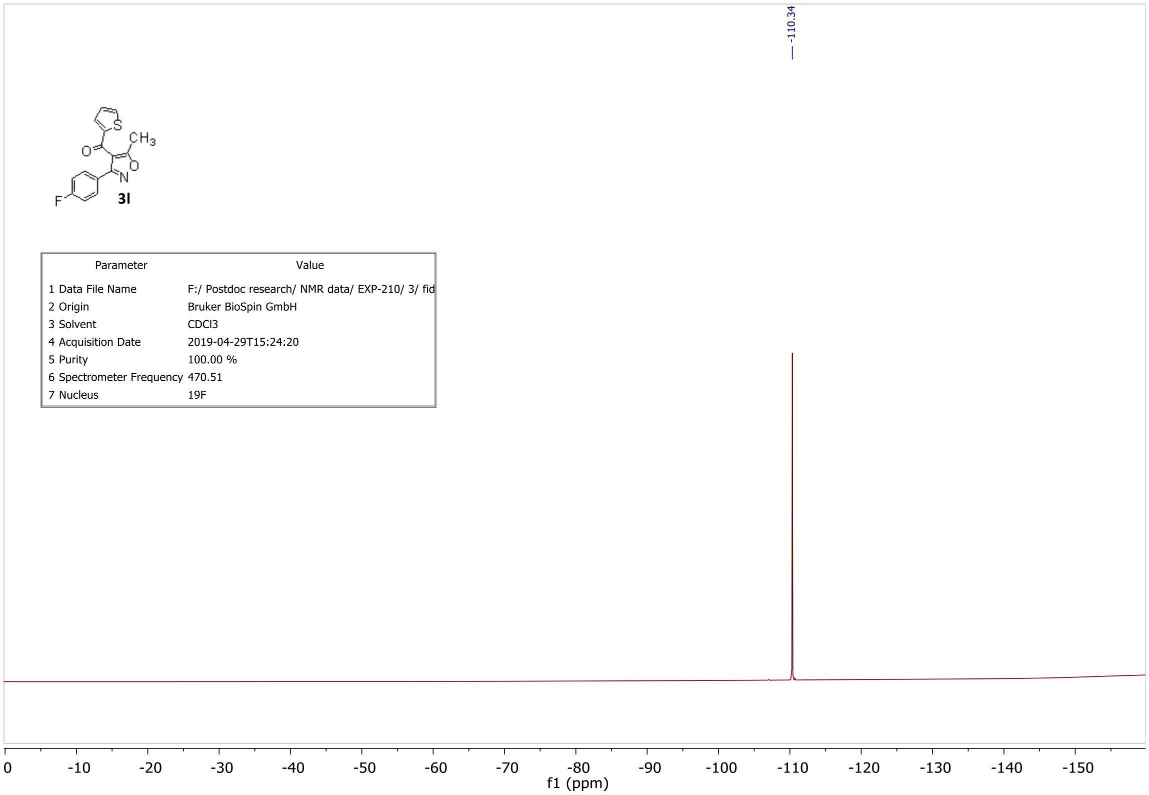

Figure S23: ^{19}F NMR spectrum of compound **3l**.

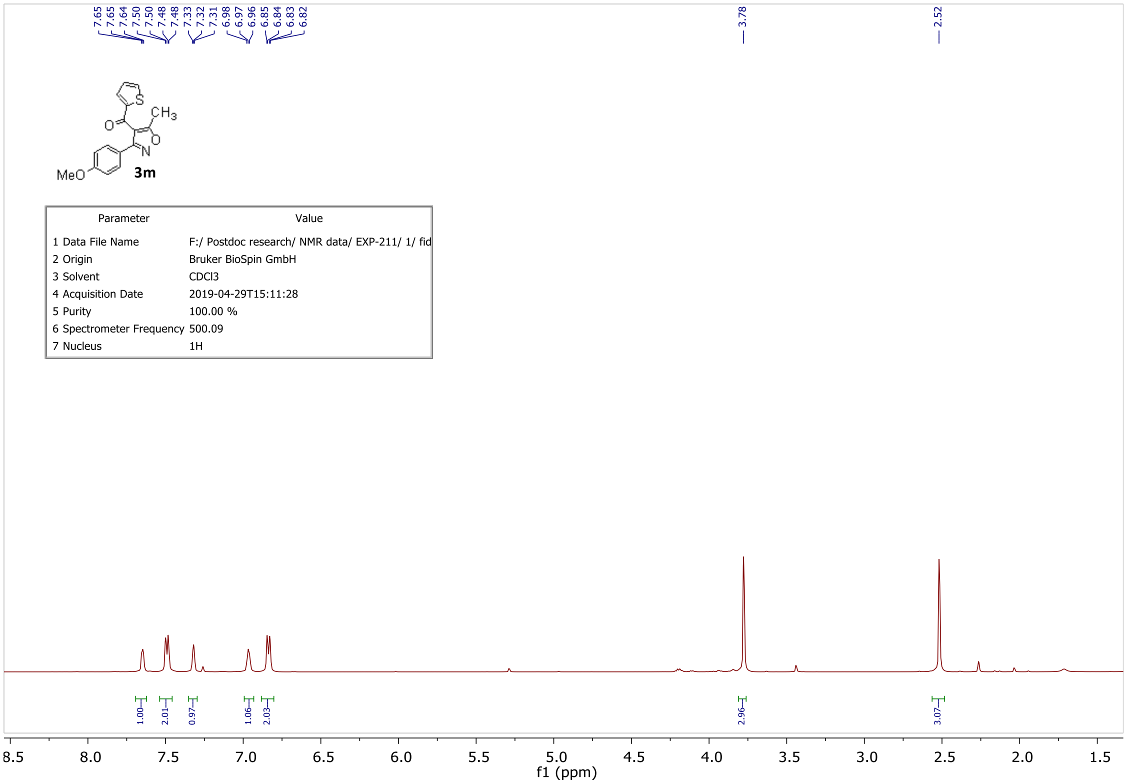

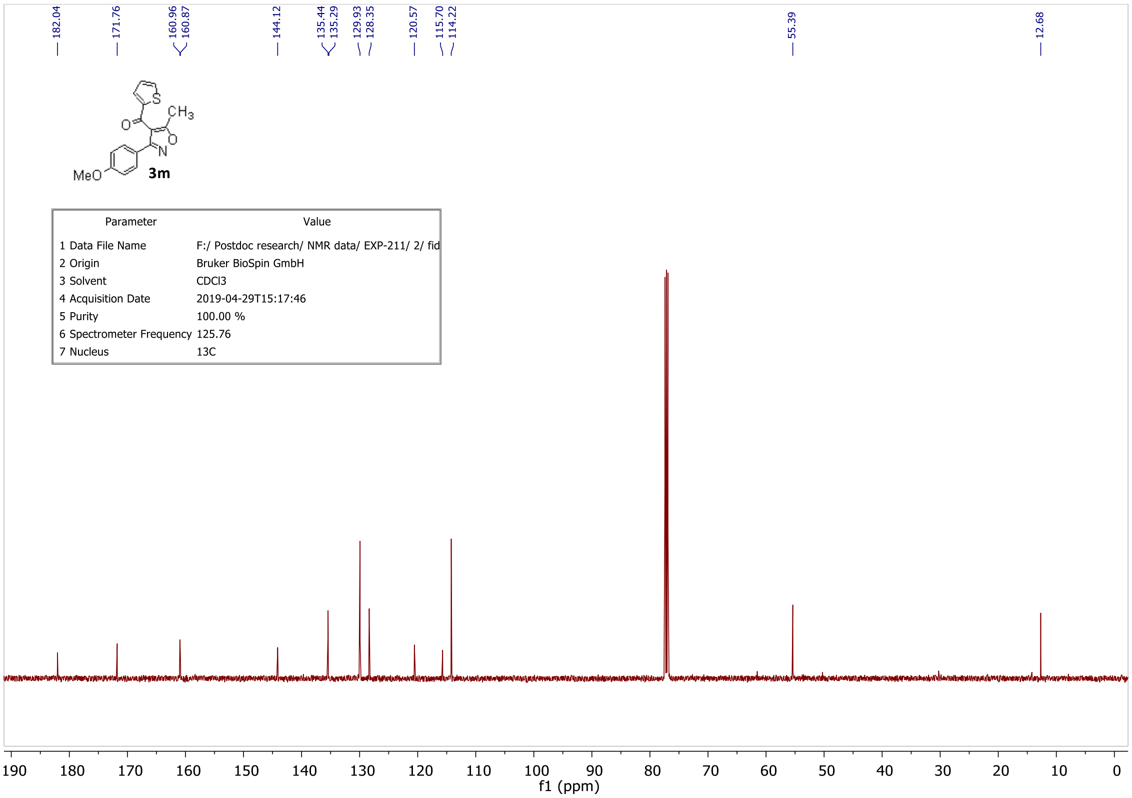

Figure S24: ^{1}H and ^{13}C NMR spectra of compound **3m**.

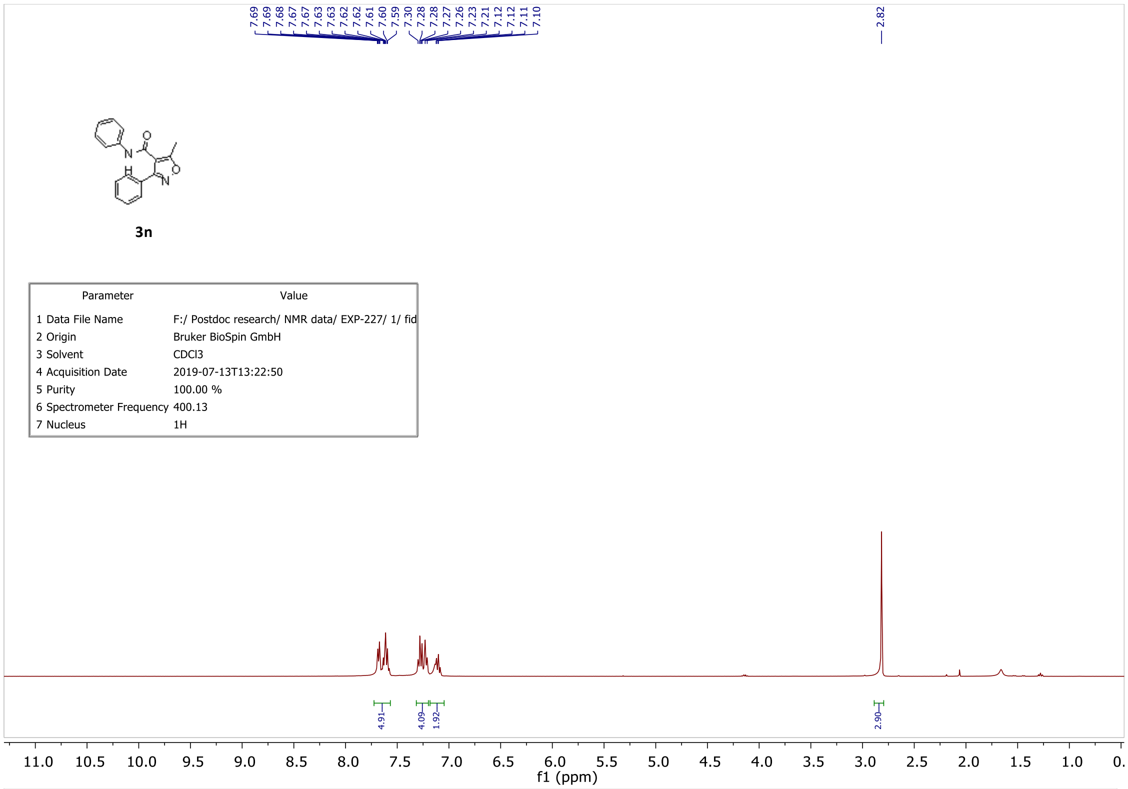

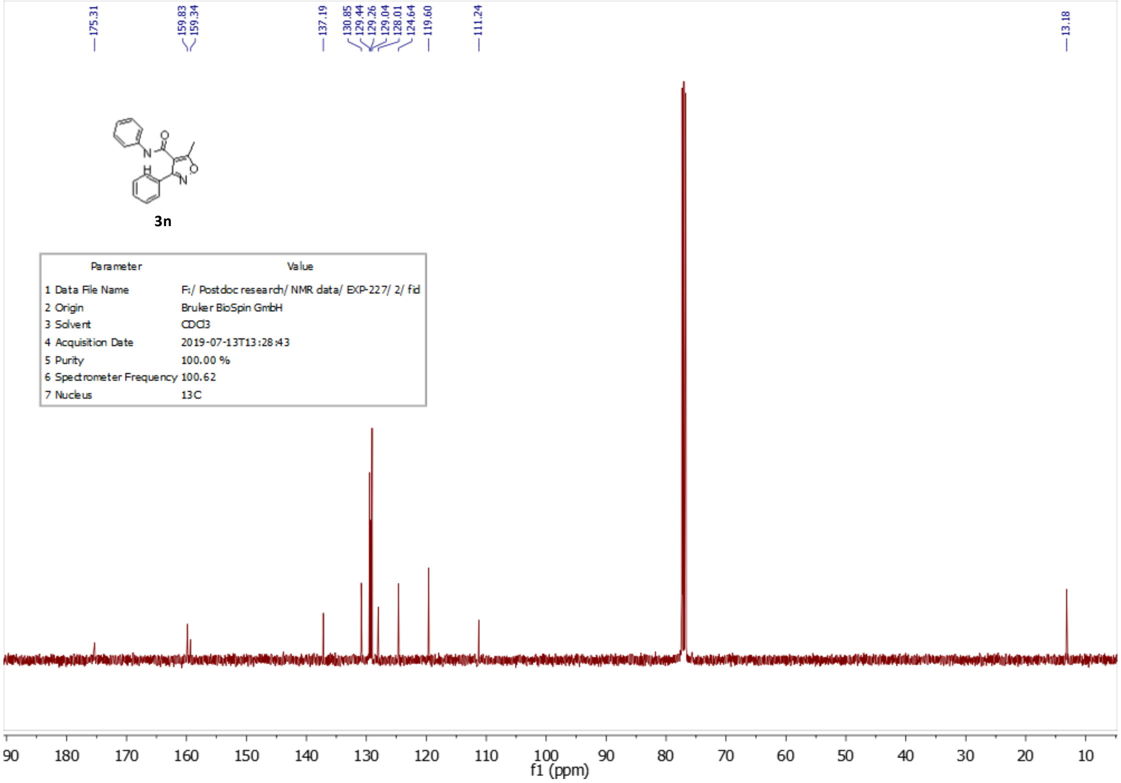

Figure S25: ^{1}H and ^{13}C NMR spectra of compound **3n**.

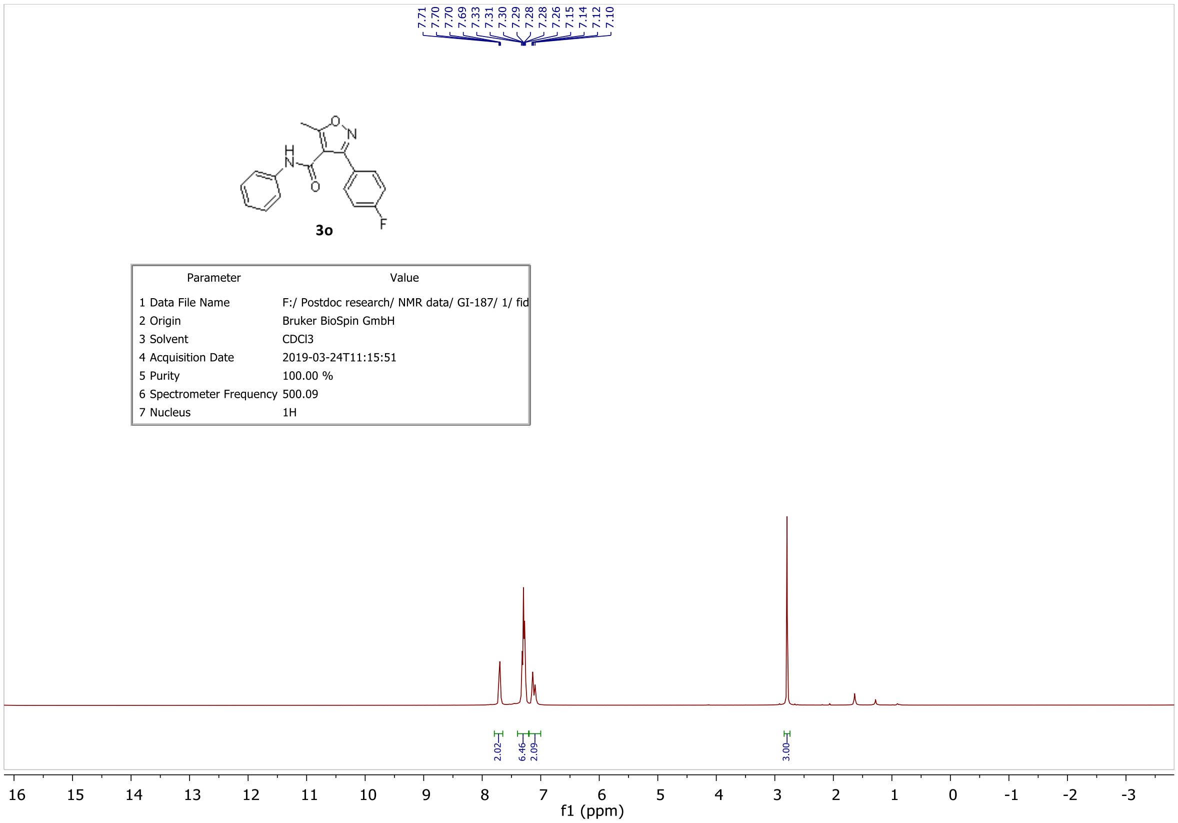

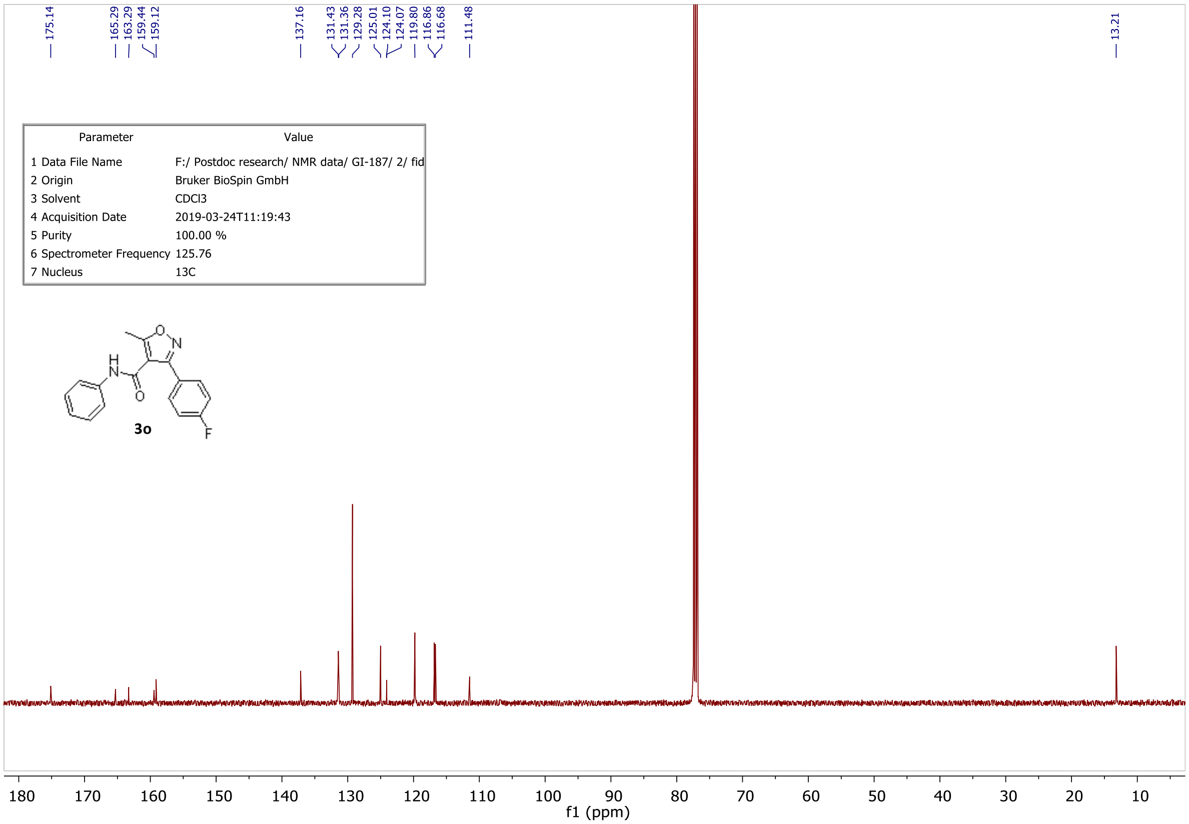

Figure S26: ^{1}H and ^{13}C NMR spectra of compound **3o**.

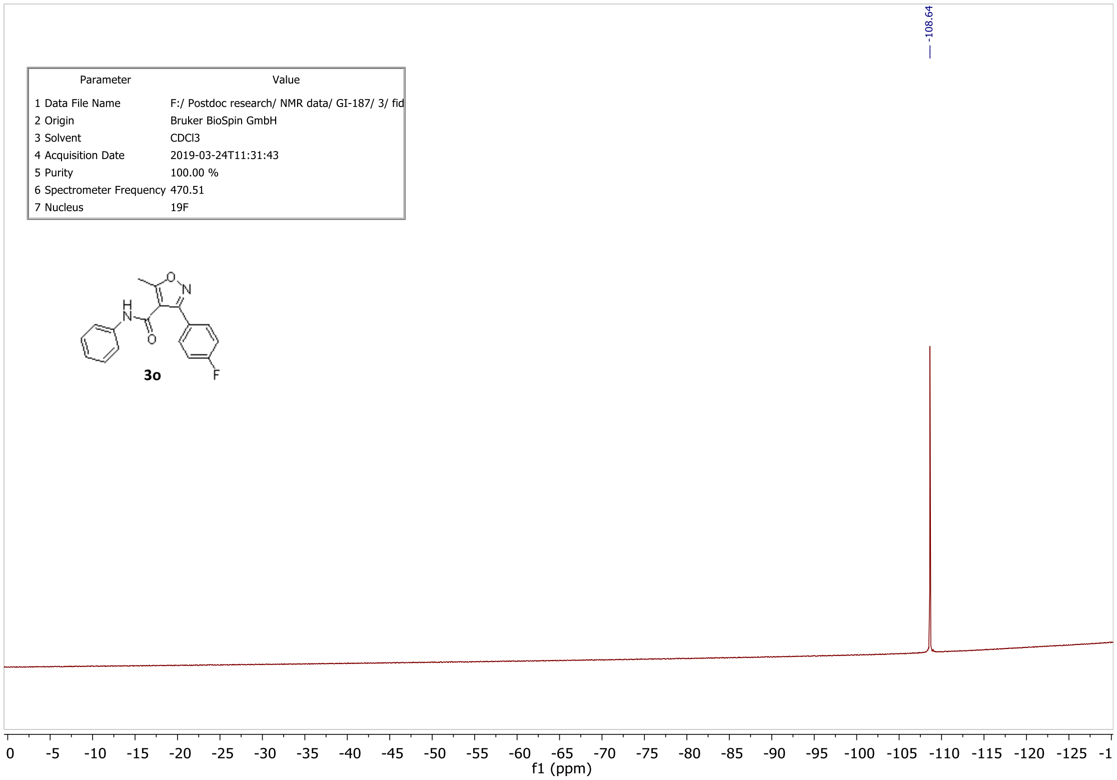

Figure S27: ^{19}F NMR spectrum of compound **3o**.

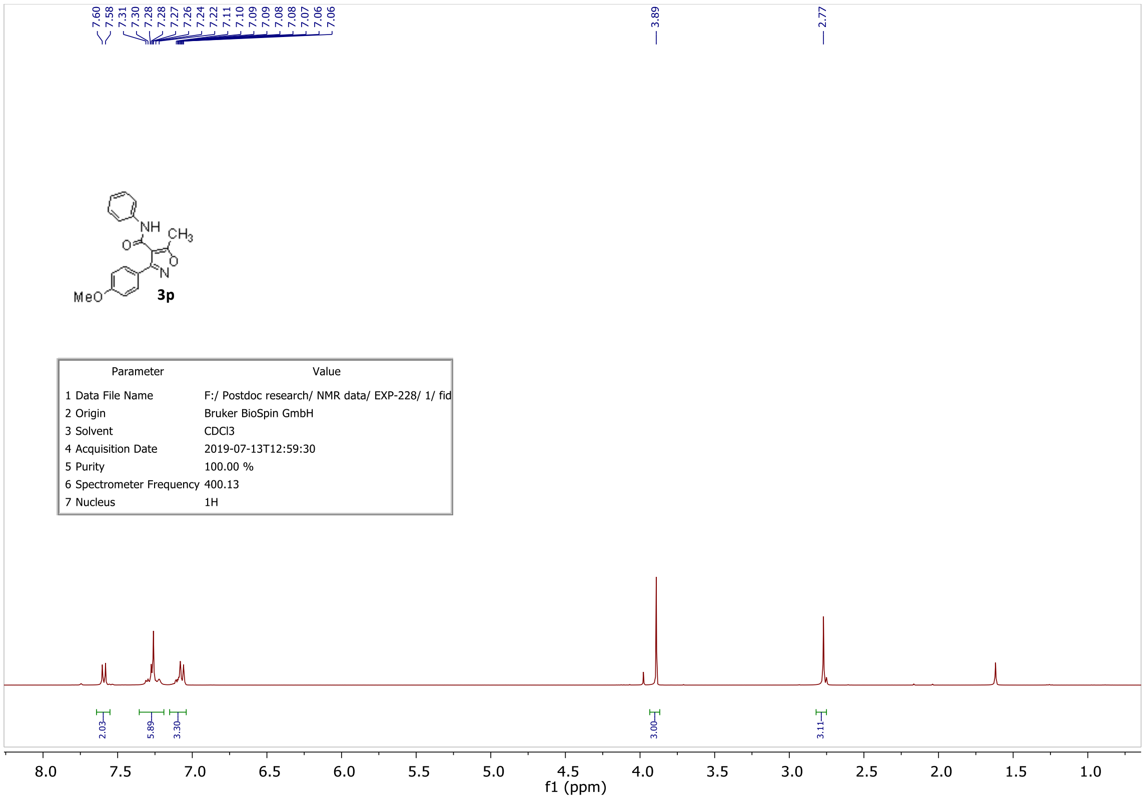

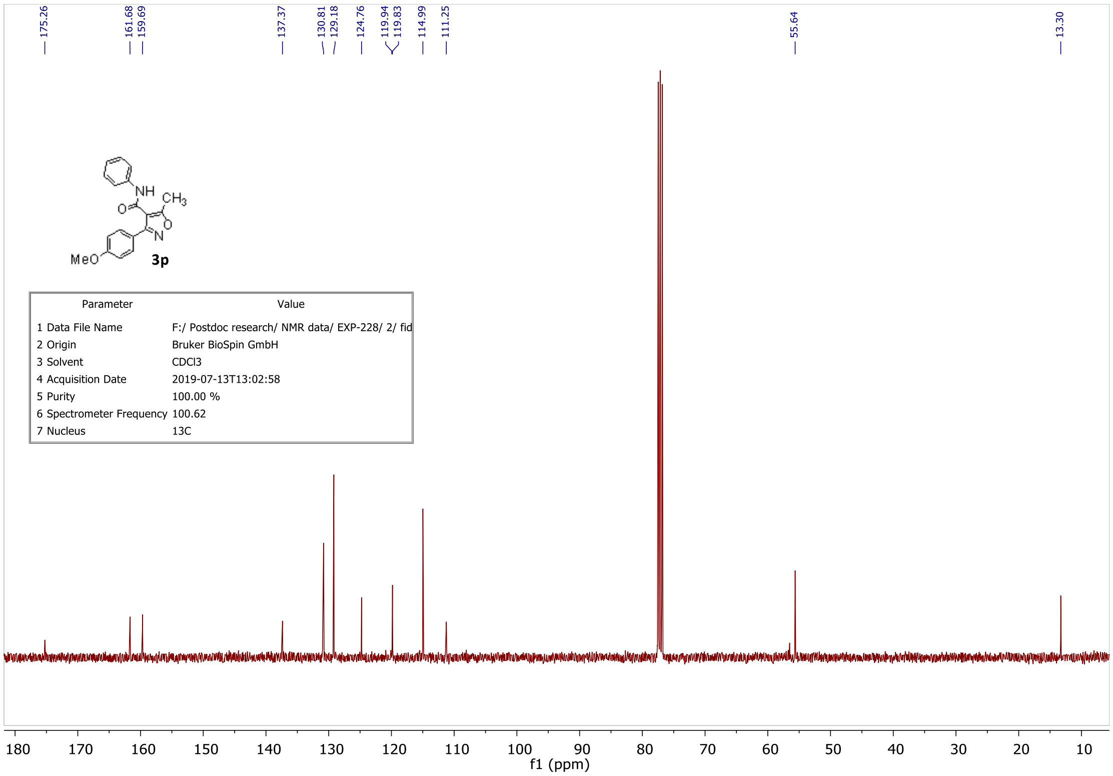

Figure S28: ^{1}H and ^{13}C NMR spectra of compound **3p**.

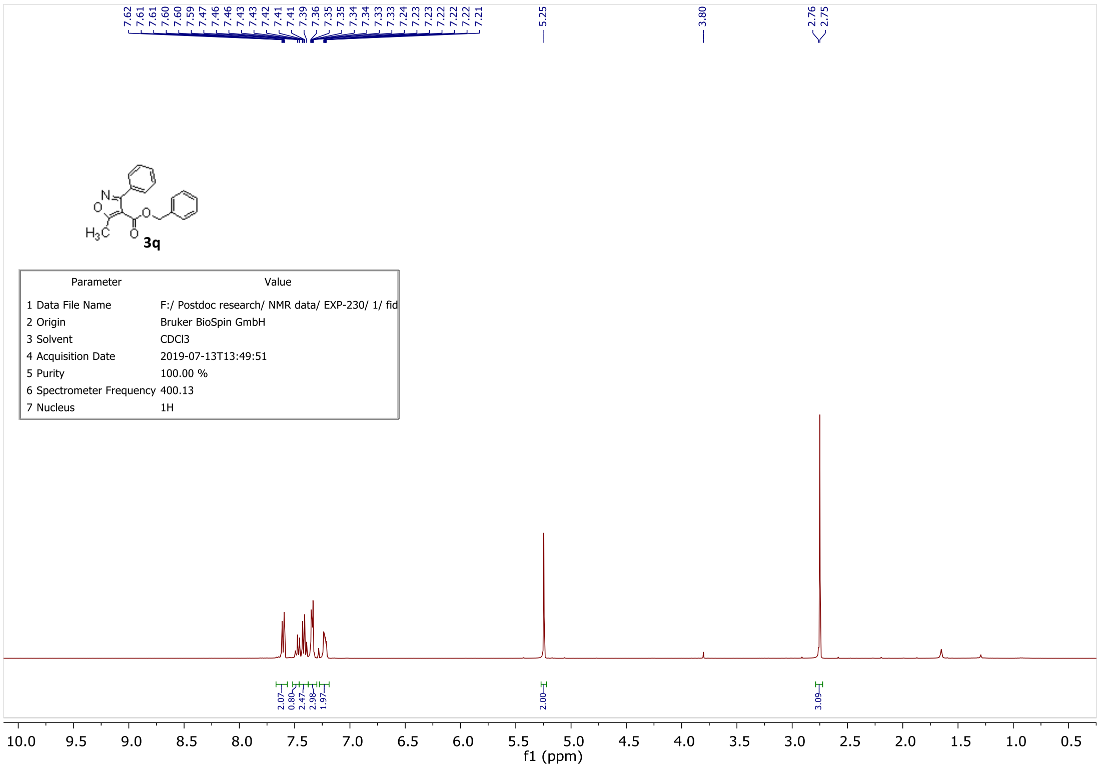

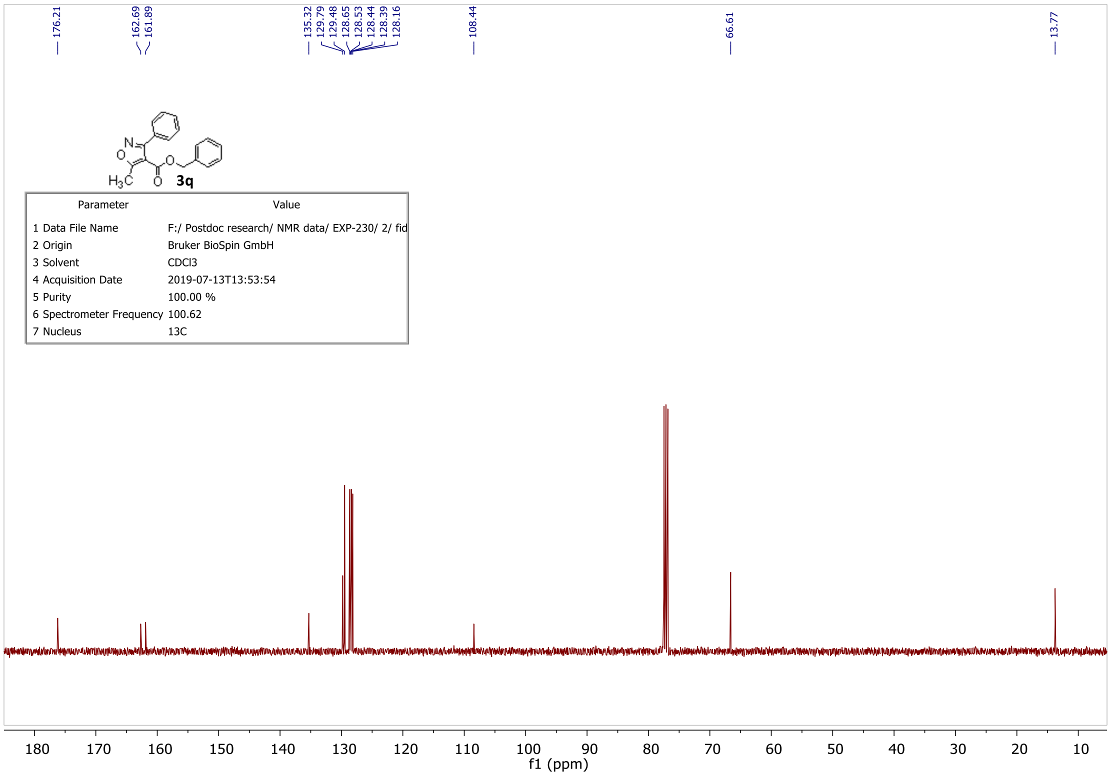

Figure S29: ^{1}H and ^{13}C NMR spectra of compound **3q**.

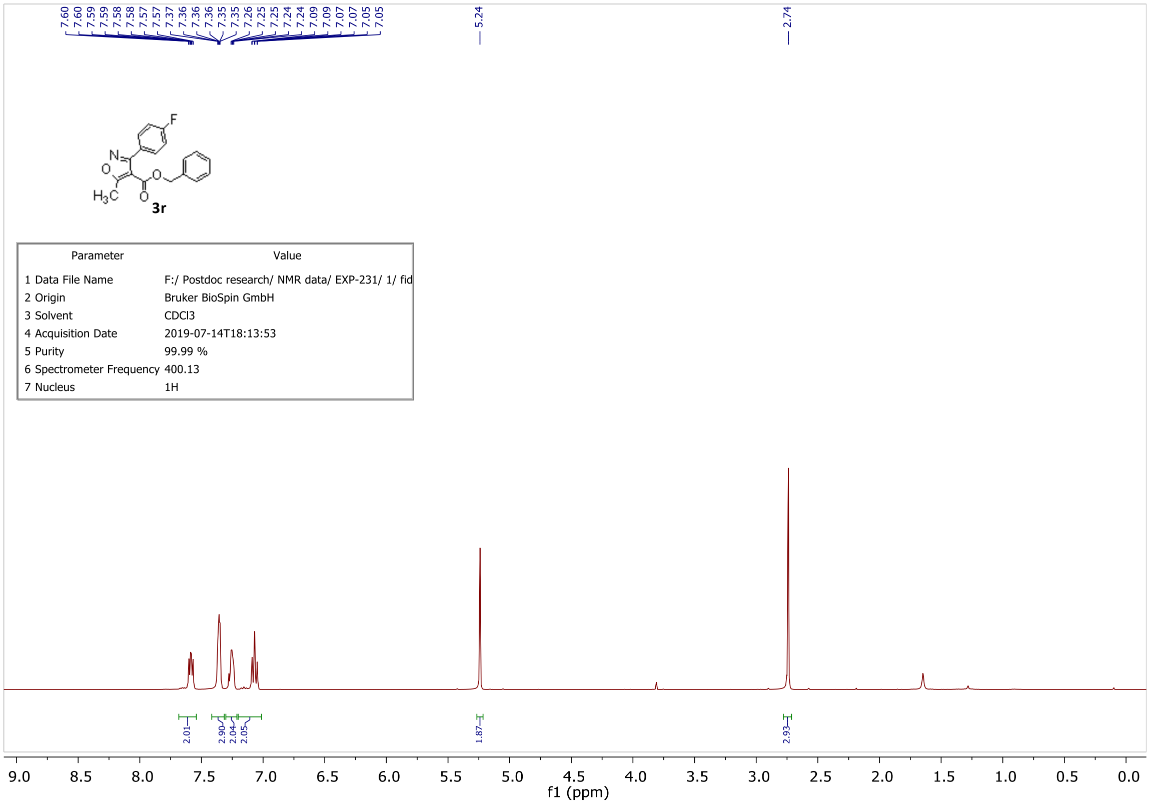

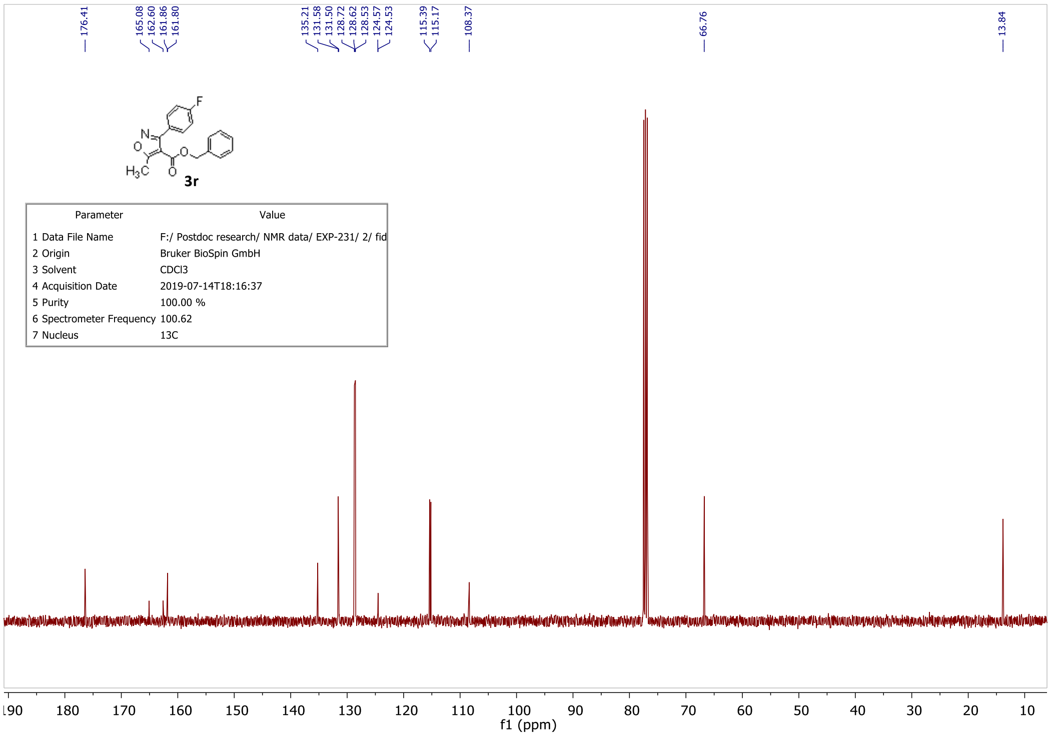

Figure S30: ^{1}H and ^{13}C NMR spectra of compound **3r**.

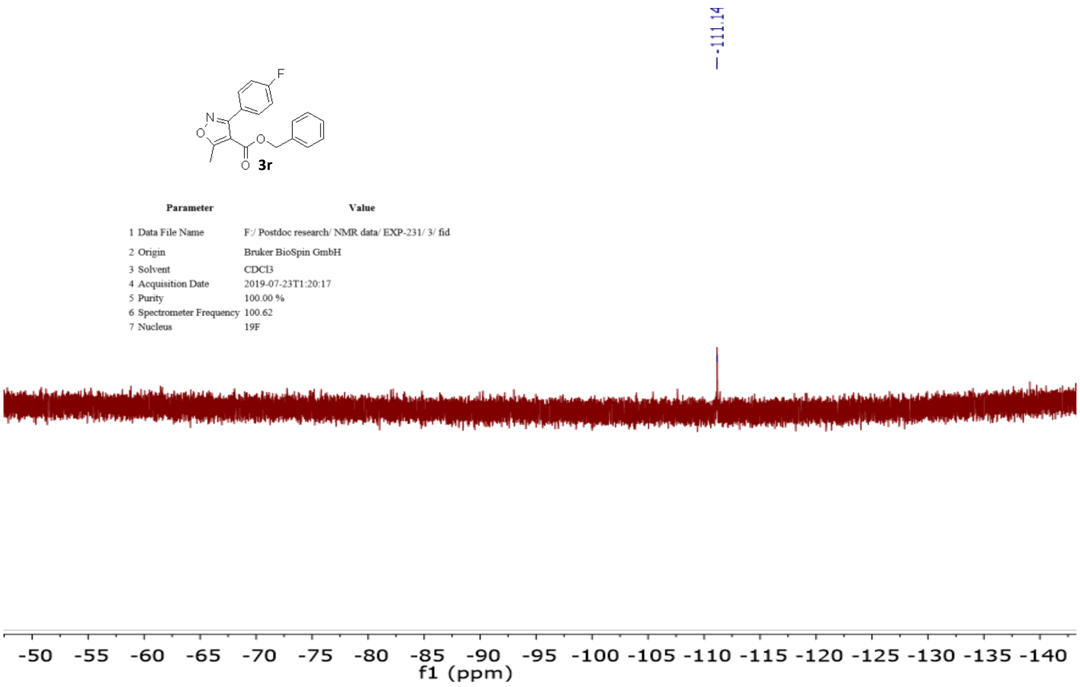

Figure S31: ^{19}F NMR spectrum of compound **3r**.

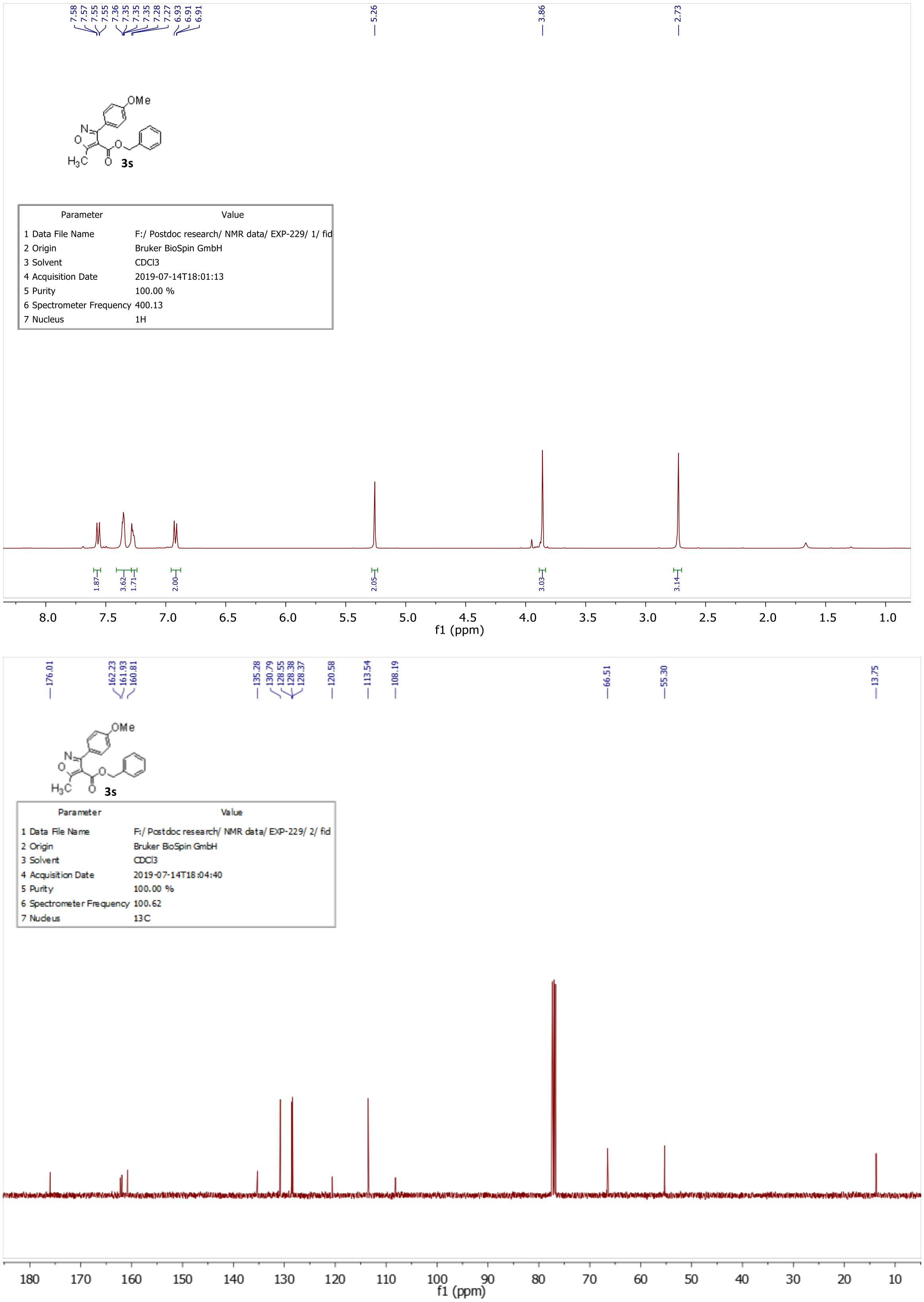

Figure S32: ^{1}H and ^{13}C NMR spectra of compound **3s**.

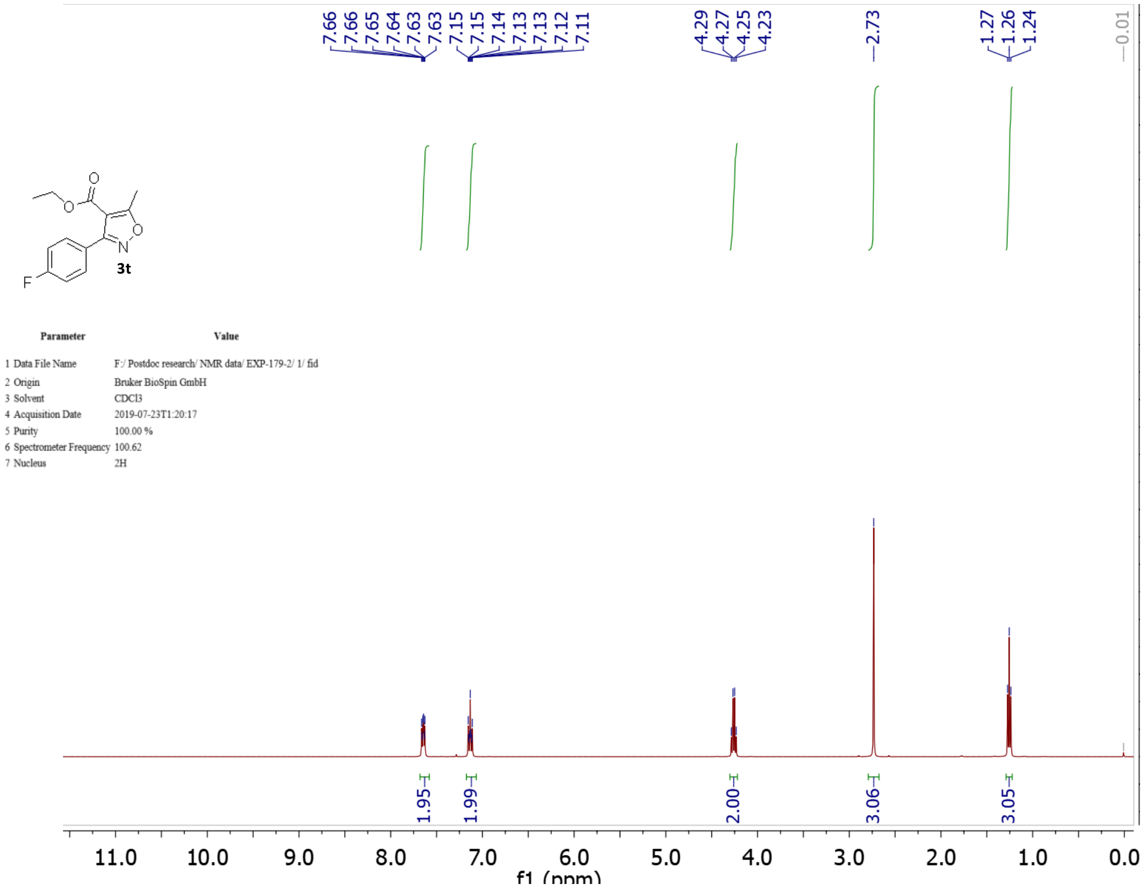

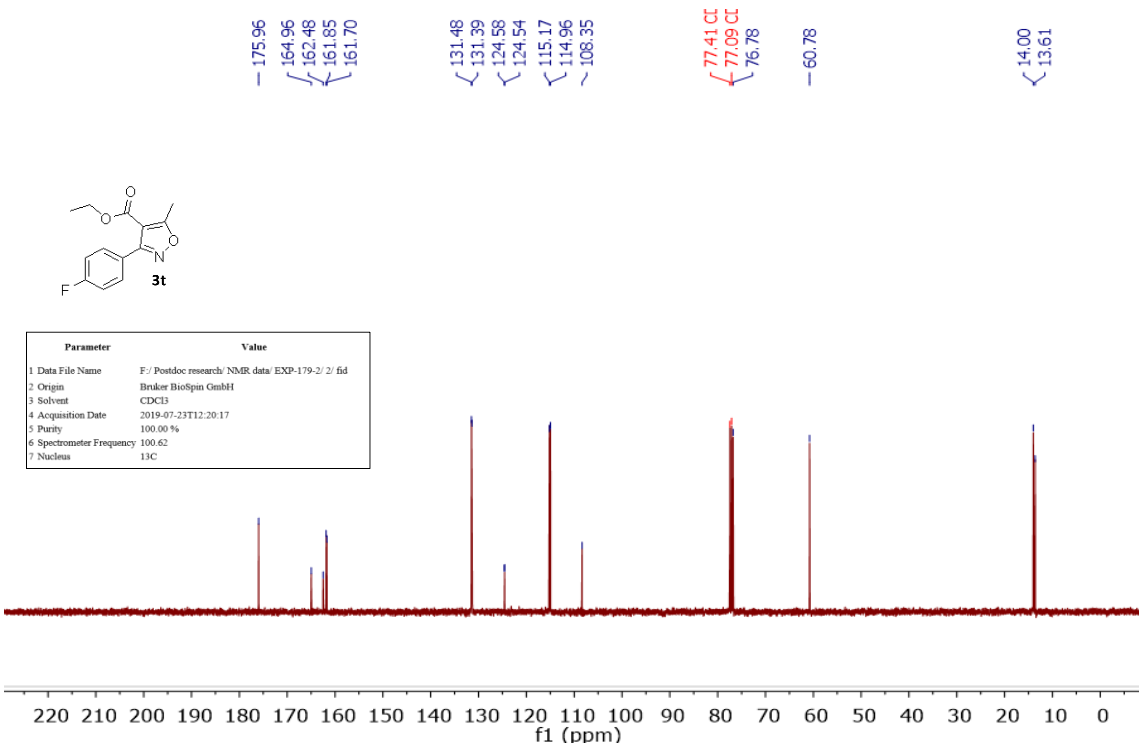

Figure S33: ^{1}H and ^{13}C NMR spectra of compound **3t**.

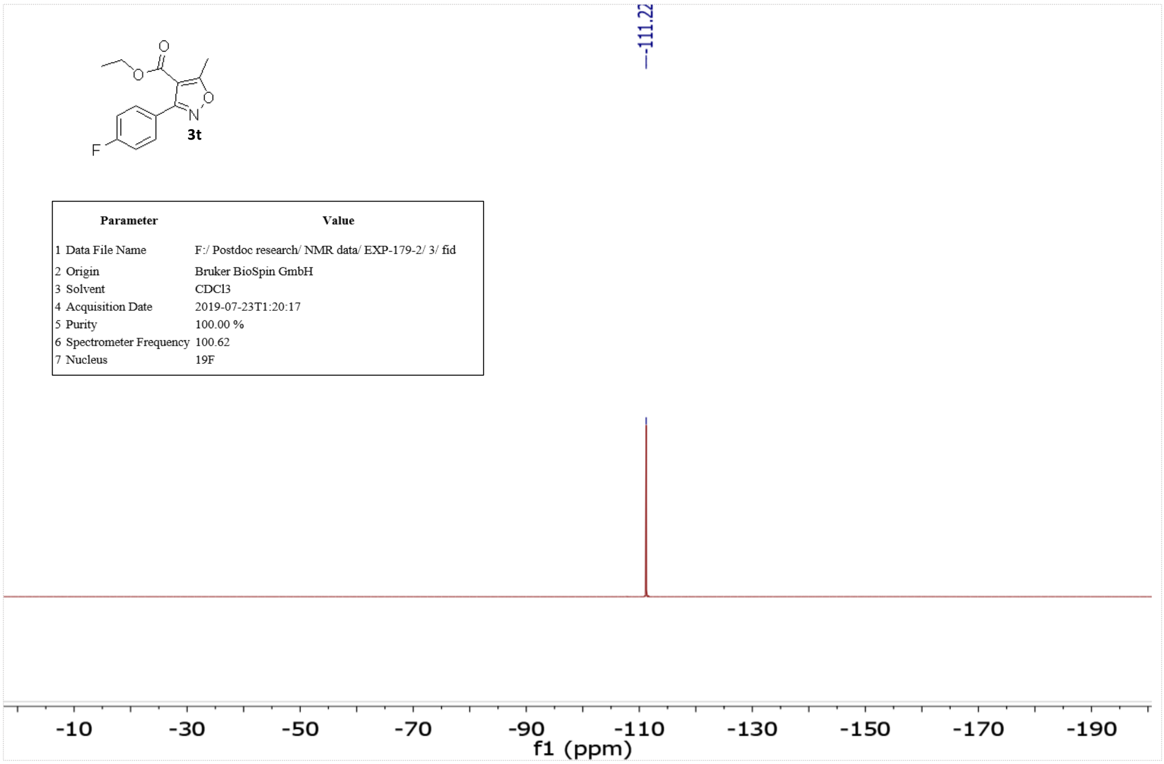

Figure S34: ^{19}F NMR spectrum of compound **3t**.

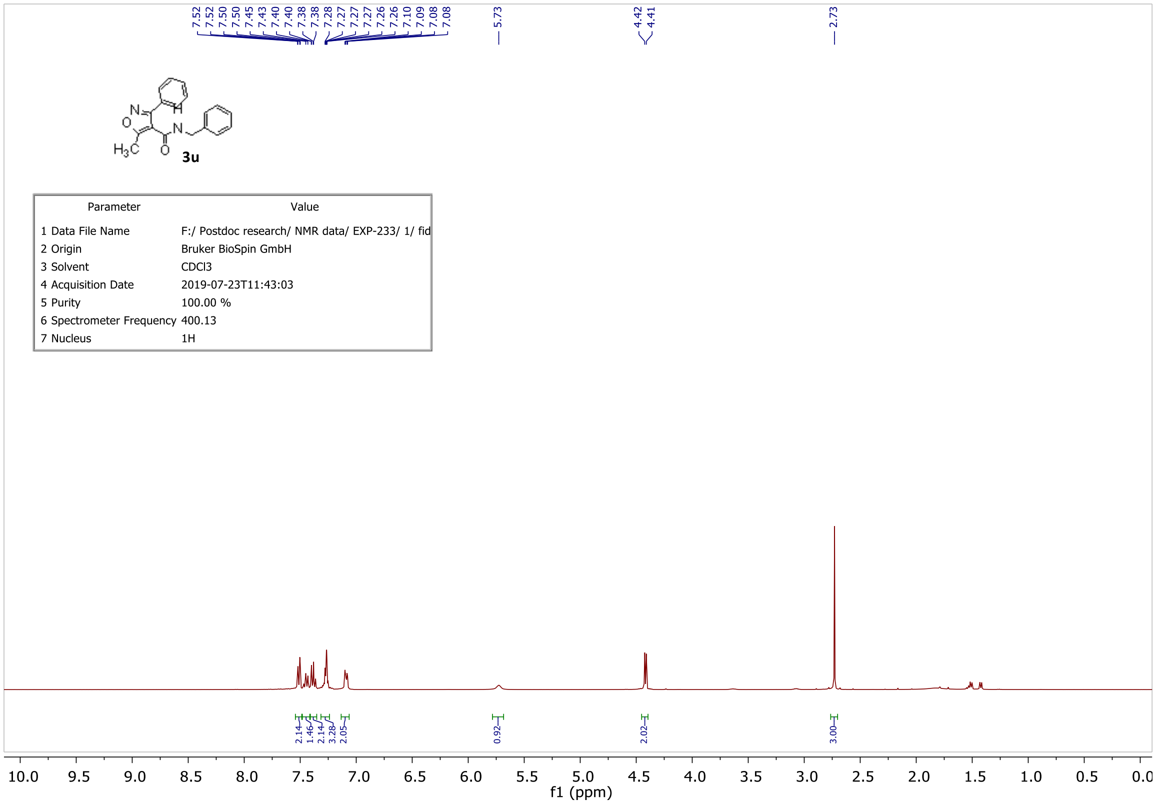

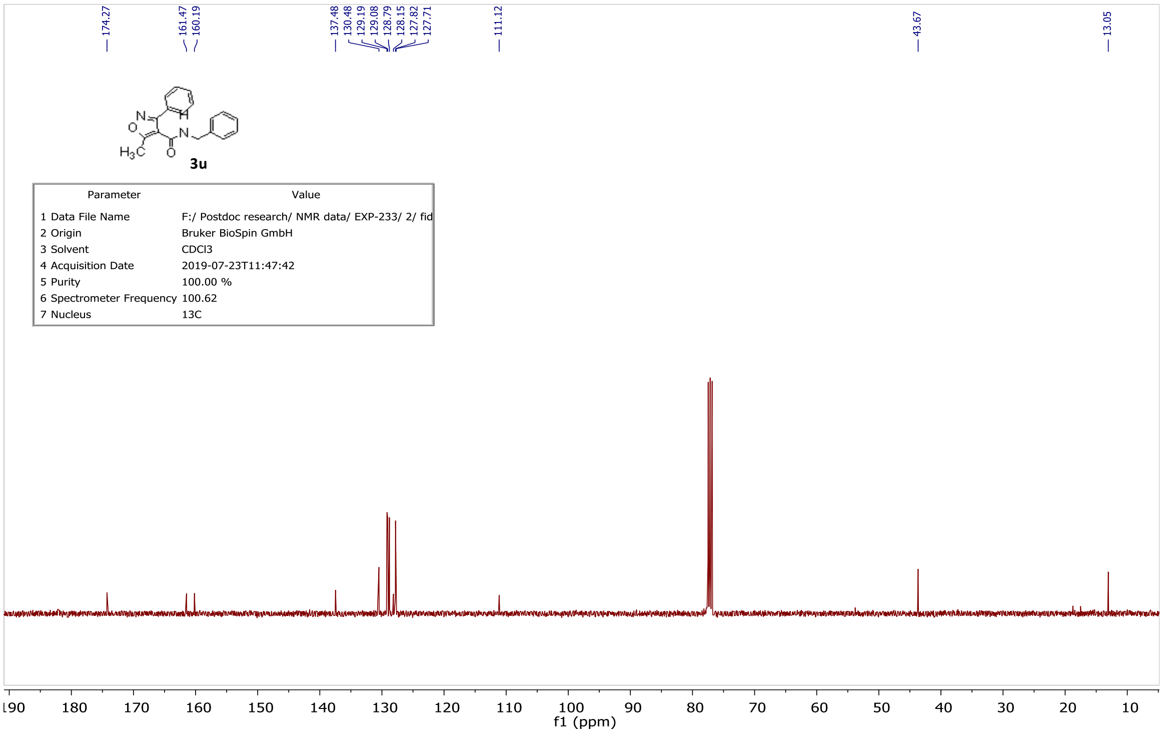

Figure S35: ^{1}H and ^{13}C NMR spectra of compound **3u**.

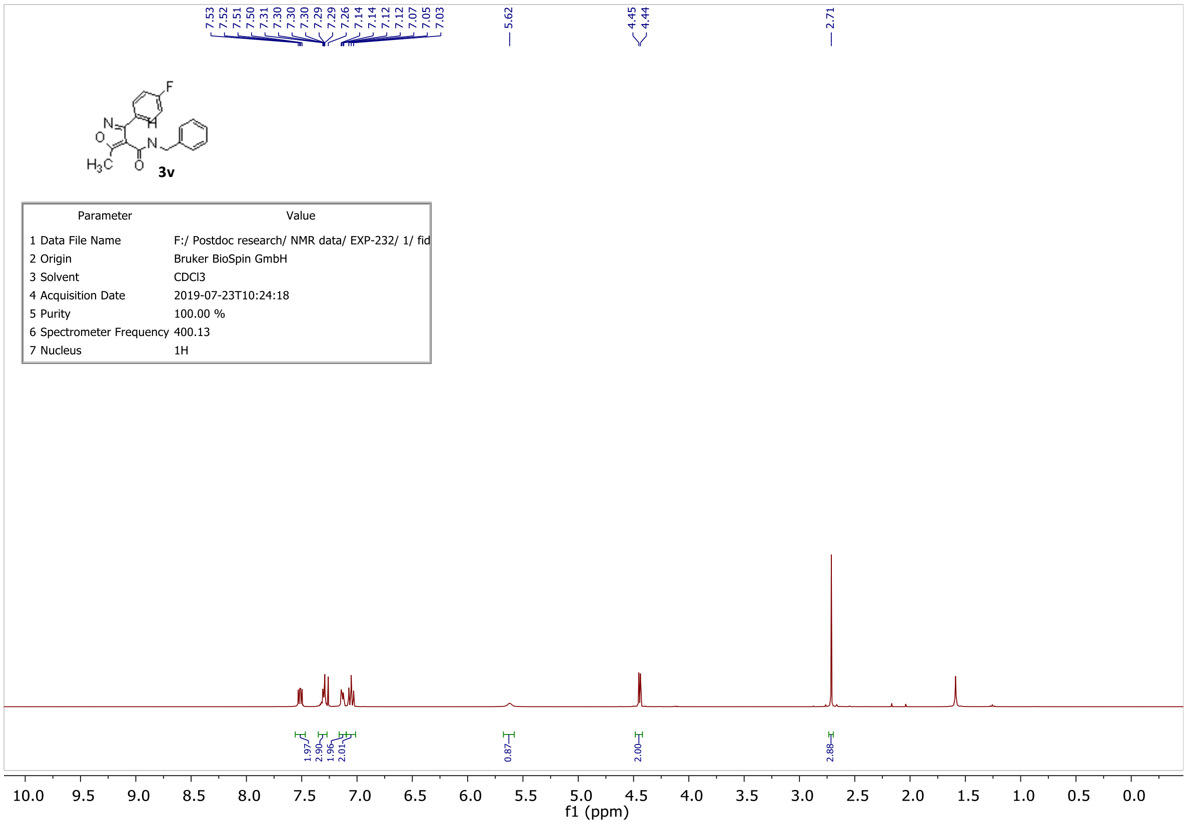

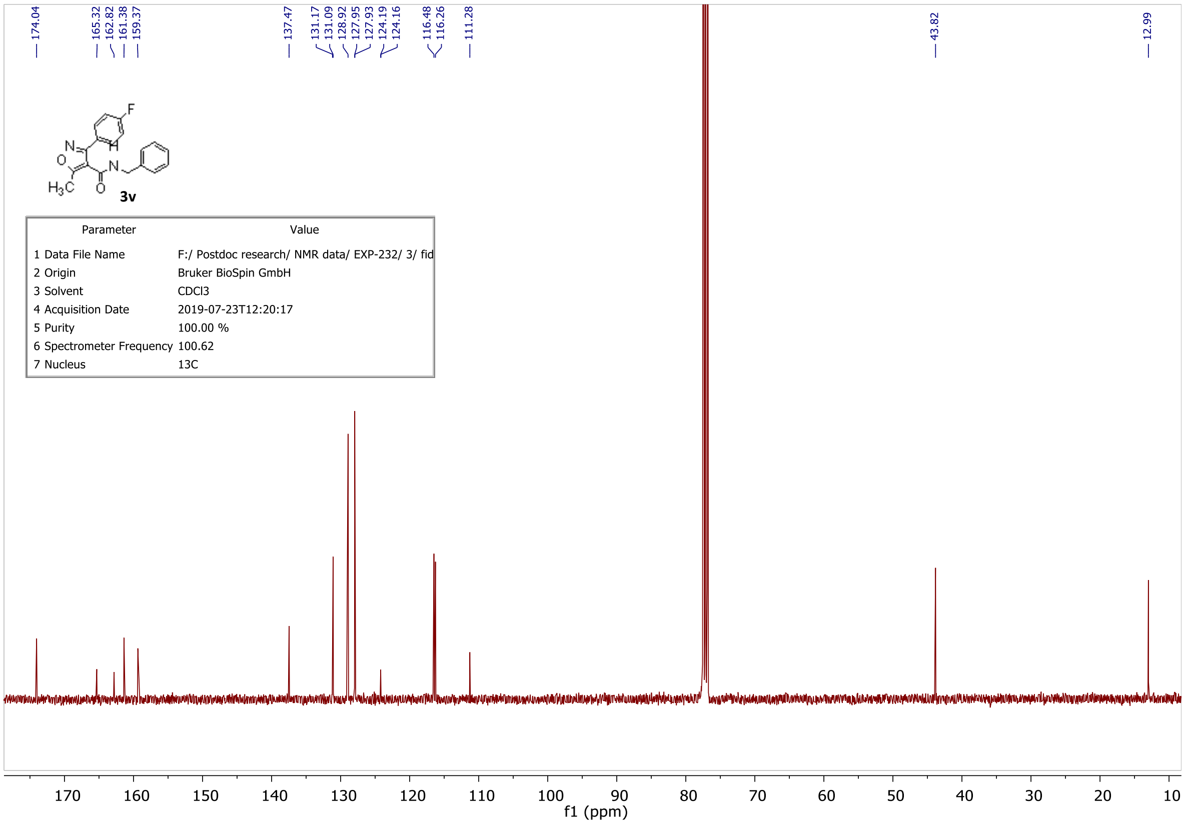

Figure S36: ^{1}H and ^{13}C NMR spectra of compound **3v**.

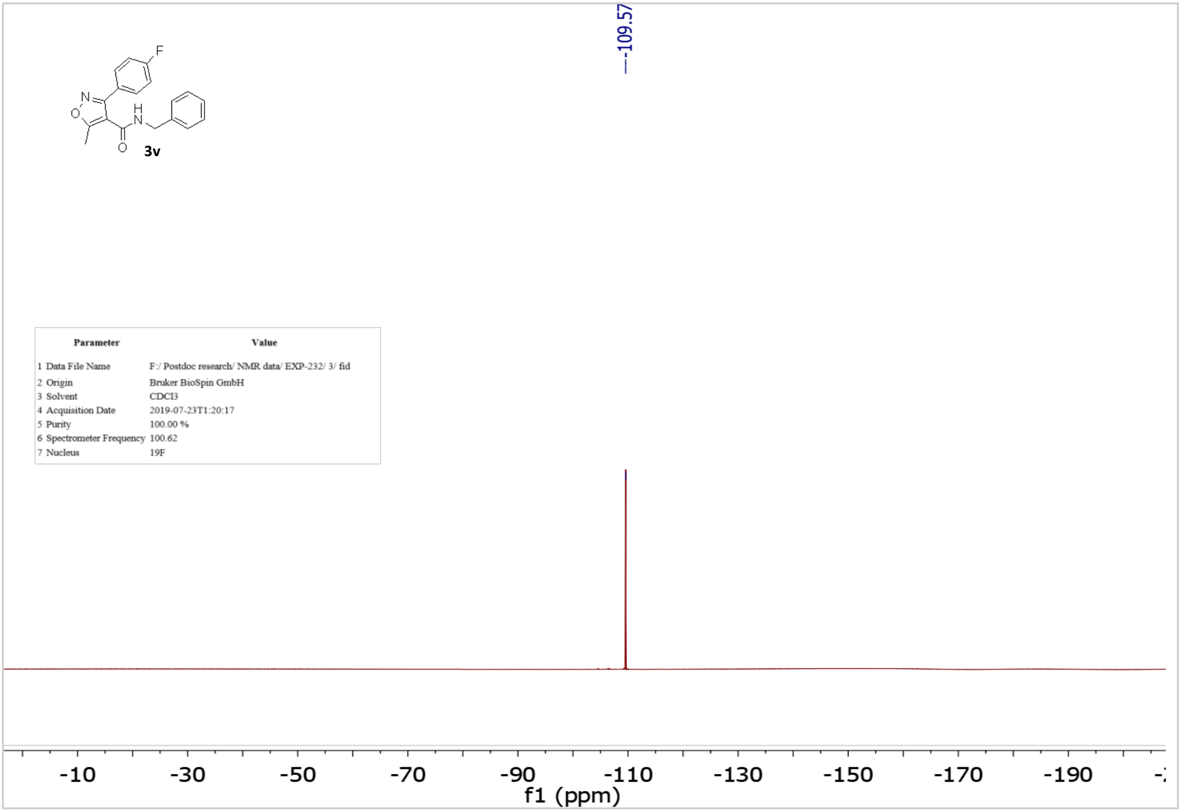

Figure S37: ^{19}F NMR spectrum of compound **3v**.

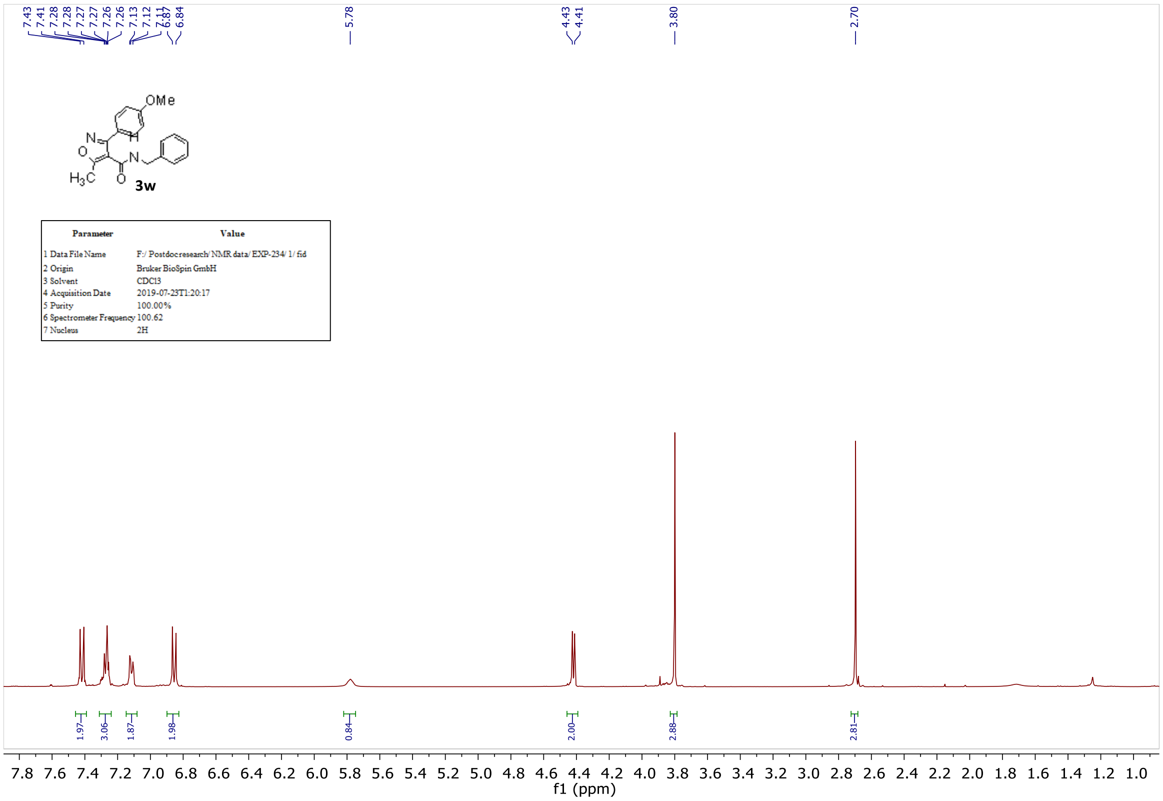

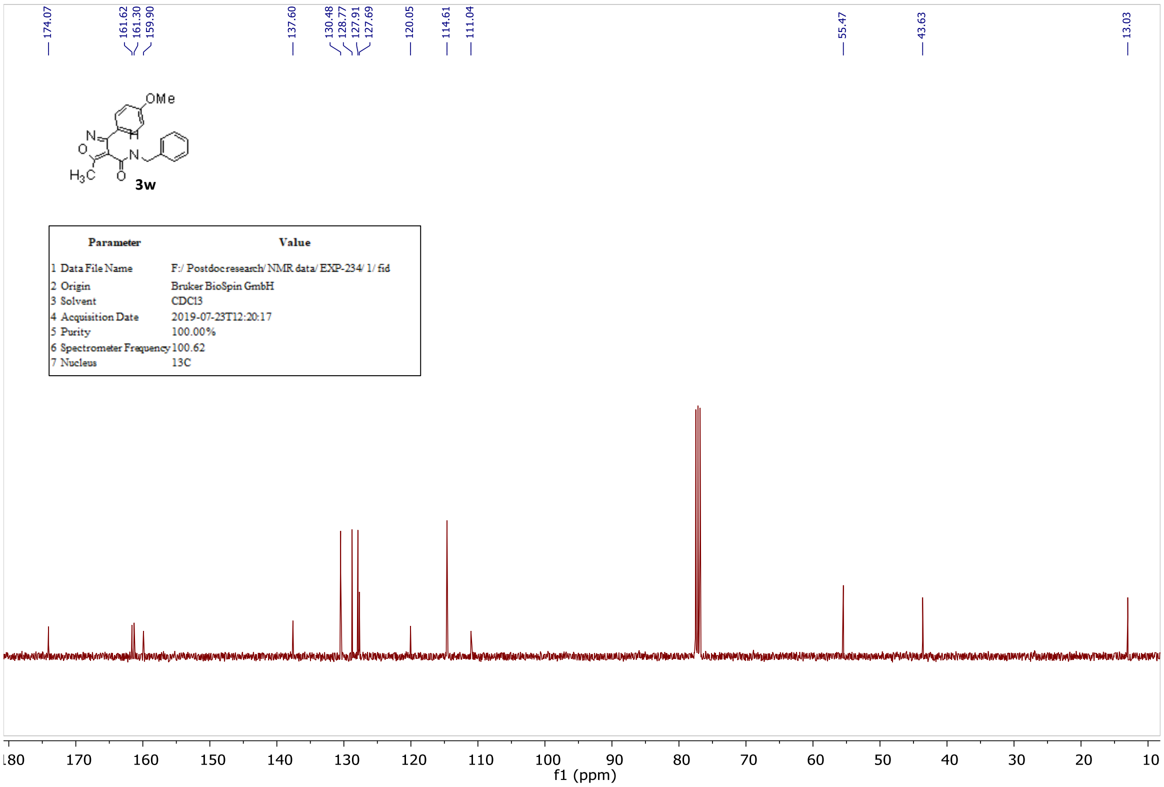

Figure S38: ^{1}H and ^{13}C NMR spectra of compound **3w**.

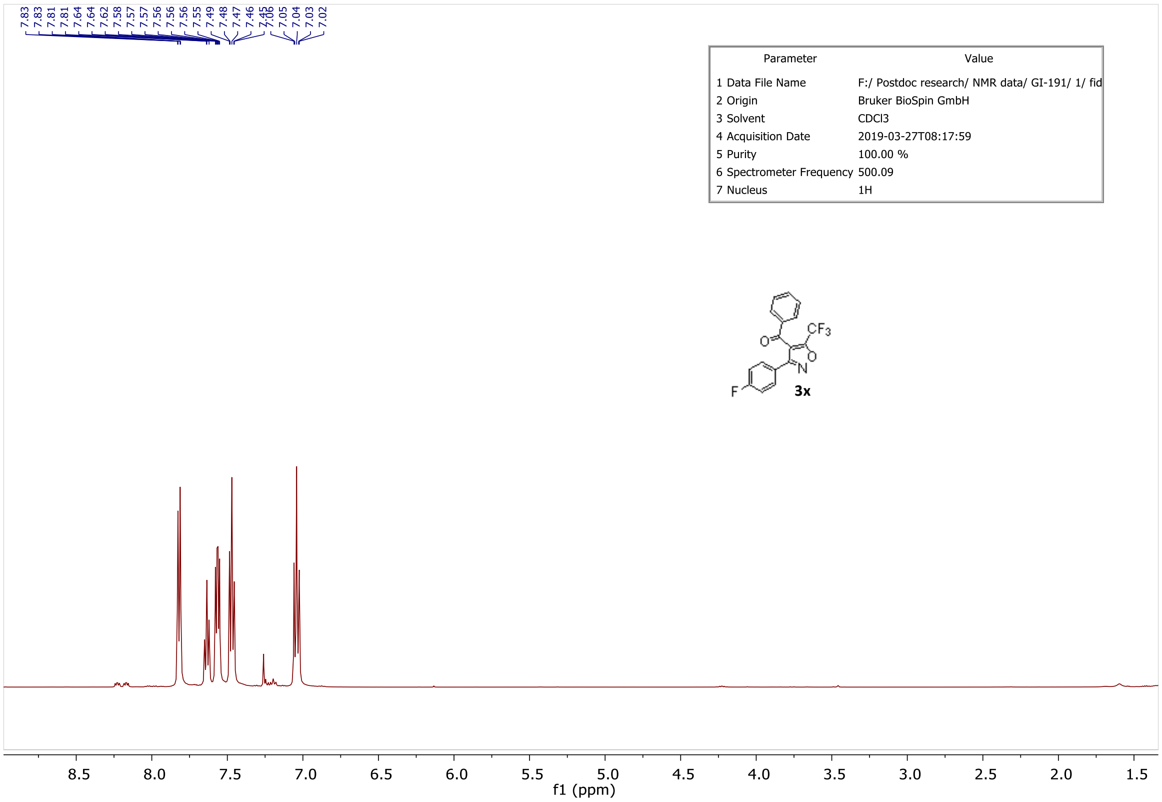

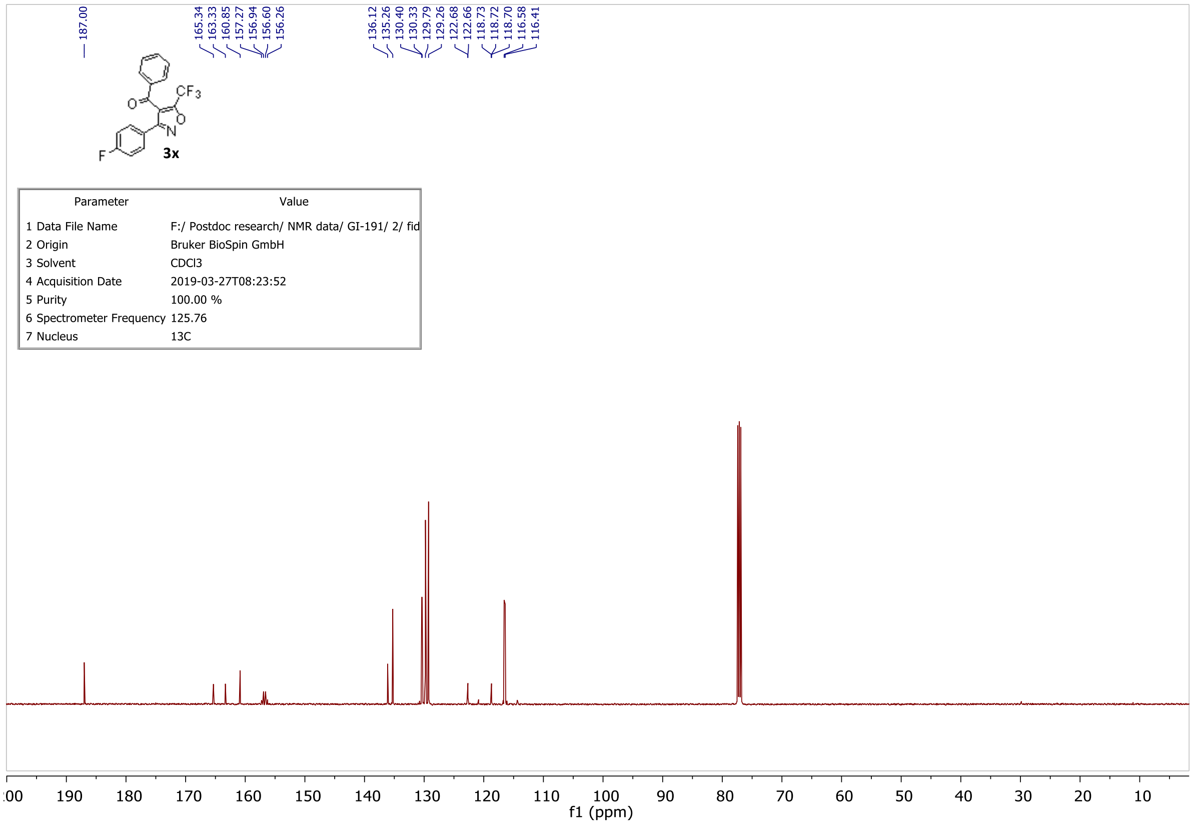

Figure S39: ^{1}H and ^{13}C NMR spectra of compound **3x**.

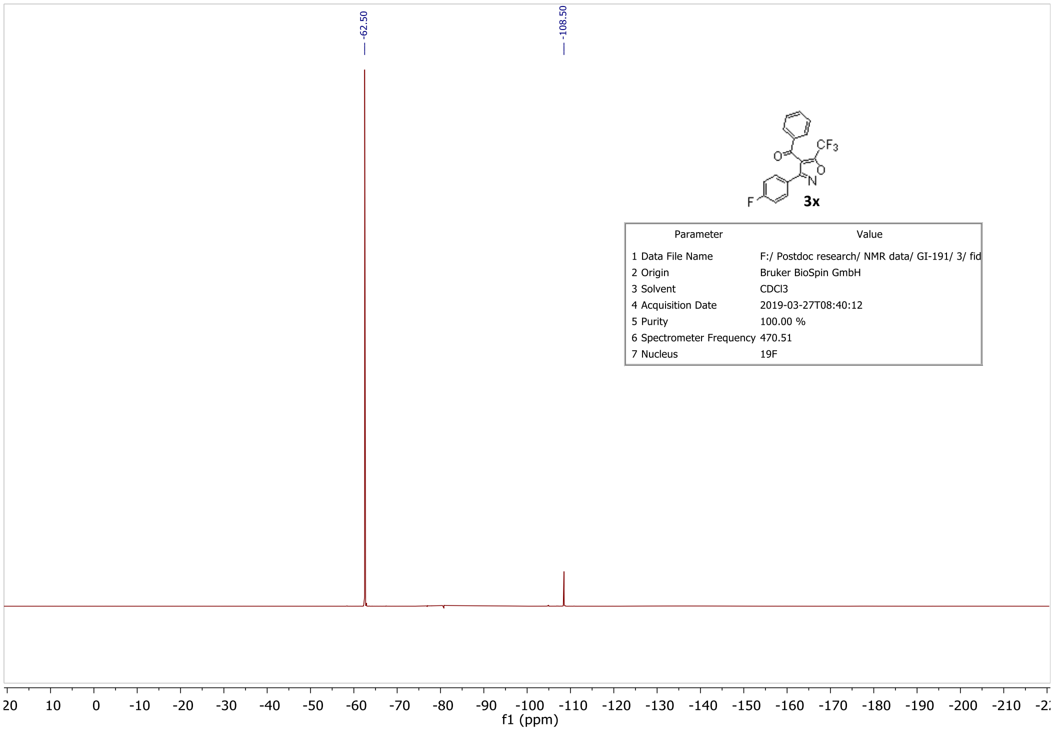

Figure S40: ^{19}F NMR spectrum of compound **3x**.

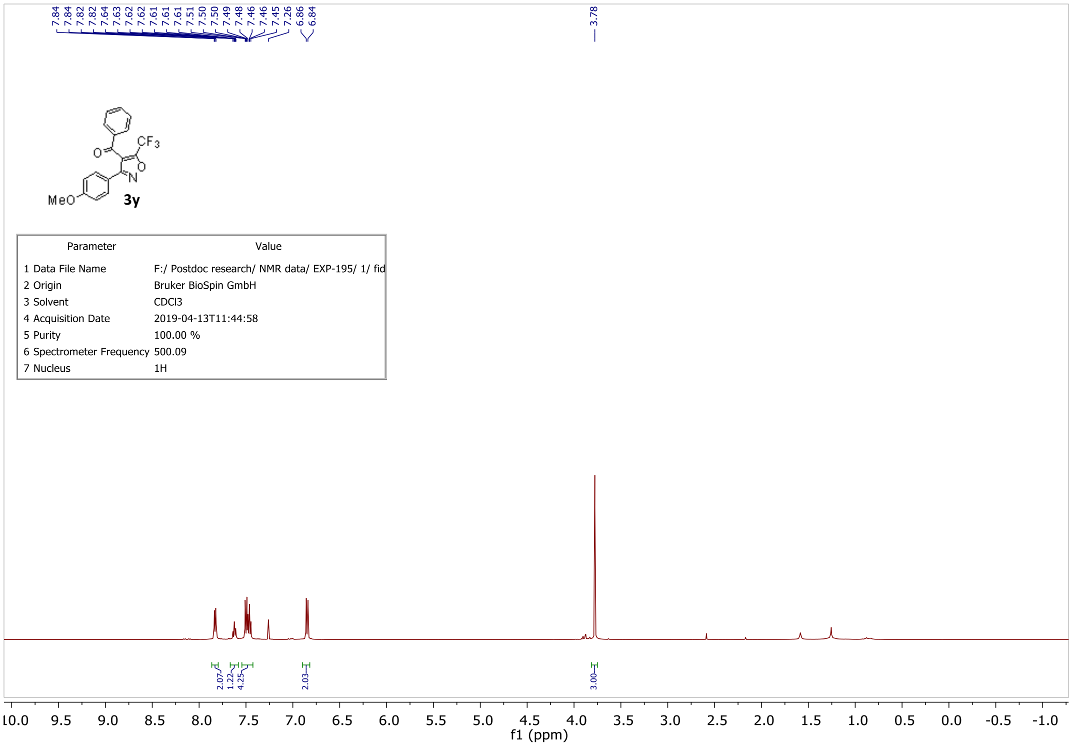

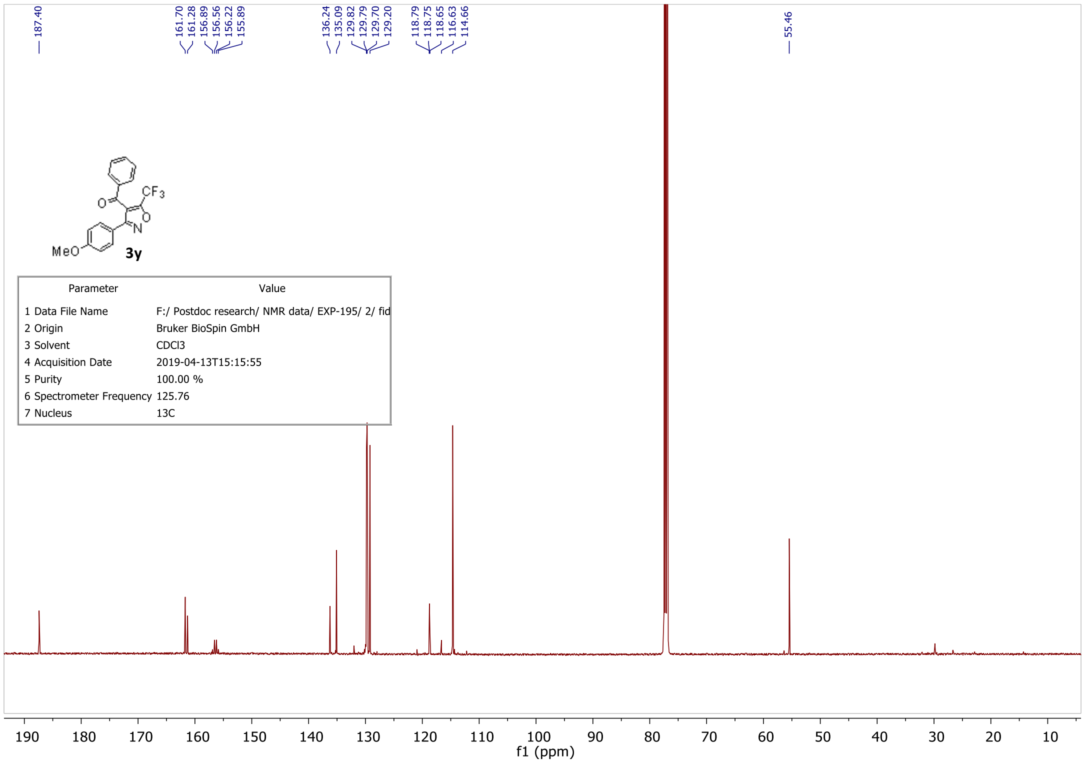

Figure S41: ^{1}H and ^{13}C NMR spectra of compound **3y**.

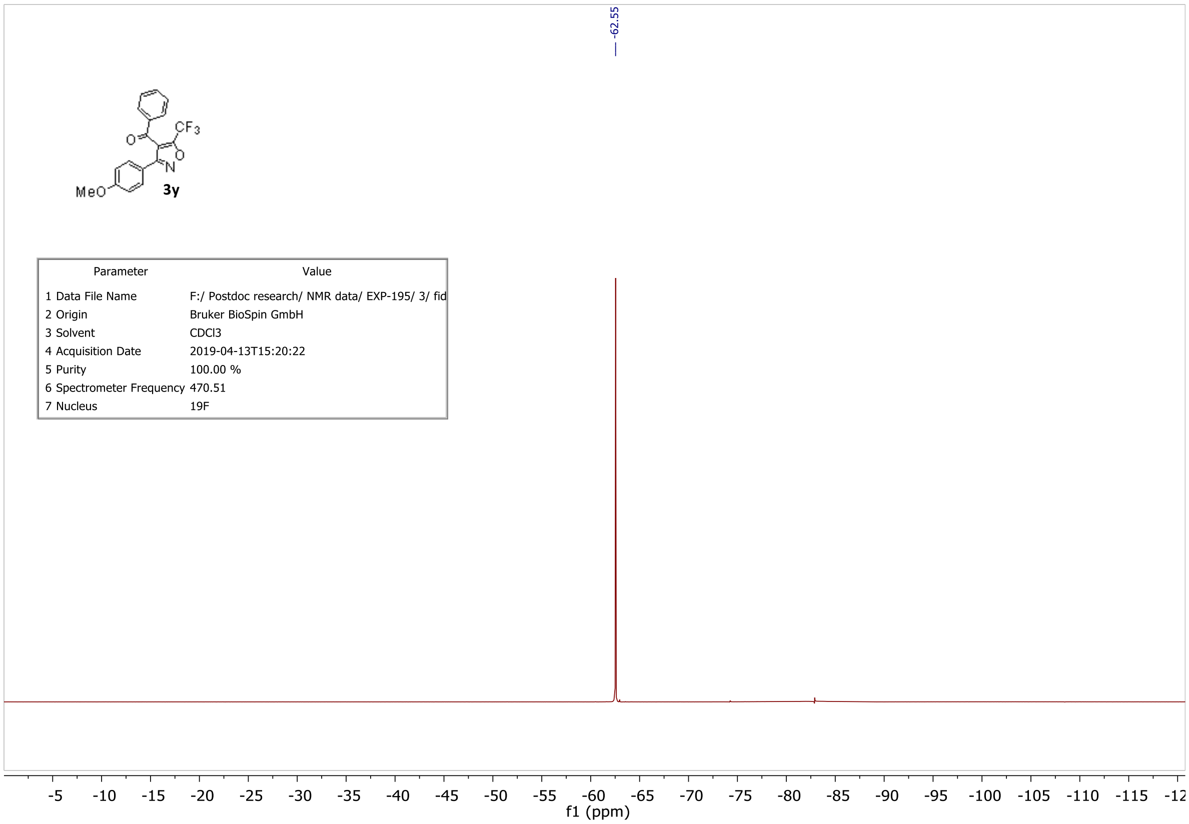

Figure S42: ^{19}F NMR spectrum of compound **3y**.

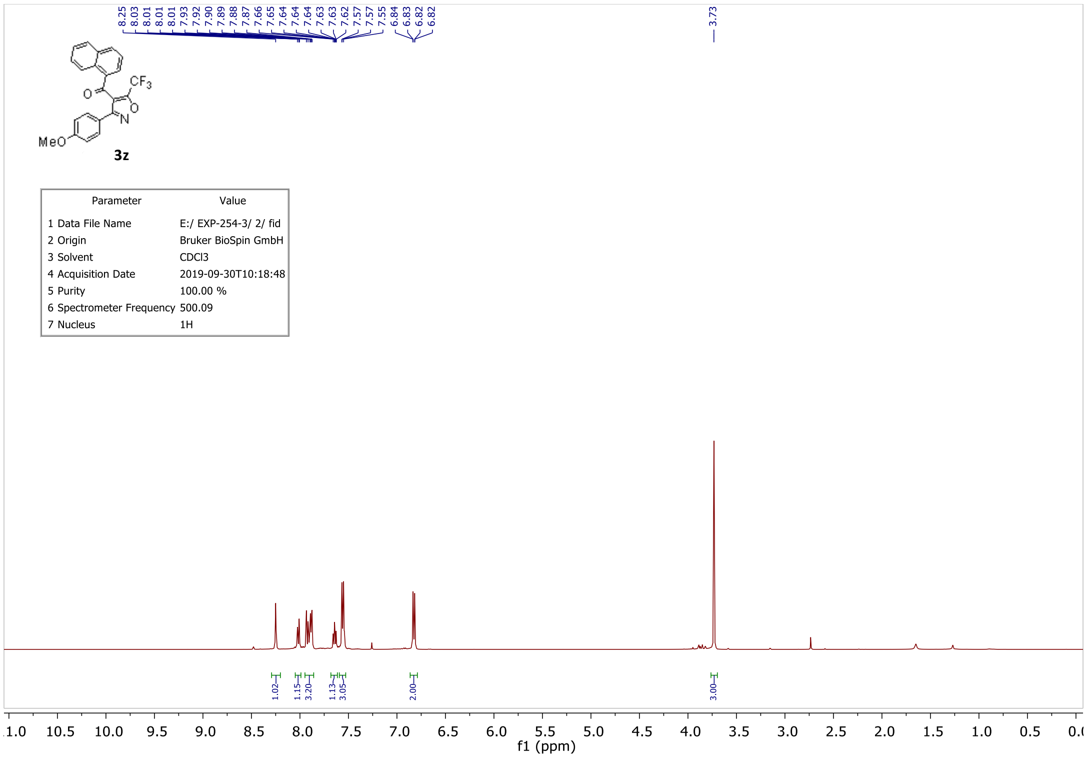

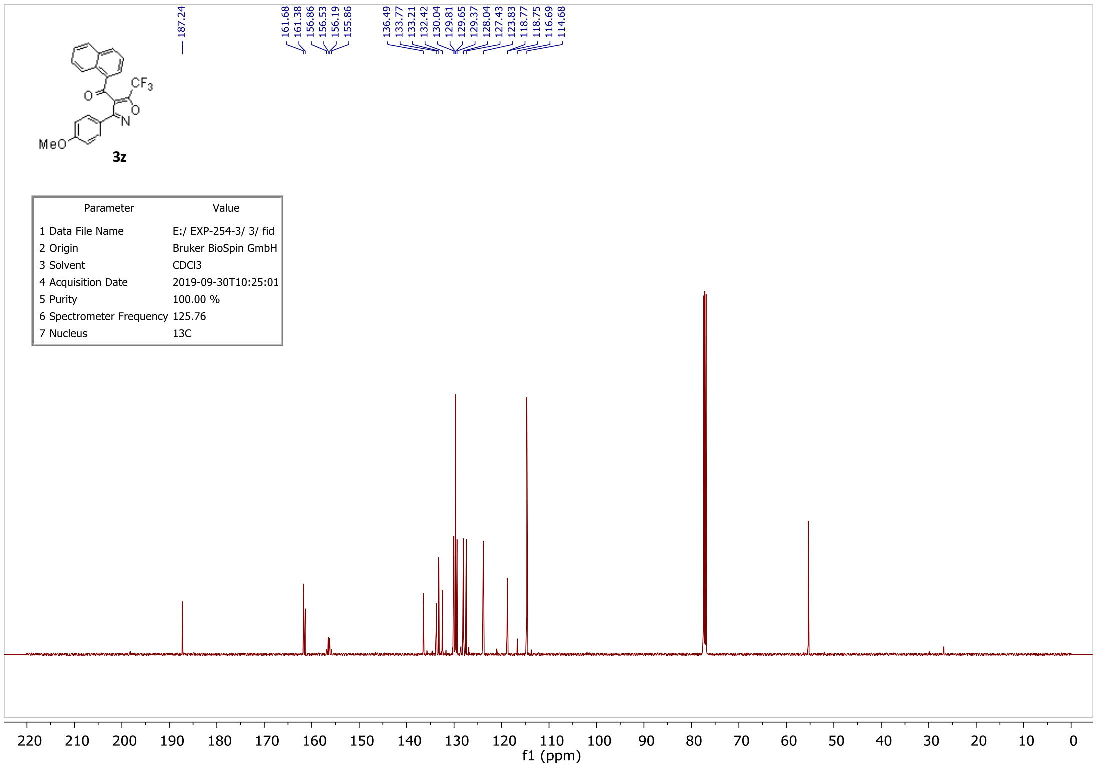

Figure S43: ^{1}H and ^{13}C NMR spectra of compound **3z**.

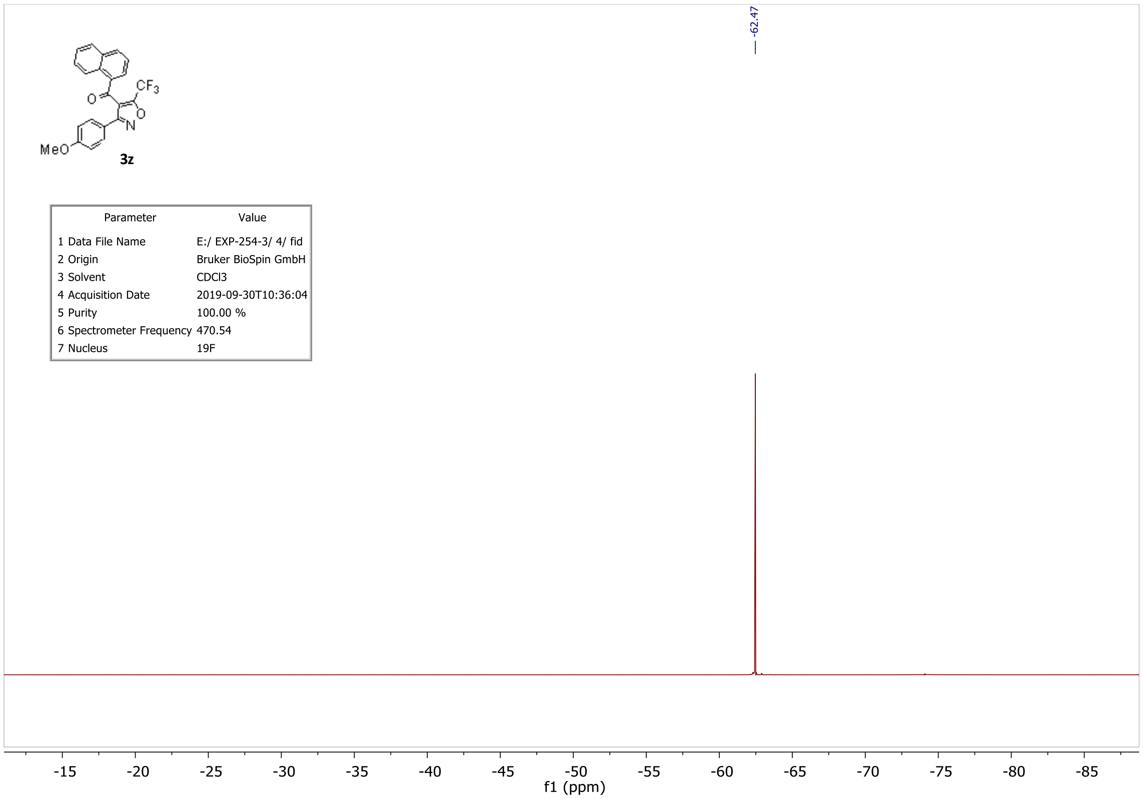

Figure S44: ^{19}F NMR spectrum of compound **3z**.

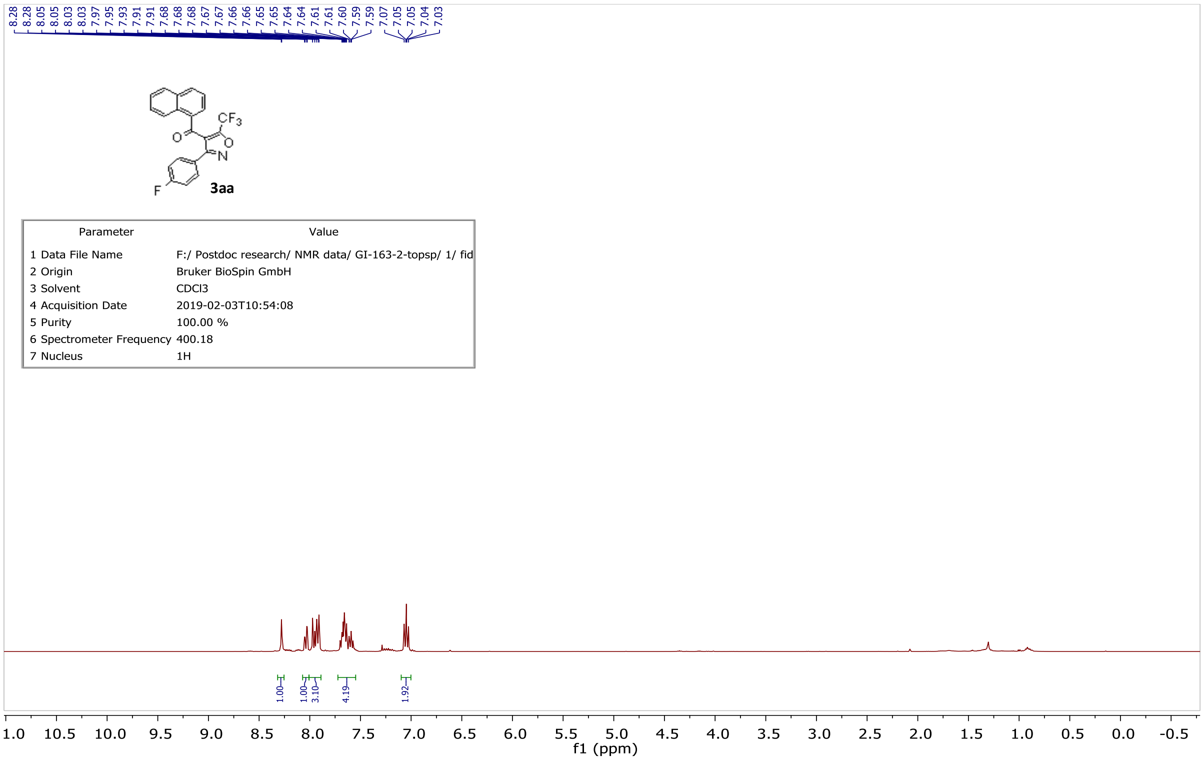

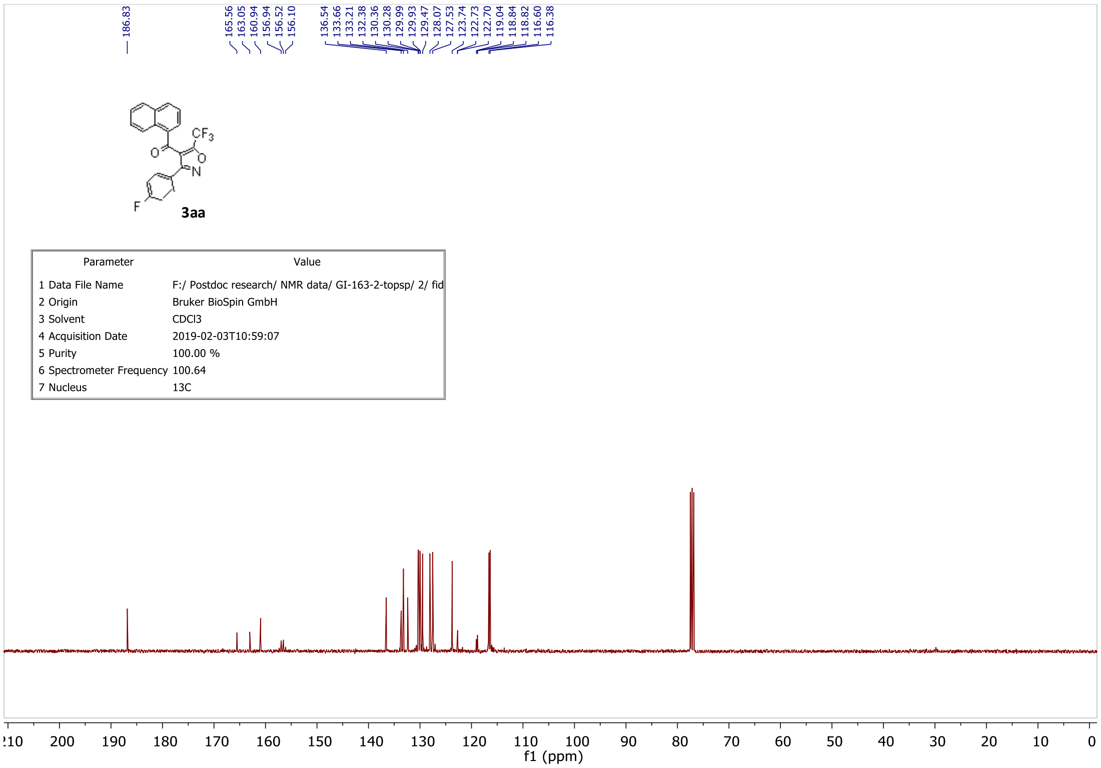

Figure S45: ^{1}H and ^{13}C NMR spectra of compound **3aa**.

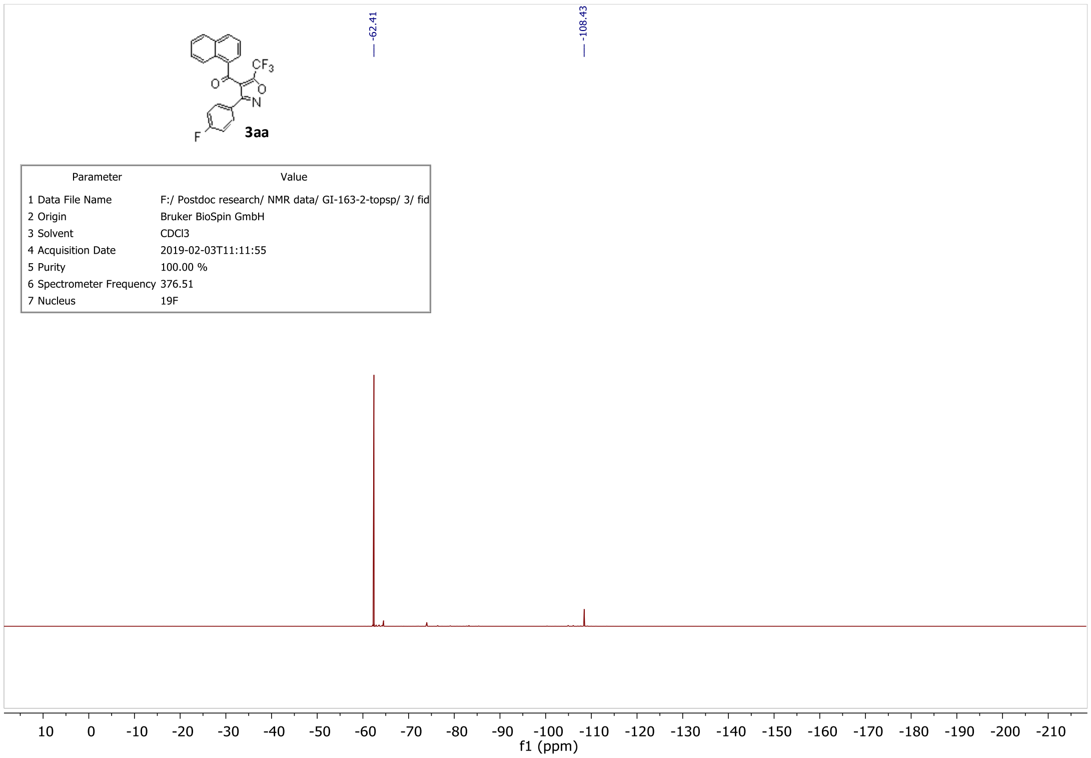

Figure S46: ^{19}F NMR spectrum of compound **3aa**.
